# Pharmacokinetics of antifungal drugs: practical implications for optimized treatment of patients

**DOI:** 10.1007/s15010-017-1042-z

**Published:** 2017-07-12

**Authors:** Romuald Bellmann, Piotr Smuszkiewicz

**Affiliations:** 1 Clinical Pharmacokinetics Unit, Division of Intensive Care and Emergency Medicine, Department of Internal Medicine I, Medical University of Innsbruck, Anichstrasse 35, 6020 Innsbruck, Austria; 2 Department of Anesthesiology, Intensive Therapy and Pain Treatment, University Hospital, Poznań, Poland

**Keywords:** Polyenes, Amphotericin B lipid formulations, Liposomal amphotericin B, Itraconazole, Voriconazole, Echinocandins, Caspofungin, Critically ill, Renal replacement therapy, Extracorporeal membrane oxygenation

## Abstract

**Introduction:**

Because of the high mortality of invasive fungal infections (IFIs), appropriate exposure to antifungals appears to be crucial for therapeutic efficacy and safety.

**Materials and methods:**

This review summarises published pharmacokinetic data on systemically administered antifungals focusing on co-morbidities, target-site penetration, and combination antifungal therapy.

**Conclusions and discussion:**

Amphotericin B is eliminated unchanged via urine and faeces. Flucytosine and fluconazole display low protein binding and are eliminated by the kidney. Itraconazole, voriconazole, posaconazole and isavuconazole are metabolised in the liver. Azoles are substrates and inhibitors of cytochrome P450 (CYP) isoenzymes and are therefore involved in numerous drug–drug interactions. Anidulafungin is spontaneously degraded in the plasma. Caspofungin and micafungin undergo enzymatic metabolism in the liver, which is independent of CYP. Although several drug–drug interactions occur during caspofungin and micafungin treatment, echinocandins display a lower potential for drug–drug interactions. Flucytosine and azoles penetrate into most of relevant tissues. Amphotericin B accumulates in the liver and in the spleen. Its concentrations in lung and kidney are intermediate and relatively low myocardium and brain. Tissue distribution of echinocandins is similar to that of amphotericin. Combination antifungal therapy is established for cryptococcosis but controversial in other IFIs such as invasive aspergillosis and mucormycosis.

## Introduction

Invasive fungal infections (IFIs) are associated with a high morbidity and mortality. *Candida* species, *Cryptococci*, *Aspergilli*, *Mucorales* and other fungi cause life-threatening IFIs mainly in immunocompromised patients. Critically ill patients, particularly those on broad spectrum antibacterial treatment, on renal replacement therapy, total parenteral nutrition, corticosteroids or other immunosuppressives are at risk of candidaemia and other manifestations of invasive candidiasis. Cryptococcosis is a typical opportunistic infection of immunodeficiency resulting from HIV infection. Several endemic fungal infections will also require systemic treatment. Invasive aspergillosis mainly affects patients with haematological malignancies, in particular those with acute myelogenous leukaemia, and patients who have undergone haematopoietic stem cell transplantation. Solid organ transplant recipients are another susceptible population. Critically ill patients suffering from severe liver cirrhosis or advanced chronic obstructive pulmonary disease have also an enhanced risk of acquiring invasive aspergillosis [1, 2]. Typical risk factors for mucormycosis comprise immunosuppression, diabetes, blood transfusion and treatment with chelators. Immediate aggressive antifungal treatment is crucial for the outcome of IFIs. As the diagnosis is difficult and often delayed empirical or pre-emptive antifungal therapy is indicated in many cases. Patients at highest risk of IFI, e.g. those with prolonged neutropenia after induction chemotherapy for acute myelogenous leukaemia or myelodysplastic syndrome or those receiving aggressive immunosuppression for graft versus host disease after haematopoietic stem cell transplantation, require antifungal prophylaxis. Comprehensive guidelines for the management of the most prevalent IFIs are available.

A timely and sufficiently high exposure to the appropriate antifungal agent is crucial for eradication of the pathogen. Most of the patients with IFIs, however, suffer from severe underlying diseases and various co-morbidities resulting in enhanced vulnerability to adverse drug reactions. Furthermore, co-morbidities can affect absorption, distribution, metabolism and elimination of antifungals and other essential drugs. Gastro-intestinal impairment, e.g. caused by anticancer chemotherapy or impaired gastro-intestinal perfusion may affect absorption of orally administered azoles or flucytosine resulting in sub-therapeutic exposure. Metabolism and elimination may be altered by impaired hepatic and renal function. In critical illness, typical pathophysiological changes such as altered hydration and haemodynamics, tissue perfusion and plasma protein levels may influence drug distribution [3]. Pharmacodynamic and pharmacokinetic drug–drug interactions involving antifungals are common as the vast majority of patients with IFIs suffer from co-morbidities and receive concomitant medications. Extracorporeal organ support can affect drug distribution and elimination. Pharmacokinetics in these special patient groups may therefore be largely different from that in healthy subjects or in less compromised patients. Appropriate dosing of antifungal is challenging under these special conditions as respective pharmacokinetic data is sparse or even lacking.

Concerning their pharmacodynamic properties, antifungals are categorised as fungistatic (azoles, 5-flucytosine, echinocandins on *Aspergilli*) or fungicidal (amphotericin B, echinocandins on *Candida*). For azoles, 5-flucytosine, and echinocandins, the ratio between the area under the concentration–time curve (AUC) and the minimal inhibitory concentration (MIC) of the causative fungal pathogen (AUC/MIC) best correlates with antifungal efficacy. By contrast, amphotericin B is a concentration-dependent antifungal agent displaying a relevant post-antifungal effect. Thus, the ratio between its peak concentration (*C*_max_) and the MIC of the fungus (*C*_max_/MIC) is the relevant pharmacokinetic/pharmacodynamic parameter [4]. Target values for these parameters are derived from animal models. By pharmacokinetic/pharmacodynamic modelling and Monte Carlo simulations, the probability of target attainment (PTA) was assessed for different antifungals under various clinical conditions.

For IFIs localized outside the bloodstream, target-site kinetics of antifungals are a key issue in treatment [5]. Until now, the majority of data originate from tissue homogenates obtained in animal studies. Only limited data are available from tissue biopsies, samples taken at surgery or autopsy, and from body fluids such as cerebrospinal fluid (CSF), peritoneal fluid, or pleural effusion. Drug target-site penetration is frequently expressed by ratio between tissue (target-site) concentration and the simultaneous plasma level. The discordance of the shape of target-site and plasma concentration–time profiles, however, which is termed hysteresis, can lead to incorrect estimation of drug penetration when single measurements are performed. By comparison of the area under the concentration–time curves (AUC) at target site and in plasma more representative data can be obtained. This approach, of course, requires the measurement of multiple target site and simultaneous plasma concentrations [5]. Pharmacokinetic/pharmacodynamic modelling has also been performed with target-site concentrations.

Taking into account pharmacokinetic/pharmacodynamic characteristics and mechanisms of action of antifungal agents, the combined antifungal therapy (CAF) exhibits differentiated drug–drug interactions (synergism, additivity, indifference, antagonism) as well as variable effectiveness in different tissues. Several models have been established to explain the mechanisms behind these effects. CAF has been investigated in several systematic clinical studies. For rare conditions, there are case reports on CAF. At the time, a few indications for CAF are supported by current guidelines.

The objective of this review is to summarise clinically relevant knowledge on pharmacokinetics of antifungals currently used for treatment of IFIs. We focus on special clinical conditions, e.g. critical illness, renal and hepatic impairment, on the implications for choice and dosage of antifungals and on the controversial field of CAF.

## Amphotericin B

Amphotericin B has been introduced in therapy in 1958 [6]. It comprises an amphophilic, monocyclic polyene lactone ring which is linked to mycosamine. Its solubility in water and in most organic solvents is poor [7]. Its molecular weight amounts to 924 Da. The so-called conventional form of amphotericin B is a deoxycholate formulation forming micelles in aqueous solution [6].

Three mechanisms of action have been described for amphotericin B. First, eight molecules of amphotericin B interact with eight ergosterol molecules and form channels. Two of such channels assemble forming a membrane-spanning pore. As a consequence, the loss of essential low-molecular-weight substrates such as electrolytes results in death of the fungal cell. Lipid peroxidation and inhibition of the fungal proton-ATPase are further cytotoxic mechanisms of amphotericin B [8]. Amphotericin B is active against the majority *Aspergillus* species*, Absidia species, Basidiobolus* species*, Blastomyces dermatitidis, Candida* species*, Coccidioides immitis, Conidiobolus* species*, Cryptococcus neoformans, Histoplasma capsulatum, Mucor* species*, Paracoccidioides* species*, Rhizopus* species*, Rhodotorula,* and against *Sporothrix schenckii* [9]. Many *A. terreus* strains, however, are resistant to amphotericin B. Because of its broad antifungal spectrum, amphotericin B is still an important drug for the treatment of invasive aspergillosis as well as non-aspergillus mould infections [10, 11]. According to current guidelines, it is the drug of choice for *Candida* meningoencephalitis, *Candida* endocarditis and urinary tract infections caused by fluconazole-resistant *Candida* [12, 13]. Recent epidemiological data from 11 Italian centres revealed amphotericin B susceptibility of all clinical *Candida* isolates [14].

### Adverse effects of amphotericin B

The use of amphotericin B is limited by numerous adverse effects. Infusion-related adverse events (IRAE) comprise chills, rigors, fever, hypotension or hypertension, hypoxia, nausea, vomiting, and hypokalaemia sometimes resulting ventricular fibrillation. About 50% of the patients on treatment with conventional amphotericin B deoxycholate are affected by IRAE. Probably, pro-inflammatory cytokines and immunostimulation via Toll-like receptors (TLRs) are involved in IRAE [15–17].

Deterioration of renal function with an increase in serum creatinine is observed in as many as 80% of patients on treatment with amphotericin B deoxycholate. In about 40%, doubling of baseline creatinine is reported [16, 18–26]. The renal toxicity is caused by vasoconstriction of the afferent arteriole resulting in a reduction of renal blood flow and glomerular filtration rate combined with tubular injury resulting in loss of potassium, magnesium, bicarbonate, and amino acids. A daily dose of >35 mg/d, a body weight >90 kg, male sex, simultaneous administration of nephrotoxic medications such as aminoglycosides or cyclosporine A are risk factors for renal adverse effects [15]. Rarely, anaemia and haemolysis have been observed during amphotericin B treatment. Whereas hypokalaemia is a common adverse effect of amphotericin B, excessive hyperkalaemia with cardiac arrest has also been observed [27].

### Dosage, plasma pharmacokinetics, and administration of amphotericin B deoxycholate

All commercially available amphotericin B formulations have to be administered by intravenous infusion, because their enteral absorption is negligible. In plasma, 95–99% of amphotericin B is protein-bound, mainly to LDL, albumin and α-1-acid glycoprotein [28, 29]. Infusion of a 1-mg test dose prior to the therapeutic dose is recommended to identify patients who are intolerant. Subsequently, 0.25–0.3 mg/kg once daily should be applied and the daily dose should be increased by 5–10 mg per day until the maintenance dose of 0.6–1.0 mg/kg once daily is reached. For eradication of highly resistant fungi, a dose up to 1.5 mg/kg per day might be considered. Prolonged infusion over ≥6 h is particularly important in these cases. After administration of amphotericin B deoxycholate, amphotericin B is eliminated from plasma with a half-life (*t*_½_ β) of ~24 h and a clearance of 10 to ~30 ml/kg/h. Its apparent volume of distribution (*V*_d_) is 0.5–2.0 L/kg. The peak level (*C*_max_) was ~2 µg/mL after standard doses of ~1 mg/kg body weight. An infusion time (*T*_inf_) of ≥4 h is required to warrant tolerability [30–34]. Twenty percent of labelled amphotericin B have been detected in the urine and ~40% in the faeces within a week after administration. This is probably unchanged amphotericin B because no amphotericin B metabolism has been detected so far (see Table 1) [28, 35–37].

**Table 1:** Overview on pharmacokinetics of amphotericin B preparations

Preparation	Amphotericin B deoxycholate	Liposomal amphotericin B AmBisome^®^
*C*_max_ (µg/mL)	1.7–2.8	14–29 (90)
AUC (µg h/mL)	14–29	423
*V*_d_ (L/kg)	0.5–2.0	0.05–2.2
Protein binding (%)	95–99	95–99 (of amphotericin B, liberated from lipid encapsulation)
*t*_1/2_ (h)	15–27	13–24
CL (mL/h/kg)	10–30	1–23
Elimination	Bile, kidney; no metabolites identified	Bile, RES long-term disposition, final elimination not yet clear; no metabolites identified
Renal impairment	Contra-indicated in reversible renal impairment	No dose adjustment, consider nephrotoxicity
Hepatic impairment	No dose adjustment, consider hepatotoxicity and renal toxicity	No dose adjustment, consider hepatotoxicity
Remark	*T*_inf_ ≥ 4 h mandatory, continuous infusion reduces toxicity, but may decrease the efficacy	*T*_inf_ ≥ 4 h recommended

Details and references are displayed in the text

*C*_max_ amphotericin B peak level; *AUC* total area under the concentration–time curve; *V*_d_ apparent volume of distribution; *t*_1/2_ half-life; *CL* clearance; *RES* reticuloendothelial system; *T*_inf_ infusion time

### Continuous infusion of amphotericin B deoxycholate

As high amphotericin B peak concentrations appear to correlate with its toxicity continuous infusion has been tried to enhance its tolerability. This approach was first reported by Chabot et al. [38]. Later on, 5-h infusion of amphotericin B deoxycholate was compared with continuous infusion in a randomized open-label trial. IRAE were significantly less frequent, and the increase in serum creatinine was lower in the continuous infusion group. The mortality was significantly lower in patients on continuous amphotericin B infusion (0 versus 18% at the end of treatment, 10 versus 30% after a 3-month follow up) [22]. In a retrospective study, renal deterioration as defined by a doubling of serum creatinine has been investigated. Treatment efficacy was a second endpoint. A median increase in serum creatinine by 50 and 85% was found in patients on continuous infusion and in patients on 4-h infusions, respectively. Renal impairment was significantly less frequent in patients who had received amphotericin B as a continuous infusion (*P* < 0.001). Fourteen-day survival was 95% in the continuous infusion cohort and only 79% in the group on 4-h infusion (*P* = 0.03) [39]. In addition, several observational studies on continuous amphotericin B infusion have been performed. In six patients who had undergone lung transplantation and obtained amphotericin B by continuous infusion (1 mg/kg/day, 40 days on average) and nephrotoxic co-medication (cyclosporine A, aminoglycosides, and ganciclovir), there was a median decline in serum creatinine clearance from 57 to 35 mL/min. One patient transiently required hemofiltration. However, renal function recovered after amphotericin B treatment had been stopped. [40]. The calculated creatinine clearance was retrospectively analysed in allogeneic hematopoietic stem cell recipients under immune-suppression with cyclosporine A on and off amphotericin B administered by continuous infusion. Creatinine clearance was 55 mL/min in patients on and 69 mL/min patients without amphotericin B treatment (*P* = 0.0002) [41]. Several case reports and observational studies advocate the administration of amphotericin B as a continuous infusion to enhance its tolerability [42–44]. Quite different results were obtained by Maharom and Thamlikitkul from 148 patients undergoing 166 treatment courses. Amphotericin B had been administered by continuous infusion in 61.4%. Infusion-related toxicity was less frequent in patients on continuous infusion. Renal toxicity was also somewhat lower in this group, but the difference was not significant. Surprisingly, the mortality was significantly higher in patients on continuous infusion compared to those who had obtained intermittent infusions (*T*_inf_ 4–6 h) [44]. For explanation of these conflicting results, two effects on mortality have to be considered. First, renal failure is associated with an enhanced mortality. Improved renal tolerability of amphotericin B treatment by continuous might therefore results in a better survival. Second, the mortality of IFI will depend on the efficacy of fungal eradication, and as mentioned, the ratio *C*_max_/MIC is supposed to correlate best with the antifungal activity of amphotericin B. For optimal efficacy against *A. fumigatus*, *C*_max_/MIC >2.4 has been suggested [45]. From a pharmacodynamic point of view, intermittent administration might therefore be advantageous. Whether antifungal efficacy of amphotericin is adequate with continuous infusion remains to be clarified [37].

### Amphotericin B deoxycholate in special patient groups

Nowadays, amphotericin B deoxycholate is contra-indicated in acute renal failure as less nephrotoxic antifungals are available. However, its use is possible in terminal renal failure requiring renal replacement therapy. Relatively small doses of 25–50 mg have been applied during intermittent hemodialysis three times per week. Since nephrotoxicity has no impact under this condition, the standard dosage appears to be appropriate in hemodialysis patients suffering from life-threatening fungal infections. Continuous veno-venous hemofiltration performed in two critically ill patients with terminal renal failure on amphotericin B deoxycholate treatment appeared to accelerate the amphotericin B clearance [46]. Recently, relatively low *C*_max_ and large *V*_d_ values were reported from critically ill patients [47].

### Lipid formulations of amphotericin B

Lipid encapsulation is another approach to improve tolerability of amphotericin B. Several preparations have been developed and assessed in preclinical and clinical studies. Three formulations with different chemical composition, particle size and shape have been launched: Liposomal amphotericin B (AmBisome^®^, Gilead, Dublin, Ireland), amphotericin B colloidal dispersion (colloidal amphotericin B, Amphotec^®^, Amphocil^®^, Ben Venue Laboratories, Bedford, Ohio, USA), and amphotericin B lipid complex (Abelcet^®^, Sigma-Tau Pharma Source, Inc., Indianapolis, IN). Today, liposomal amphotericin B is the only widely available lipid formulation. Liposomal amphotericin B consists of spherical uni-lamellar vesicles (liposomes) of 45–80 nm in diameter containing hydrogenated soy phosphatidylcholine, cholesterol, distearoyl phosphatidylglycerol, and amphotericin B in a molecular ratio of 2:1:0.8:0.4 [48]. The production of amphotericin B colloidal dispersion, a cholesteryl sulphate complex of amphotericin B, has been stopped in 2012 [49]. The lipid moiety of amphotericin B lipid complex consists of l-alpha-dimyrsitoylphosphatidylcholine, l-alpha-dimyrsitoylphosphatidylglycerol forming ribbon-like structures, 1600–11,000 nm in length [50].

### Dosage and plasma pharmacokinetics of lipid-formulated amphotericin B

The recommended standard doses of lipid-formulated amphotericin B are much higher than that of conventional amphotericin B: for liposomal amphotericin B, it is 3–4 mg/kg per day (5 mg/kg for mucormycosis, even 10 mg/kg, for *Mucorales* infections of the CNS). For amphotericin B lipid complex, the standard dose amounts to 5 mg/kg once daily. Amphotericin B lipid formulations display marked differences in their pharmacokinetics [36]. After repeated administration of 5 mg/kg/d of liposomal amphotericin B, amphotericin B peak levels as high as 90 µg/mL were measured [51–53]. A *t*_1/2_ of 5–10 h was determined in most of the studies (see Table 1). This is shorter than that observed during treatment with amphotericin B lipid complex. Liposomal amphotericin B has a relatively small volume of distribution of ~0.1–0.2 L/kg (see Table 1), that of amphotericin B lipid complex is highly variable and very large (up to 131 L/kg) [54, 55]. *C*_max_ values of 2 µg/mL are reached with amphotericin B lipid complex at standard doses. Liposomal amphotericin B and amphotericin B lipid complex display non-linear pharmacokinetics [32, 52, 54, 56]. Unlike lipid-encapsulated amphotericin B, the liberated fraction displayed quite similar pharmacokinetics after administration of different lipid formulations [46, 57].

### Lipid-formulated amphotericin B in special patient groups

In critically ill patients, lower amphotericin B plasma levels were achieved by liposomal amphotericin B than in healthy subjects or in less compromised patients [34, 46, 58]. Continuous veno-venous haemofiltration, haemodiafiltration and intermittent haemodialysis, did not significantly affect exposure to liposomal amphotericin B [34, 46, 58, 59]. CL and AUC_0–24 h_ of liberated amphotericin B which is looked upon as the active amphotericin B fraction were not significantly different on and off hemofiltration. Standard dosage is, therefore, probably adequate during continuous renal replacement therapy [46]. This is also true for amphotericin B colloidal dispersion and amphotericin B lipid complex [46, 60]. Amphotericin B lipid complex, however, should be avoided in patients with renal impairment because of its nephrotoxicity [54]. Cholestatic liver disease had no significant influence on steady state pharmacokinetics of liberated amphotericin B when amphotericin B colloidal dispersion was administered [61]. In three patients treated with albumin dialysis for cholestatic liver failure who received lipid-formulated amphotericin B (one patient liposomal amphotericin B, one amphotericin B colloidal dispersion and one patient amphotericin B lipid complex), exposure with liberated amphotericin B was slightly decreased. However, a dose adjustment of lipid-formulated amphotericin B for albumin dialysis is probably not necessary [62, 63]. In a patient on extracorporeal membrane oxygenation (ECMO), amphotericin B levels were measured 13 and 18 h after administration of liposomal amphotericin B at a dose of 3 mg/kg. Levels were within the therapeutic range with 5.8 and 6.2 µg/mL, respectively [64].

### Safety and antifungal activity of amphotericin B lipid formulations

The antifungal activity of lipid-formulated amphotericin B at doses of 3–5 mg/kg is comparable to that of 0.6–1.0 mg/kg of amphotericin B deoxycholate [34, 50, 65–77]. At these standard doses, the amphotericin B lipid formulations are less toxic than amphotericin B deoxycholate. The underlying mechanisms are not yet completely understood. Suggested explanations comprise targeting to fungal cell surface with minimal systemic exposure to free amphotericin B as well as different binding to plasma lipoproteins and rapid uptake by the reticuloendothelial system (RES) [67, 75, 77–84]. However, considerable amounts of amphotericin B are liberated from lipid encapsulation in the plasma of healthy subjects and patients [28, 46, 57]. Reduced concentrations of free amphotericin B may probably play a role [36, 57].

### Target-site penetration of amphotericin B preparations

Tissue penetration of amphotericin B was studied in human autopsy material of patients who had received amphotericin B deoxycholate. There was an accumulation of amphotericin B in liver and spleen. Concentrations were intermediate in lung and kidney and low in myocardium and brain [78, 85]. After treatment with amphotericin B lipid formulations, amphotericin B target-site distribution in autopsy samples was similar to that reported after amphotericin B deoxycholate with tissue levels of ~100 µg/g in the liver and lowest concentrations in myocardium and cerebral cortex (~1 µg/g) [86]. In a preclinical study, enhanced cerebral amphotericin B uptake was achieved by exposure to P-glycoprotein (P-gp) inhibitors verapamil and itraconazole [87]. In P-gp knock-out mice, however, brain concentrations of amphotericin B were low [88].

In pulmonary epithelial lining fluid (ELF), amphotericin B levels were much lower than those in whole lung tissue (~0.4–1.6 µg/mL) [89]. Even lower amphotericin B concentrations have been recovered from pleural effusion and from ascites during treatment with different amphotericin B formulations [90–92]. Biliary excretion of amphotericin B appears to depend on the administered formulation. Biliary concentrations of ~5 and 41 µg/mL were achieved with amphotericin B deoxycholate therapy in a patient suffering from *C. albicans* cholecystitis and in a cancer patient, respectively. The *C*_max_ values in plasma were 1.1 and 1.64 µg/mL, respectively. In the cancer patient, switching to amphotericin lipid complex resulted in a biliary *C*_max_ of 60 µg/mL [93, 94]. More recently, biliary amphotericin B levels in liver transplant recipients on treatment with lipid-formulated amphotericin B were assessed. Biliary concentrations were much lower with a maximum of 1.28 µg/mL. In addition, bile displayed an inhibitory effect on antifungal activity of amphotericin B [95].

## Flucytosine

Flucytosine (5-flucytosine, 5-fluorocytosine, Ancotil^®^, ICN Pharmaceuticals Ltd., Cedarwood, Hampshire, UK) is available for systemic treatment of fungal infections since 1968 [96]. It is licensed for the treatment of systemic cryptococcosis, candidiasis, chromomycosis and infections due to *Torulopsis glabrata* and *Hansenula*. For therapy of *Candida* sepsis and *Cryptococcus* meningitis, it is applied in combination with amphotericin B. 5-flucytosine is a prodrug which is converted to 5-fluorouracil its active form by cytosine deaminase inside the fungal cell. Cytosine permease localized in the fungal cell membrane is required for internalization of 5-flucytosine into the fungus. Therefore, a lack of cytosine permease or cytosine deaminase renders resistance to 5-flucytosine. 5-fluorouracil is converted into 5-fluorouridine monophosphate (FUMP), 5-fluorouridine diphosphate (FUDP) and finally into 5-fluorouridine triphosphate (FUTP). FUTP is incorporated into the fungal RNA instead of uridine triphosphate (UTP) causing inhibition of fungal protein synthesis. In addition, fluorodeoxyuridine monophosphate (FdUMP) formation is catalysed by the uridine monophosphate pyro-phosphorylase. FdUMP inhibits the fungal thymidylate synthase and thus fungal DNA synthesis. Flucytosine is active against *Candida* species, *Cryptococcus neoformans, Cladophialophora carrionii, Fonsecaea* species and *Phialophora verrucosa* [97]. As resistance is a common problem in 5-flucytosine therapy, it should be used in combination with other antifungals, mainly with amphotericin B [97].

Flucytosine displays significant adverse effects, in particular hepatotoxicity and myelotoxicity which is probably due to toxic fluorouracil plasma concentrations. Obviously, 5-flucytosine converts spontaneously into 5-fluorouracil. This conversion may be promoted by the gut flora [97].

### Dosage and plasma pharmacokinetics of flucytosine

Flucytosine is available for oral and for intravenous administration. The standard dose recommended by the manufacturer is 100-150 mg/kg per day (25–37.5 mg/kg four times per day, *T*_inf_ = 30 min). Its oral bioavailability amounts to 76–89% [98]. Flucytosine is hydrophilic and has a low protein binding of 3–4% [99]. It is eliminated by about 90% via glomerular filtration with a *t*_1/2_ of 3–4 h [99, 100]. A volume of distribution at steady state (*V*_ss_) of 0.4–0.8 L/kg has been calculated in healthy volunteers. *C*_max_ values were 50–100 µg/mL, and *C*_min_ values were 25–50 µg/mL under this regimen (see Table 2) [97]. *C*_max_ > 100 µg/mL, and *C*_min_ < 25 µg/mL must be avoided.

**Table 2:** Overview on pharmacokinetics of 5-flucytosine

Standard dose (mg/kg)	25–37.5 mg/kg four times per day
*C*_max_ (µg/mL)	50–100
*V*_d_ (L/kg)	0.4–0.8
Protein binding (%)	3–4
*t*_1/2_ (h)	3–6
Elimination	Glomerular filtration
Renal impairment	Dose reduction guided by glomerular filtration rate
Hepatic impairment	Flucytosine should be avoided because of hepatotoxicity, no effect on pharmacokinetics because of renal elimination
Remark	Therapeutic drug monitoring strongly recommended because of toxicity

Details and references are displayed in the text

*C*_max_ flucytosine peak level; *V*_d_, apparent volume of distribution; *t*_1/2_ half-life

### Flucytosine in special patient groups

The flucytosine plasma clearance resembles the creatinine clearance. Prolonged *t*_1/2_ of up to 85 h has been observed in renal failure [101]. Accordingly, a prolonged dosage interval of 12 h (37.5–50.0 mg/kg b.i.d.) is recommended when creatinine clearance is 20–40 mL/min and of 24 h for a creatinine clearance of 10–20 mL/min (37.5–50.0 mg/kg once daily) [101].

As 5-flucytosine is efficiently eliminated via haemodialysis, it has to be applied after the dialysis sessions [99, 101–103]. Because of its low protein binding and its small molecular weight, an efficient elimination via continuous renal replacement therapy has to be anticipated. The optimal dosage for patients with renal failure requiring continuous veno-venous haemofiltration or continuous veno-venous haemodialysis is not yet established. In an early study on seven patients on continuous arteriovenous or veno-venous haemofiltration, prolonged *t*_1/2_ of 16–37 h were found after a single dose of 2.5 g. There was a linear relationship between ultrafiltration rate (16 mL/min on average) and 5-flucytosine elimination. The volume of distribution amounted to 0.77–0.98 L/kg. The authors recommended administration of 2.5 g with adaption of the dosage interval, e.g. 12 h for an ultrafiltration rate of 20 mL/min [104]. Thomson et al. measured an elevated *C*_max_ of 110 µg/mL after 3 days of treatment with 50 mg/kg per day. The estimated half-life and clearance were 37 h and 1.1 L/h, respectively. Therefore, they suggested a dose of 2.5 g every 48–72 h [105]. Recently, an 81-year-old patient (body weight 97 kg) was treated with 2.5 g of oral 5-flucytosine twice daily during continuous veno-venous haemofiltration using a contemporary protocol with an ultrafiltration rate of 2.5 L/h and a polyarlethersulfone membrane. This resulted in supra-therapeutic *C*_max_ and *C*_min_ were of 120 and 81 μg/mL, respectively, causing thrombocytopenia [106]. Even highly efficient continuous veno-venous haemodiafiltration with a dialysate flow rate of 1 L/h and an ultrafiltration rate of 2 L/h (blood flow rate 200 mL/h) did not normalise 5-flucytosine elimination. Under standard dose (25 mg/kg q.i.d. intravenously), *C*_max_ and *C*_min_ amounted to 120 and 74 µg/mL, respectively, *t*_1/2_ was 12.6 h [107].

For patients with hepatic impairment, no reduction of the flucytosine dose is recommended, as flucytosine does not undergo significant hepatic biotransformation or biliary elimination [98, 103, 108, 109]. But its hepatotoxicity limits its use in this condition.

### Drug–drug interactions involving flucytosine

Pharmacokinetic drug–drug interactions involving the cytochrome P 450 system are a minor concern in flucytosine treatment. Nephrotoxic co-medication such as amphotericin B or cyclosporine A, however, can lead to enhanced flucytosine levels. The toxicity of 5-flucytosine, probably correlates with fluorouracil plasma concentrations. Fluorouracil is degraded by dihydropyrimidine dehydrogenase. Therefore, simultaneous treatment with flucytosine and inhibitors of dihydropyrimidine dehydrogenase such as nucleoside analogues, e.g. brivudin or sorivudine is contra-indicated. After cessation of brivudin or sorivudine flucytosine treatment must not be started within 4 weeks. In addition, pharmacodynamic drug–drug interactions have to be considered. The myelotoxic effects of antineoplastic and immunosuppressive medications are increased when flucytosine is applied. Cytarabine interferes with fungal flucytosine permease thus abolishing its antifungal activity [97]. Although not available in our institutions, we strongly advocate therapeutic drug monitoring of 5-flucytosine, in particular, when it is administered to patients with potentially altered pharmacokinetics.

### Target-site penetration of flucytosine

Flucytosine displays a favourable penetration into various relevant compartments such as human CSF where 71–85% of the simultaneous serum concentrations are achieved. Relatively high levels were also measured in saliva (~50% of the respective serum levels), in ascites (~25–40% of the respective serum concentration), and in bronchial secretion (~76% of the respective serum concentration). Flucytosine kinetics in bronchial secretion was assessed in a dog model displaying almost constant levels of about 20 µg/mL over 3 h [110]. Even at the so-called sanctuary sites, considerable flucytosine concentrations were reached, e.g. 10 µg/mL in aqueous humour (20% of the serum level), 3 µg/mL in bone (30% of the respective serum level), and 26 µg/mL in synovial fluid (41% of the serum concentration). In peritoneal fluid, flucytosine levels were comparable to the simultaneous plasma levels [91]. Highest concentrations are measured in urine (~tenfold serum concentration) [108].

## Antifungal azoles

The azole antifungals can be divided into two subclasses the imidazoles and the triazoles. The imidazoles contain a heterocyclic five-member ring with two nitrogen atoms. The triazole group comprises three nitrogen atoms. Ketoconazole is the only imidazole that can be applied systemically. Fluconazole and itraconazole, as well as the newer broad spectrum antifungals voriconazole, posaconazole and isavuconazole are triazoles. Azole antifungals inhibit the 14-α-demethylase by binding to its haem group. This enzyme is required for conversion of lanosterol into ergosterol. Lack of ergosterol in the fungal cell membrane and accumulation of toxic precursors contribute to the fungistatic activity of azoles. The 14-α-demethylase belongs to the cytochrome P 450 (CYP) family. It is termed as CYP51A1. However, azoles also inhibit other isoenzymes of the CYP system causing thereby numerous drug–drug interactions. Mutations of the 14-α-demethylase (CYP51A1) gene can cause azole resistance [111, 112].

## Ketoconazole

Ketoconazole (e.g. Fungoral^®^, Janssen-Cilag, Beerse, Belgium) is an imidazole for topical and systemic administration. Its antifungal spectrum comprises *Candida* species, *Cryptococcus immitis, Histoplasma capsulatum, Malassezia furfur, Paracoccidioides brasiliensis* and dermatophytes. Nevertheless, ketoconazole lost its role in systemic antifungal therapy.

### Dosage and plasma pharmacokinetics of ketoconazole

An oral dose of 200–400 mg once daily has been applied for the treatment of fungal infections. The oral bioavailability of ketoconazole is highly variable and dependent of oral nutrition and gastric pH. Its plasma protein binding amounts to 84%, 15% are bound to erythrocytes [101]. Ketoconazole is transformed in the liver into inactive metabolites by CYP3A4. Finally, it is eliminated via the bile. Ketoconazole is a strong inhibitor of P-gp and CYP3A4 causing numerous drug–drug interactions. Its elimination half-life (*t*_1/2_β is ~2 h, the terminal half-life (*t*_1/2_γ) amounts to 8 h. A favourable penetration into the urine, the saliva, the synovial fluid, into sebum and cerumen has been described [101].

### Drug–drug interactions involving ketoconazole

Today, ketoconazole is used as a model drug for inhibition of CYP3A4 and P-gp in pharmacokinetic studies. Thus, enhanced plasma concentrations of cyclosporine A, clarithromycin, telithromycin, everolimus, antihistamines, rosiglitazone, midazolam, isavuconazole, riociguat, drospirenone, tetrahydrocannabinol, and cannabidiol were measured during concomitant administration of ketoconazole [113–123].

### Endocrinologic effects and current indication of ketoconazole

Ketoconazole also inhibits corticosteroid synthesis [124]. Today, systemic ketoconazole as a tablet form (Ketoconazole HRA, Laboratoire HRA Pharma, Paris, France) is therefore licensed for medical treatment of endogenous Cushing’s syndrome in adults and adolescents above the age of 12 years. Maintenance doses required for this indication range from 400 to 1200 mg per day taken orally in two to three divided doses to restore normal cortisol levels. As ketoconazole also inhibits testosterone synthesis, it has been used in androgen independent prostate cancer [125].

## Fluconazole

Fluconazole is a triazole comprising a phenyl ring which is substituted by two fluoride atoms in position 2 and 4 and two azole rings. Unlike the other azoles, it displays high solubility in water. Various *Candida* species and *Cryptococcus* species are susceptible to fluconazole [126, 127]. Since *C. albicans* is still the most common species, fluconazole plays also an important role in antifungal prophylaxis. It is available for intravenous and oral administration. In general, fluconazole is well tolerated, but hepatotoxicity and prolongation of the QT interval in ECG resulting in life-threatening ventricular arrhythmias are harmful adverse effects.

### Dosage and plasma pharmacokinetics of fluconazole

The therapeutic dose of fluconazole is guided by the indication. For patients in with invasive candidiasis in stable condition, recent guidelines recommend a loading dose of 12 mg/kg (800 mg) followed by a maintenance dose of 6 mg/kg (400 mg) once daily administered by intravenous infusion [12]. Lower oral doses are sufficient for uncomplicated skin, mucosal or urinary tract infections. After oral administration, fluconazole is well absorbed [bioavailability (*F*) >90%]. Food intake, gastric pH, and gastro-intestinal surgery had no major influence on enteral fluconazole absorption [128–130]. In healthy volunteers, intake of 400 mg of fluconazole led to a *C*_max_ of 9.1 µg/mL. A *C*_max_ of 1.7 µg/mL and an AUC_0−∞_ of 93 µg h/mL were measured after an oral dose of 100 mg. *T*_max_ amounted to 0.5–1.0 h. Fluconazole has a plasma protein binding of ~12%, and a *t*_1/2_ of ~30 h [129]. Thus, it takes 6 days to achieve steady state concentrations unless a loading dose is applied. The total fluconazole CL in healthy volunteers was 15–24 mL/h/kg [114, 130–132] and the apparent volume of distribution at steady state (*V*_ss_) was about 0.75 L/kg [132, 133]. Fluconazole is eliminated via the kidney by 60 to 80% where it undergoes glomerular filtration and tubular re-absorption (see Table 3).

**Table 3:** Overview on pharmacokinetics of fluconazole, voriconazole and isavuconazole

	Fluconazole	Voriconazole	Isavuconazole
Intravenous standard dose	Loading dose 12 mg/kg once Maintenance dose 6 mg/kg once daily	Loading dose 6 mg/kg b.i.d. on day1 Maintenance dose 4 mg/kg b.i.d.	Loading dose 200 mg t.i.d. on day 1 and day 2 Maintenance dose 200 mg once daily
Oral standard dose	Depends on clinical indication	Loading dose 400 mg b.i.d. on day1 Maintenance dose 200 mg b.i.d.	Loading dose 200 mg t.i.d. on day 1 and day 2 Maintenance dose 200 mg once daily
*C*_max_ (µg/mL)	9 after 400 mg i.v.	4.4 after i.v. administration	2.6
AUC (µg h/mL)	93 (AUC_0-∞_ after 400 mg i.v.)	30 (AUC_τ_ after i.v. administration)	34 (AUC_τ_ after i.v. administration)
V_d_ (L/kg)	0,7	4.5	~6.5
Protein binding (%)	12	58	98–99
t_1/2_ (h)	30	~6	80–120
CL (mL/h/kg)	15–24	~100	~30–70
Metabolism and elimination	Mainly unchanged via the kidney, tubular re-absorption	Hepatic metabolism involving 2C9, 2C19, and CYP3A4	Hepatic metabolism involving UGT, and CYP3A4
Renal impairment	Dose reduction (by 50% for GFR 11-50 mL/min)	Standard dose, consider SBECD accumulation during i.v. infusion	Standard dose
Hepatic impairment	No relevant hepatic metabolism, consider hepatotoxicity	Mild to moderate: 50% dose reduction, TDM recommended	Mild to moderate, enhanced levels, no dose reduction recommended by the manufacturer
Remark	Strong inhibitor of CYP3A4 and 2C9, continuous renal replacement therapy requires enhanced dose	Strong inhibitor of CYP2C9 and 2C19, moderate inhibitor of CYP3A4	Inhibitor of CYP3A4, P-gp and BCRP

Details and references are displayed in the text

*C*_max_, peak level; *i.v.* intravenous; *AUC* area under the concentration–time curve; *V*_d_ apparent volume of distribution; *t*_1/2_ half-life; *CL* clearance; *GFR* glomerular filtration rate; *CYP* cytochrome P 450; *SBECD* sulfobutylether-β-cyclodextrin; *TDM* therapeutic drug monitoring; *UGT* uridine diphosphate glucuronosyltransferase, *P-gp* P-glycoprotein; *BCRP* breast cancer related protein

### Drug–drug interactions involving fluconazole

Hepatic metabolism does not play a role in fluconazole elimination. But fluconazole is a strong inhibitor CYP3A4 and CYP2C9. Numerous drug–drug interactions must therefore be considered [113, 129]. Simultaneous treatment with CYP3A4 and CYP2C9 substrates should therefore be avoided, in particular with those prolonging the QT interval [133–135]. Cyclosporine A, tacrolimus or sirolimus are substrates of CYP3A4. Transplant recipients on immunosuppression with one of these drugs are therefore at a high risk of adverse effects, e.g. nephrotoxicity or over-immunosuppression. Dose reduction and close therapeutic drug monitoring of these immunosuppressives is mandatory when the combination with fluconazole is thought to be indispensable [136–144]. Combination of fluconazole with warfarin prolongs the prothrombin time and can cause severe bleedings [145, 146]. Fluconazole inhibits phenytoin metabolism via CYP2C bearing the risk hepatic and neurological adverse effects [147–149]. *C*_max_ and AUC_0−∞_ values of tolbutamide were enhanced by fluconazole causing hypoglycaemia [150]. *C*_max_ of celecoxib, which has affinity to CYP2C9, increased by 60% and the AUC by 130%. A markedly prolonged sedative effect of midazolam and triazolam has to be anticipated in combination with fluconazole [113, 151]. The levels of levonorgestrel and ethinyl estradiol were moderately enhanced by 40 and 24%, respectively, under fluconazole treatment. Although fluconazole is mainly eliminated via the kidney, CYP3A induction by rifampin can decrease fluconazole exposure bearing the risk of treatment failure [152, 153]. The metabolism of rifabutin appears to be inhibited by fluconazole [154].

### Fluconazole in special patient groups

For critically ill patients, doses of 800–1200 mg per day resulting in *C*_max_ values of 40–60 µg/mL have been proposed [155]. This is supported by the observation of an impaired target-site penetration in septic patients [156]. In 15 critically ill patients treated with fluconazole at a median dose of 4.9 (2.3–5.0) mg/kg, concentrations were highly variable and five patients did not reach the pharmacokinetic/pharmacodynamic target defined as a ratio between AUC_0–24 h_ (free drug) and MIC of 100 or greater (*f*AUC_0–24h_/MIC ≥100) [157]. For obese critically ill patients, fluconazole dosage according to the actual body weight (loading dose 12 mg/kg, maintenance dose 6 mg/kg per day) has recently been proposed. This recommendation is based on a pharmacokinetic study of 21 patients. Six patients were obese with a body mass index of 30.0–39.9 kg/m^2^; four patients were morbidly obese with body mass index ≥40 kg/m^2^ [158]. In extremely premature infants with a birth weight <750 g, intravenous or oral administration of 6 mg/kg twice weekly appears to be adequate [159].

For patients with renal failure, a reduction of the fluconazole maintenance dose is necessary because of delayed elimination. A prolonged *t*_1/2_ of 96 h and a 50-percent decrease in fluconazole CL (~10 mL/h/kg) were determined in renal failure with a creatinine clearance of 35 mL/min [132]. Accordingly, the dose of fluconazole should be reduced by 50% in patients with a creatinine clearance of 11–50 mL/min. High amounts of fluconazole are eliminated by renal replacement therapy. Its plasma concentration was decreased by ~40% during a 4-h haemodialysis session [160]. During continuous ambulatory peritoneal dialysis, *t*_½_ was 79 h, and CL was 8 mL/kg/h, which are values comparable to those obtained in patients with a creatinine clearance of 35 mL/min [131, 161]. Continuous renal replacement therapy such as continuous veno-venous hemofiltration and haemodiafiltration is highly efficient in fluconazole elimination. This is explained by the low protein binding, the high water solubility, the relatively small molecular weight of fluconazole, and by the lack of tubular re-absorption in patients with renal failure. During haemodiafiltration a mean *C*_max_ of 26 µg/mL, a very short *t*_1/2_ of only 9 h, and a high CL of 60 mL/h/kg were determined after infusion of 800 mg of fluconazole over 2 h. Based on these data, 500–600 mg twice daily have been suggested for patients on haemodiafiltration [162]. For continuous veno-venous haemofiltration, an intravenous dose of 800 mg once daily has been recommended [163]. Critically ill patients on prolonged intermittent renal replacement therapy appear to require a loading dose of 800 mg of fluconazole followed by 400 mg twice daily (before and after prolonged intermittent renal replacement therapy) for treatment of infections with susceptible *C. albicans* [164]. In a patient on sustained low-efficiency diafiltration, fluconazole kinetics was determined in plasma and subcutaneous interstitial fluid using microdialysis technique. Fluconazole rapidly penetrated into subcutaneous interstitial fluid [165].

During treatment with ECMO, *V*_d_ of fluconazole was enhanced in children. Based on population modelling, the authors suggest treatment with an enhanced loading dose of 35 mg/kg followed by standard maintenance dose [166].

### Target-site penetration of fluconazole

Animal studies on rabbits and rats revealed relatively high fluconazole tissue concentrations [113, 167, 168]. Fluconazole concentrations in urine, in blister fluid, in blister roof, in skin scrapings, in vaginal mucosa, in saliva, in sputum and in CSF were assessed in early clinical studies. The highest fluconazole concentrations were measured in urine and in skin exceeding plasma levels. In most of the tissues, fluconazole concentrations were similar to the simultaneous plasma concentrations, e.g. in CSF, 50–90% of the respective plasma levels [169–171]. In human brain samples obtained from tumour surgery, Thaler et al. measured a mean fluconazole concentration of 17.6 µg/g (133% of the respective plasma level) [172]. Sinnollareddy et al. found a variable target-site penetration of fluconazole in critically ill patients with sepsis. In subcutaneous interstitial fluid, AUC_0–24 h_ was about 50% lower than the AUC_0–24 h_ in plasma [156]. In ascites of a liver transplant recipient treated with fluconazole for 5 days (loading dose 400 mg, maintenance dose 150 mg per day, serum creatinine 1.7 mg/dL), *C*_min_ amounted to 9.6 µg/mL (85% of the simultaneous plasma level). Biliary *C*_min_ values of 9.0 and 6.3 µg/mL (~50% of *C*_min_ in plasma) were measured in two other liver transplant recipients [173]. Biliary concentrations of up to 14 µg/mL were reached by intravenous or oral administration of 200 mg of fluconazole per day (serum creatinine 2.8 mg/dL) [174].

## Itraconazole

Itraconazole (e.g. Sporanox^®^, Janssen-Cilag Ltd, Beerse, Belgium; Itraconazol Universal Farma^®^, Universal Farma, Barcelona) is a triazole with a high lipophilicity. It is active against numerous dermatophytes and yeasts, such as *Candida* and *Cryptococcus neoformans*, and against several *Aspergillus* species [175–177]. Itraconazole was licensed and used for treatment of invasive aspergillosis, because it had been effective in two open-label studies [178, 179]. Today, itraconazole is widely used for local fungal infections. But according to current guidelines, it has also a role in treatment of chronic pulmonary aspergillosis, blastomycosis, histoplasmosis, and coccidioidomycosis [180–184]. Adverse effects comprise gastro-intestinal symptoms, hepatotoxic effects, and congestive heart failure [175].

### Dosage and plasma pharmacokinetics of itraconazole

The oral dose of itraconazole recommended by the manufacturer for systemic mycoses is 200 mg once daily or b.i.d. The intravenous formulation is not widely available. The recommended intravenous dose is 200 mg b.i.d. for the first 2 days followed by 200 mg once daily. Because of potential nephrotoxicity of the solvent vehicle hydroxypropyl-β-cyclodextrin the manufacturer recommends early switch to oral treatment with 200 mg b.i.d., e.g. after 5 days if enteral absorption can be anticipated. Absorption of oral itraconazole amounts to ~55%, but is highly variable and depending on food intake, when the capsule formulation is used. It may be decreased by infections and other co-morbidities [185–190]. Absorption of the oral suspension which contains hydroxypropyl-β-cyclodextrin is better without food intake, and it is superior to that of the capsule formulation [191, 192]. The intravenous itraconazole formulation contains also hydroxypropyl-β-cyclodextrin. At steady state, itraconazole has a *t*_1/2_ of ~30 h. The *T*_max_ is 5 h, the plasma protein binding is as high as 99.8%, *V*_d_ is large (11 L/kg) [36]. Itraconazole undergoes excessive hepatic metabolism involving CYP3A4. The most active metabolite is hydroxy-itraconazole. Fifty-four percent of the administered dose are eliminated via the faeces, 35% via the urine after metabolism (see Table 4). The majority of metabolites are inactive [185, 193, 194]. As itraconazole absorption is highly variable and exposure is difficult to predict, therapeutic drug monitoring will be indispensable for the treatment of systemic infections in the majority of cases [195].

**Table 4:** Overview on pharmacokinetics of itraconazole and posaconazole

	Itraconazole	Posaconazole, oral suspension	Posaconazole, tablet formulation	Posaconazole, intravenous
Standard dose	Loading dose 200 mg b.i.d. Maintenance dose 200 mg once daily—200 mg b.i.d	Therapeutic dose 200 mg q.i.d. or 400 mg b.i.d Prophylaxis 200 mg t.i.d.	Loading dose 300 mg b.i.d. on day 1 Maintenance dose 300 mg once daily	Loading dose 300 mg b.i.d. on day 1 Maintenance dose 300 mg once daily
*C*_max_ (µg/mL)	0.3–1.3	0.6	2	2.6
*V*_d_ (L/kg)	11	20	5	3.7
Protein binding (%)	99.8	98–99	98–99	98–99
*t*_1/2_ (h)	30	29	35	27
CL (mL/h/kg)	Dose-dependent, highly variable	485	130	100
Metabolism and elimination	Excessive metabolisms involving CYP3A4	Metabolisms involving CYP3A4, P-gp substrate		
Renal impairment	No dose reduction, enhanced dose during continuous renal replacement therapy	No dose adjustment	No dose adjustment	Avoid because of SBECD accumulation, When GFR <50 mL/min
Hepatic impairment	Consider dose reduction, TDM	No dose adjustment	No dose adjustment	No dose adjustment
Remark	Variable enteral absorption, strong inhibitor of CYP3A4 causing numerous drug–drug interactions, TDM recommended	Poor, variable enteral absorption, strong inhibitor of CYP3A4 causing numerous drug–drug interactions	Strong inhibitor of CYP3A4 causing numerous drug–drug interactions	Strong inhibitor of CYP3A4 causing numerous drug–drug interactions

Details and references are displayed in the text

*C*_max_ peak level; *AUC* area under the concentration–time curve; *V*_d_, apparent volume of distribution; *t*_1/2_ half-life; *CL* clearance; *CYP* cytochrome P 450; *SBECD* sulfobutylether-β-cyclodextrin; *GFR* glomerular filtration rate; *TDM* therapeutic drug monitoring

### Drug–drug interactions involving itraconazole

As itraconazole is a strong inhibitor of CYP3A4, a long list of drug–drug interactions has to be considered. Co-administration of itraconazole with lovastatin, atorvastatin, simvastatin, or quinidine is contra-indicated. Plasma levels of many other CYP3A4 substrates such as midazolam, triazolam, cyclosporine A, tacrolimus, sirolimus and everolimus, methylprednisolone, warfarin, digoxin, carbamazepine, rifabutin, and anti-retroviral drugs such as ritonavir, indinavir, and saquinavir will increase when itraconazole treatment is started in patients on treatment with one of these drugs [113, 196]. Co-administration of negative inotropic drugs may enhance the risk of congestive heart failure, and plasma levels of calcium antagonists are enhanced by itraconazole [175, 197–199]. In vitro, itraconazole has also a strong inhibitory potential on the ATP-binding cassette transporters P-gp, breast cancer related peptide (BCRP), and bile salt export pump (BSEP, ATP-binding cassette protein B11 or ABCB11). P-gp is also inhibited by the metabolite hydroxy-itraconazole [200].

### Itraconazole in special patient groups

For critically ill patients, intravenous infusion for 7 days, followed by oral administration has been proposed [201]. Itraconazole is not eliminated by intermittent hemodialysis. Surprisingly, continuous haemodiafiltration resulted in increased itraconazole elimination. Doses exceeding 300 mg t.i.d. may be required [191, 202].

### Target-site penetration of itraconazole

In the skin and in fat, the highest itraconazole concentrations were measured exceeding the simultaneous plasma concentrations 19-fold and 17-fold, respectively. In liver, in lung, particularly in alveolar macrophages, in kidney, spleen, bone, and in muscle, concentrations were above the plasma levels [191, 203].

## Voriconazole

Although voriconazole has a chemical structure which is similar to that of fluconazole, its antifungal spectrum is much broader. *Aspergillus* species, *Candida* species, *Scedosporium, Fusarium* and some endemic fungi are susceptible to voriconazole. *Zygomycetes*, however, are resistant. Because it had achieved better clinical outcome than amphotericin B deoxycholate in an open-label randomized trial of invasive aspergillosis, it is recommended as first-line drug for this disease [10, 204, 205].

Voriconazole displays a short post-antifungal effect (PAFE) [206]. Depending on the fungal strain and on the method applied, an AUC/MIC >32–100 showed the best correlation with antifungal effectiveness [112, 207].

Voriconazole is available as a tablet formulation (50 mg of film-coated tablets, 200 mg of film-coated tablet, Vfend^®^, Pfizer Limited, Sandwich, Kent, UK; Voriconazole Accord, Accord Healthcare Limited, North Harrow, Middlesex, UK), a 40-mg/mL of oral solution (Vfend^®^, Pfizer Limited, Sandwich, Kent, UK) and an intravenous formulation comprising sulfobutylether-β-cyclodextrin sodium (SBECD) as a solubiliser (Vfend^®^, Pfizer Limited, Sandwich, Kent, UK; Voriconazole Hospira^®^, Hospira UK Limited, Royal Leamington Spa, Warwickshire, UK).

### Dosage and plasma pharmacokinetics of voriconazole

The recommended intravenous standard dose is 6 mg/kg b.i.d. on day 1 (loading dose) followed by 4 mg/kg b.i.d. (maintenance dose). The oral dose for adult patients is 400 mg b.i.d. on day 1 followed by 200 mg b.i.d. For adult patients with a body weight of less than 40 kg, a dose reduction by 50% is recommended (loading dose, 200 mg b.i.d maintenance dose, 100 mg b.i.d.). In healthy volunteers, the bioavailability of voriconazole amounts to 96% and is independent from gastric pH, but may be considerably lower in patients. Voriconazole displays non-linear pharmacokinetics. After treatment with oral standard dose, a *C*_max_ of 2 µg/mL and a *C*_min_ of 0.5 µg/mL were measured on day 7. An increase of the dose by a factor of 1.7 led to a 2.4-fold elevation of *C*_max_ and a 3.1-fold increase of the AUC over the dosage interval (AUC_*τ*_) [36, 208, 209]. *C*_max_ is usually reached 1.5–3 h after oral intake. After administration of the recommended intravenous dose, *C*_max_ and AUC_*τ*_ amounted to 4.4 µg/mL and 29.5 µg h/mL, respectively, in male healthy volunteers [210]. The plasma protein binding of voriconazole amounts to 58% which is markedly lower than that of all the other azoles but fluconazole. The *V*_d_ of voriconazole is ~4.5 L/kg, and CL ~7 L/h (~100 mL/h/kg) in healthy volunteers. Voriconazole undergoes hepatic phase I biotransformation involving CYP2C9, CYP2C19, and CYP3A4. Rate-limiting is the fluoropyrimidine-*N*-oxidation. As there is a genetic polymorphism of CYP2C9, there are ultra-rapid and poor voriconazole metabolizers. A fourfold elevation in plasma levels has been found in the latter population. In Asians, a prevalence of slow voriconazole metabolizers of ~20% has been reported [208, 209]. Inactive metabolites of voriconazole are eliminated by ~80% via the urine and by ~20% via the faeces (see Table 3) [209, 210]. On average, *t*_1/2_ of voriconazole is ~6 h at standard dosage, but it increases with the plasma concentration.

### Drug–drug interactions involving voriconazole

Voriconazole is a strong inhibitor of CYP2C19, CYP2C9 and a moderate inhibitor of CYP3A4. It is also a substrate for CYP2C19, CYP2C9 and CYP3A4. Therefore, numerous potentially dangerous drug–drug interactions have to be anticipated during treatment with voriconazole. Inhibition of the metabolism of immune-suppressants causing enhanced exposure can be particularly harmful. Therefore, the doses of cyclosporine A and of tacrolimus have to be reduced by 50 and 66%, respectively, when voriconazole treatment is initiated. Close therapeutic drug monitoring of immune-suppressants is indispensable to avoid excessive immunosuppression and renal damage. Voriconazole has been shown to enhance *C*_max_ and AUC_*τ*_ of sirolimus by 556 and 1014%, respectively. Concomitant use of sirolimus and voriconazole is therefore contra-indicated [211]. Enhanced plasma concentrations of vitamin K antagonists and probably of direct acting oral anticoagulants by voriconazole bear the risk of severe haemorrhage. The sedative effect of benzodiazepines is prolonged by voriconazole. This is also true for the combination with the opioids fentanyl, alfentanil, oxycodone and methadone. In patients on sulfonylureas, voriconazole treatment may cause hypoglycaemia. Statin levels can be enhanced by voriconazole bearing the risk of rhabdomyolysis. We therefore recommend discontinuation of statin therapy as long as voriconazole is administered. For omeprazole, a dose reduction by 50% is advised when voriconazole is concomitantly used. Accumulation of histamine blockers (e.g. terfenadine and astemizole), cimetidine or quinidine under voriconazole treatment is particularly dangerous, because of an additional pharmacodynamic drug–drug interaction. These drugs, just as voriconazole, may cause prolongation of the QT interval resulting in torsades de pointes [212]. Impaired efficacy of voriconazole because of sub-therapeutic plasma levels is caused by co-administration of CYP inducers such as rifampicin or carbamazepine. An enhanced voriconazole dosage of 5 mg/kg i.v. or 350 mg p.o. b.i.d. has been recommended, when the combination of voriconazole and rifabutin appears to be indispensable [211]. Combination with the HIV protease inhibitors saquinavir, amprenavir and nelfinavir may enhance exposure to these drugs and to voriconazole [211]. The non-nucleoside HIV reverse transcriptase inhibitors delavirdine and efavirenz also cause elevated voriconazole levels [208, 211, 213]. Intake of St. John ´s wort will lower voriconazole levels [214].

### Voriconazole in special patient groups

For children (2–11 years old) and for young adolescents with low body weight (12–14 years old, body weight <50 kg), the manufacturers recommend a loading dose of 9 mg/kg every 12 h on day 1, followed by an intravenous maintenance dose of 8 mg/kg twice daily. Oral administration of a maintenance dose 9 mg/kg twice daily (maximum dose of 350 mg b.i.d.) can be considered, but a 50-percent decrease in voriconazole exposure has to be anticipated with the oral regimen. In lung transplant recipients, the absorption of voriconazole is significantly decreased with a bioavailability of only 24–63% [215]. In patients with mild or moderate liver cirrhosis (stage Child–Pugh A and B) voriconazole metabolism is impaired and CL is delayed by ~50%. Therefore, the maintenance dose should be reduced by 50% [208]. Severe liver disease (e.g. liver cirrhosis Child–Pugh stage C), may result in prolongation of *t*_½_ by ~tenfold [216, 217]. Drug monitoring is essential in this condition.

Renal impairment at any stage appears to have no relevant influence on voriconazole pharmacokinetics, and does not require dose adjustment for the oral voriconazole preparations [208]. However, a considerable accumulation of the solvent vehicle SBECD was observed in patients with impaired renal function undergoing intravenous voriconazole treatment [208]. SBECD the solubiliser of the intravenous voriconazole preparation is a large cyclic oligosaccharide which is potentially nephrotoxic at higher concentrations. Therefore, the manufacturer advises to prefer oral voriconazole in patients with a creatinine CL <50 mL/min.

In a patient on continuous veno-venous haemodiafiltration, voriconazole pharmacokinetics was reported to be similar to that in patients off haemodiafiltration. The extracorporeal CL by haemodiafiltration was <10% of the total CL [218]. In nine critically ill patients undergoing continuous veno-venous haemodiafiltration, mean *C*_max_ and *C*_min_ were 5.9 and 1.1 mg/L, respectively, after a single intravenous 6-mg dose. A mean AUC_0–12 h_ of 22.4 µg h/mL, a *V*_d_ of 228 L, a *t*_1/2_ of 14.7 h, a sieving coefficient of 0.56, and mean total CL of 12.9 L/h were reported. The extracorporeal CL via continuous veno-venous haemodiafiltration was ~1 L/h. Despite an enlarged *V*_d_, a prolonged *t*_½_ and an increased total CL, the voriconazole exposure was similar to that in healthy subjects, and no dose adjustment was recommended for patients on continuous veno-venous haemodiafiltration [217]. From six patients on continuous veno-venous hemofiltration, a similar *C*_max_ value (mean 4.3 µg/mL) and a somewhat higher voriconazole exposure (mean AUC_0–12 h_, 53.5 µg h/mL) were reported. The mean sieving coefficient was lower (0.22), and *t*_1/2_ was longer (27.6 h). Because of a considerable variability in plasma levels, the authors recommend therapeutic drug monitoring [219]. In a more recent study of ten critically ill patients on continuous veno-venous haemofiltration, the latter findings were confirmed: mean *C*_max_ was 4.1 µg/mL, *C*_min_ 2.4 mg/L, *t*_1/2_ 19.5 h, and the mean AUC_0–12 h_ was 37 µg h/mL [220]. In a single patient on high-volume continuous veno-venous hemofiltration, a somewhat higher voriconazole exposure was observed despite an efficient extracorporeal CL of voriconazole (AUC_0–12 h_, 65 mg h/L, total CL, 5.4 L/h, *t*_1/2_, 16.5 h, *V*_d_, 128.6 L, sieving coefficient, 0.58, extracorporeal CL, 1.4 L/h). This was ascribed to impaired hepatic metabolism because of multi-organ failure [221].

The effects of different renal replacement techniques on SBECD kinetics have also been investigated. Not surprisingly, continuous renal replacement therapy was more efficient in SBECD elimination than intermittent. As long as information on safety of SBECD is insufficient, alternatives for intravenous voriconazole should be considered in patients with renal impairment. Oral administration with therapeutic drug monitoring could be an option in stable conditions, intradialytic administration in terminal renal failure. In patients on continuous renal replacement therapy, administration of the intravenous voriconazole preparation appears to be safe [220, 222, 223].

During veno-arterial ECMO, voriconazole levels were low or even undetectable, whereas therapeutic concentrations where achieved by an enhanced dose in a patient on veno-venous ECMO [64, 224].

Given the complex pharmacokinetics and metabolism, the variable absorption and the numerous drug–drug interactions, therapeutic drug monitoring has a paramount role in voriconazole treatment to warrant sufficient dosage and therapeutic safety [195, 225].

### Target-site penetration and pharmacokinetics of voriconazole

Voriconazole displayed high tissue penetration in animal models [208, 226]. In human autopsy samples, median voriconazole concentrations amounted to 3.41 µg/g in the brain, 6.26 µg/g in the lung, 6.89 µg/g in the liver, 5.60 µg/g in the spleen, 6.47 µg/g in the kidneys, and 7.55 µg/g in myocardium [227]. In CSF, variable voriconazole concentrations have been measured. In samples obtained by lumbar puncture, the median concentration amounted to 0.65 µg/mL [penetration ratio, 0.46 (range 0.22–1.00)] [226]. When CSF was taken from ventricular drainage, lower levels of 0.08–0.17 µg/mL were found [228]. Voriconazole kinetics in pulmonary ELF were assessed in 20 healthy volunteers who had received the intravenous standard dose for 3 days. Sampling of ELF and blood was performed 4, 8, 12, or 24 h after start of voriconazole administration (n was 5 for each time point, *T*_inf_, 2 h). In plasma, mean *C*_max_, *t*_1/2_, and AUC_*τ*_ were 5.3 mg/mL, 6.9 h, and 39.5 µg h/mL, respectively. In ELF, mean *C*_max_ amounted to 48.3 µg/mL, and in alveolar macrophages 20.6 µg/mL. The AUC_τ_ values calculated for ELF and alveolar macrophages were 282 and 178 µg h/mL, respectively. The penetration ratio for ELF (expressed by AUC_τ_ in ELF/AUC_*τ*_ in plasma) amounted to 7.1. For alveolar macrophages it was 4.5 (AUC_*τ*_ in alveolar macrophages/AUC_*τ*_ in plasma). [229]. In a study of 12 lung transplant recipients on voriconazole, single ELF and plasma samples were obtained at different times after oral intake. Concentrations in ELF amounted to 0.29–83.32 µg/mL. The estimated *T*_max_ in ELF was ~6 h. The penetration ratio (C in ELF/C in plasma) was 11 + 8 (mean + standard deviation) in this study population [230]. Voriconazole could also be recovered from pleural empyema. The concentrations amounted to 0.8–1.4 µg/mL, the penetration ratio (C in empyema/C in plasma) 0.45–0.95 [231].

## Posaconazole

Posaconazole (Noxafil^®^, Merck Sharp & Dohme Ltd, Hoddesdon, Hertfordshire, UK) is a triazole with a wide antimycotic spectrum that includes *Mucorales*. Its chemical structure resembles that of itraconazole. Depending on the animal model applied AUC/MIC ratios >400 and >1000, respectively, have been correlated with optimal antifungal efficacy [232, 233]. Posaconazole is licensed for antifungal prophylaxis in selected haematological high risk patients, i.e. allogeneic stem cell transplant recipients with graft versus host disease and patients with acute myeloid leukaemia or myelodysplastic syndrome. The prophylactic indication is based on two randomized controlled trials. In patients who had undergone allogeneic haematopoietic stem cell transplantation and suffered from graft versus host disease, the rate of proven or probable invasive aspergillosis was lower under posaconazole than under fluconazole prophylaxis (2.3 vs. 7.0%, *P* = 0.006). However, the primary endpoint of this study, reduction of all invasive fungal infections, was missed [234]. A significantly lower incidence of invasive fungal infections, in particular invasive aspergillosis, was achieved by posaconazole in comparison with fluconazole or itraconazole prophylaxis in patients with acute myeloid leukaemia or myelodysplastic syndrome undergoing aggressive remission-inducing chemotherapy [235]. In an open-label, multicentre study of 107 patients on posaconazole salvage therapy for invasive aspergillosis and other mycoses, a response rate of 42% was achieved. In the retrospective control group (86 patients), the response rate was only 26% [236]. Therefore, posaconazole is licensed for second-line treatment of invasive aspergillosis. Further licensed indications comprise second-line treatment of fusariosis, chromoblastomycosis, coccidioidomycosis, and mycetoma.

### Dosage and plasma pharmacokinetics of posaconazole

For a decade, posaconazole had been available only as an oral suspension displaying poor and highly variable absorption [237–241]. For this suspension, splitting of the therapeutic dose of 800 mg/d resulted in enhanced posaconazole exposure, because of saturable enteral absorption [237, 242]. Therefore, a therapeutic dose of 200 mg q.i.d and a prophylactic dose of 200 mg t.i.d. had been recommended for this formulation [237, 242]. Intake of fatty nutrition or nutritional supplements is necessary to warrant adequate absorption with this preparation [237–239]. This was a particular challenge in patients undergoing myeloablative chemotherapy or in stem cell transplant recipients suffering from graft versus host disease. After a single dose of 400 mg, *t*_1/2_ was ~20 h, *C*_max_ was 0.6 µg/mL, *T*_max_ 6.3 h, and the AUC_0−∞_ was 19.4 µg h/mL [2, 239] (see Table 4).

Recently, an intravenous formulation and a tablet formulation with improved bioavailability have been launched. The gastro-resistant 100-mg-tablets comprise hypromellose acetate succinate and croscarmellose sodium. In a phase I study, oral intake of 100 mg of posaconazole in different tablet and capsule formulations including the currently available tablet form led to *C*_max_, AUC_0–168 h_, and AUC_0–∞_ values that were more than three times above the respective values achieved by the same dose of the oral posaconazole suspension (median *C*_max_, ~0.35 versus 0.08 µg/mL, median AUC_0–∞_, 11 versus 3 µg h/mL). Notably, the median *V*_d_ was 1450 L after administration of the suspension and only 340 L after tablet intake. A slightly shorter *t*_1/2_ was found in the tablet groups (~25 versus ~29 h), whereas CL was much slower for the tablet form (~9 versus ~34 L/h) [243]. *C*_max_, AUC_0–∞_ and AUC_0–last_ achieved by the tablet form were largely uninfluenced by co-medication affecting gastric pH and motility [244]. Doses of 200 and 400 mg were compared in a further phase I study after a single administration and at steady state [245]. On day 14, the mean *C*_max_ amounted to 1.8 and 2.9 µg/mL, and AUC_0–24h_ values were 31 and 57 µg h/mL after tablet intake at daily doses of 200 and 400 mg, respectively. Similar results were obtained from 50 patients with haematological malignancies, where the median *C*_max_ amounted to 2.1 µg/mL, and the median CL was 9.4 L/h [246]. The plasma protein binding of posaconazole amounts to 98–99% [239] (see Table 4). Intake of the tablets together with a fat-rich meal enhanced the AUC_0–72 h_ by 50% [247].

The concentrate for solution (300 mg per vial) for infusion contains Betadex sulfobutylether sodium (SBECD) as a solubiliser. The infusion should be applied via a central venous line to avoid thrombophlebitis. *T*_inf_ should be 90 min. Pharmacokinetics of the intravenous posaconazole formulation have been assessed in patients on myeloablative chemotherapy for haematologic malignancies [248]. After a single intravenous 300-mg dose, mean *C*_max_ and AUC_0–24 h_ amounted to 1.6 µg/mL and 8.2 µg h/mL, respectively. On day 14 of treatment with 300 mg once daily (loading dose 300 mg b.i.d.), a median *C*_max_ of 2.6 µg/mL, an AUC_0–24 h_ of 34 µg h/mL, a *C*_min_ of 1.1 µg/mL, and an accumulation ratio of 2.8 were determined. The recommended standard dose for the tablet and the intravenous formulation is 300 mg b.i.d. on day 1 followed by 300 mg once daily (see Table 4).

Posaconazole is glucuronidated in the liver by UDP-glucuronyl-transferase (UGT) 1A4. Inactive mono- and diglucuronides are formed. After intake of radiolabelled posaconazole as an oral suspension, 77% of the administered dose was recovered from the faeces, where the unchanged parent drug accounted for 66%. Only 14% of the applied radioactivity was detected in the urine, almost exclusively as glucuronides [2].

### Drug–drug interactions involving posaconazole

CYP isoenzymes have no relevant role in posaconazole disposition. However, posaconazole is a strong inhibitor of CYP3A4 causing numerous drug–drug interactions, e.g. increased levels of tacrolimus (2.2-fold *C*_max_, and 4.5-fold AUC), of cyclosporine A, of glipizide, and of midazolam [237, 249]. Recently, it has been demonstrated that CYP3A4 inhibition is more pronounced when the tablet form is applied. For sirolimus, administration of 60% of the standard dose every third day has been proposed when it is combined with posaconazole [250]. The manufacturer advises against concomitant treatment with sirolimus and posaconazole, and recommends dose reduction for cyclosporin A, and for tacrolimus as well as close drug monitoring. Levels of simvastatin, atorvastatin, ergot alkaloids, vinca alkaloids, HIV protease inhibitors, midazolam, and verapamil are also enhanced by posaconazole. Recently, posaconazole has been reported to be a strong inhibitor of P-gp and BCRP in vitro [200].

Posaconazole is also a substrate for P-gp. Therefore, its plasma concentrations are enhanced by concomitant use of P-gp inhibitors such as verapamil, cyclosporine A, quinidine, clarithromycin, and erythromycin. Rifabutin, efavirenz, fosamprenavir, and phenytoin decrease posaconazole levels [249, 251, 252].

Therapeutic drug monitoring has been mandatory in patients on treatment or prophylaxis with the oral posaconazole suspension to warrant therapeutic levels [253, 254]. Target trough concentrations ≥ 1.0 and 0.7 µg/mL have been suggested for treatment and prophylaxis, respectively [255]. Although exposure is probably less variable with the new formulations, therapeutic drug monitoring is still advocated [256].

### Posaconazole in special patient populations

In a study of critically ill patients, the majority presented sub-therapeutic posaconazole plasma levels during treatment with standard doses of the oral suspension [257]. Mild to moderate renal or hepatic impairment had no relevant influence on posaconazole pharmacokinetics. In elderly (≥65 years), the AUC was enhanced by 29–42%. Thus, the manufacturer recommends standard dose for the latter conditions. Posaconazole is not removed by haemodialysis and in patients with terminal renal failure, posaconazole exposure on and off haemodialysis was almost identical [241, 251]. However, the manufacturer advises against the intravenous formulation in patients with moderate or severe renal impairment because of accumulation of the intravenous vehicle SBECD. There is one case report on the use of intravenous posaconazole during continuous veno-venous haemofiltration. A *C*_max_ of 2.8 µg/mL and a *C*_min_ of 1.7 µg/mL were measured. Thus, concentrations were comparable with those reported from patients off haemofiltration. Although SBECD exposure was 2.5 times higher than that in healthy volunteers, the authors do not anticipate relevant toxic effects [258].

### Target-site penetration and kinetics of posaconazole

Pulmonary target-site pharmacokinetics of posaconazole was investigated in two clinical studies on alveolar ELF. Posaconazole was administered as an oral suspension at a dose of 400 mg b.i.d. In the first study, 25 healthy adults were enrolled. Comparable posaconazole concentrations were measured in ELF and in the plasma (mean *C*_max_ 1.9 and 2.1 µg/mL, respectively). Posaconazole accumulated in alveolar cells (mean *C*_max_ 87.7 µg/mL) [259]. In a second study of 20 lung transplant recipients by the same authors group, posaconazole concentration were slightly lower: *C*_max_ was 1.3 µg/mL in ELF, 1.3 µg/mL in plasma, and 55.4 µg/mL in alveolar cells [260]. During the perioperative period, highly variable levels were measured in bronchoalveolar lavage fluid of transplant recipients on prophylaxis with posaconazole oral suspension [261].

In autopsy samples obtained from stem cell transplant recipients who had been on treatment with low doses, the highest posaconazole tissue levels have been recovered from the liver (up to 7.5 µg/g), followed by kidney (up to 4.6 µg/g), lung (up to 4.6 µg/g), myocardium (up to 1.8 µg/g), and brain (maximum 0.3 µg/g) [258, 262]. Lung concentrations exceeding the respective blood levels were recently determined in a rat model [263].

Posaconazole concentrations achieved in CSF were low (1.2–4.6 ng/mL) or even undetectable [264–266]. In brain abscess fluid and CSF, respectively, of two patients with severe encephalitis and obvious disturbance of the blood brain barrier, concentrations of ~0.2 µg/mL could be achieved by administration of the oral suspension [266]. In muscle of a burn patient on continuous haemodialysis treated with standard doses of the oral suspension, posaconazole levels were below 0.1 µg/mL, probably because of poor absorption [267]. In vitro, fluorophore-labelled posaconazole accumulated in host and fungal cell membranes [268, 269].

## Isavuconazole

The active antifungal drug isavuconazole is cleaved by butyrylcholinesterase and other plasma esterases from its water soluble prodrug isavuconazonium sulphate (Cresemba^®^, BAL 8557, Basilea, Basel, Switzerland). The chemical structure of isavuconazole is similar to that of fluconazole and voriconazole [270]. Its antifungal spectrum comprises *Candida*, including non-albicans, *Aspergillus* species, and *Mucorales* such as *Mucor, Rhizopus, Rhizomucor,* and *Cunninghamella*. Isavuconazole is inactive against *Fusarium* and *Sporothrix schenckii* [271]. In a randomized controlled double blind trial of 516 patients, it was as effective as voriconazole for treatment of invasive aspergillosis and other mould infections [272]. Hepatotoxicity, gastro-intestinal and central nervous adverse effects may occur during isavuconazole treatment. Whereas prolongation of the QT interval is a common adverse effect of azole antifungals, shortening of the QT interval is observed under isavuconazole. The clinical impact of the latter observation is not yet clear [273].

### Dosage and plasma pharmacokinetics of isavuconazole

Isavuconazonium sulphate is available for intravenous and for oral administration. There is a powder for concentrate for solution for infusion (200 mg isavuconazole as 372.6 mg isavuconazonium sulphate per vial), and there are hard capsules containing 100 mg of isavuconazole, equivalent to 186 mg of isavuconazonium sulphate. Because of the water solubility of isavuconazonium sulphate, no solvent is required for the intravenous formulation. As the oral bioavailability amounts to 98%, the intravenous and the oral dose are identical. Treatment has to be started with a loading dose of 200 mg three times per day applied for 2 days. The maintenance dose amounts to 200 mg once daily given as a 1-h infusion (one vial diluted to 0.8 mg/mL) or orally (two capsules) with or without food. Isavuconazole has a high plasma protein binding of 98–99%. Its volume of distribution amounts to 300–500 L, *T*_max_ is about 2 h after oral intake [274]. In male healthy volunteers, mean *C*_max_ values at steady state were 2.61 and 2.55 µg/mL after oral and intravenous administration, respectively. A single 200-mg loading dose followed by 100 mg once daily over 14 days had been applied. The mean AUC_0–24 h_ amounted to 41.5 and 33.6 mg h/L, respectively [274]. After single oral doses of 100–400 mg and intravenous doses of 50–200 mg, there was a linear increase of AUC with the dose [275]. Isavuconazole undergoes hepatic metabolism involving CYP3A4, CYP3A5, and subsequently UGT. Urinary excretion of unchanged isavuconazole is minimal with 0.02–0.04 and 0.06–0.38% of the administered dose after oral intake and after infusion, respectively. Equal amounts of the metabolites are excreted via urine and faeces. Isavuconazole has a long *t*_1/2_ of about 80–120 h. The systemic CL was between 1.9 and 5.0 L/h in healthy volunteers [274, 275]. A population pharmacokinetic analysis was performed using data from nine phase 1 studies and one phase III study. The mean isavuconazole CL was 2.36 L/h (see Table 3). In Asians, it was 36% lower than in Caucasians [276]. This observation, however, was not confirmed in patients suffering from infections with filamentous fungi [277]. The mean AUC_0–24 h_ amounted to 92 µg h/mL in healthy volunteers, and to 101 µg h/mL in patients with IFI. Pharmacokinetic/pharmacodynamic modelling showed that the treatment with isavuconazole at standard doses is effective against *Aspergillus* strains with MIC values ≤0.5 µg/mL according Clinical and Laboratory Standards Institute (CLSI) methodology and ≤1 µg/mL according European Committee on Antimicrobial Susceptibility Testing (EUCAST) methodology [276].

### Drug–drug interactions involving isavuconazole

As isavuconazole is a moderate CYP3A4 inhibitor, numerous drug–drug interactions have to be considered. Enhanced plasma levels of cyclosporine A, tacrolimus, sirolimus, and mycophenolate mofetil have to be anticipated, when isavuconazole is co-administered. The AUC_0–∞_ of tacrolimus was enhance by 125% that of sirolimus by 84%, and that of cyclosporine A and mycophenolic acid by 29 and 35%, respectively [278]. Dose reduction and close therapeutic drug monitoring is strongly advised whenever isavuconazole is applied together with immunosuppressants. This is also true for the combination with digoxin. The pharmacokinetic effects of isavuconazole on colchicine and dabigatran appear to be less pronounced, but a dose reduction may be required. If the combination with midazolam or atorvastatin is indispensable, close monitoring is necessary [279]. Recently, isavuconazole did not have a clinically significant effect on warfarin pharmacokinetics in a phase I study of 20 healthy males [280]. An increase of isavuconazole levels has to be considered under treatment with lopinavir and ritonavir. Concomitant therapy with rifampin, carbamazepine, barbiturates or St John’s wort is contra-indicated because of resulting sub-therapeutic isavuconazole levels. A decrease in plasma levels of bupropion, lopinavir and ritonavir by isavuconazole has been reported [279]. Along with CYP3A4/5-mediated drug–drug interactions, effects on drug transporters might play a role. A recent in vitro study revealed considerable inhibition of the ATP-binding cassette transporters P-gp and BCRP by isavuconazole [200].

### Isavuconazole in special patient groups

In a phase III study (“SECURE study”), moderate to severe renal dysfunction (calculated creatinine clearance <50 mL/min) was an exclusion criterion [272]. In patients suffering from infections with various moulds, dimorphic fungi, and non-*Candida* yeasts, population pharmacokinetics isavuconazole has been analysed. The estimated glomerular filtration rate did not correlate with the isavuconazole CL [277]. Mild, moderate and severe renal impairment did not influence isavuconazole pharmacokinetics. No relevant extracorporeal isavuconazole CL took place during intermittent haemodialysis and isavuconazole exposure remained largely unchanged. The investigators conclude that probably no dose adjustment is required for patients with end-stage renal disease undergoing intermittent haemodialysis [281]. The manufacturer recommends standard dosage for patients suffering from renal failure including those with end-stage renal disease. The effect of continuous renal replacement therapy on isavuconazole elimination, however, has not yet been assessed.

The impact of impaired liver function on isavuconazole pharmacokinetics has been assessed in patients with mild (Child–Pugh Class A) or moderate (Child–Pugh Class B) alcoholic liver cirrhosis who had received a single oral or intravenous dose of 100 mg. There was a significant increase in isavuconazole exposure in patients with hepatic dysfunction compared to healthy volunteers. After intravenous infusion of 100 mg of isavuconazole, the mean values for AUC_0–∞_ were 0.039, 0.072, and 0.101 µg h/mL for normal, mildly impaired, and moderately impaired liver function, respectively. Half-lives amounted to 123, 224 and 302 h, respectively, and CL values were 2.13, 1.93 and 1.43 L/h for normal, mildly and moderately impaired liver function, respectively. Very similar values were obtained after oral isavuconazole intake [282]. A recently published population pharmacokinetic analysis revealed a mean isavuconazole CL of 1.55 L/h in patients with mild or moderate liver impairment, and a CL of 2.5 L/h in healthy volunteers. Simulations showed mean trough concentrations at steady state of 3.5, 5.3, and 6.1 µg/mL for normal, mildly impaired and moderately impaired liver function, respectively. Thus, there was a less than twofold increase in trough concentrations for subjects with mild and moderate hepatic impairment in comparison with healthy subjects [283]. Based on these data, the manufacturer recommends standard dose for patients with mild or moderate liver dysfunction. Pharmacokinetic data from patients with severe hepatic impairment (Child–Pugh Class C) are lacking.

Impaired absorption requiring an increased dose has been observed in a patient after Roux-en-Y gastric bypass surgery [284]. No pharmacokinetic data is available for patients younger than 18 years.

### Isavuconazole target-site concentrations

Until now, there is only one report on penetration of isavuconazole into human tissue. Isavuconazole levels in three soft tissue biopsies (muscle and fat) taken from a patient with mucormycosis 3 h after dosing were 1.09, 1.27 and 1.38 µg/g. The corresponding plasma level was 0.85 µg/mL [285]. Data from animal studies suggest a favourable tissue penetration. In a murine meningitis model, cerebral isavuconazole concentrations exceeded simultaneous plasma levels [286].

## Echinocandins

Echinocandins are cyclic hexa-lipopeptides linked with an *N*-aryl side chain which is relevant for their antifungal activity. Echinocandins act by non-competitive inhibition of β-(1, 3)-d-glucan synthase which is localized in the fungal cell membrane. The polysaccharide β-(1, 3)-d-glucan is an essential component of the inner layer of the fungal cell wall, which plays an important role for cellular integrity [287–289]. Depletion of β-(1,3)-d-glucan results in characteristic morphological changes such as thinning of cellular wall, abnormal swelling, and an irregular shape of the fungal cell, and in aberrant budding [290]. Echinocandins are fungicidal to *Candida* including several non-albicans strains, e.g. *C. glabrata*, *C. krusei*, and *C. lusitaniae* and fungistatic to *Aspergilli. Cryptococcus neoformans, Fusarium species,* and *Zygomycetes* are resistant to echinocandins. The fungistatic effect of echinocandins on *Aspergilli* can be assessed by morphological evaluation. The activity of echinocandins is quantified by the minimal effective concentration (MEC). A paradoxical pharmacodynamic effect of echinocandins has been observed in vitro and in vivo. When a susceptible fungus is exposed to an increasing echinocandin concentration, after the first sub-inhibitory phase, an inhibition of fungal growth is achieved (second phase). If the echinocandin concentration will be further increased, a decline of the antifungal activity takes place (third phase). Finally, at highest concentrations (fourth phase), fungal growth is inhibited again [291–293]. The underlying mechanism and the clinical impact of this paradoxical pharmacodynamic effect are not yet clear [291]. Obviously, it may occur at therapeutic concentrations. Synthesis of cell wall chitin, as well as protein kinase C, and calcineurin have been speculated to be involved [292]. Echinocandins display a relevant post-antifungal effect and therefore a concentration-dependent activity [294, 295]. The ratio *C*_max_/MIC as well as AUC/MIC are looked upon as relevant pharmacokinetic/pharmacodynamic indices [294, 296–299].

The echinocandins are recommended for the treatment of moderately and severely compromised patients with invasive candidiasis by current guidelines [300–302]. They are relatively well tolerated and display a low risk of drug–drug interactions. Adverse effects of echinocandins comprise headache, nausea, diarrhoea, phlebitis and pruritus, but also severe adverse reactions such as leukopenia, neutropenia, anaemia, hypokalaemia and hepatotoxicity [303–305]. The latter is a particular concern in micafungin treatment. Micafungin has therefore a restricted indication in the EU. All therapeutically used echinocandins, display poor enteral absorption and are therefore only available for intravenous infusion [306].

## Caspofungin

Caspofungin (Cancidas^®^, Merck & Co., Inc. Whitehouse Station, N.J., USA) is produced by chemical modification of a fermentation product obtained from *Glarea lozoyensis.* Its molecular weight amounts to 1093 Da. Caspofungin has its major role in the treatment of invasive candidiasis [12, 13, 307, 308]. It is also recommended for empirical antifungal therapy in neutropenic patients with fever that persists under broad spectrum antibacterial treatment [309]. Based on an open non-comparative trial of 83 patients who had failed to respond to standard therapy or did not tolerate this treatment, caspofungin is licensed for salvage therapy of invasive aspergillosis. The standard treatment had been performed with conventional or lipid-formulated amphotericin B, itraconazole or voriconazole. A response was achieved in 45%, a complete response in only 5%, a partial response in 40% [310]. Caspofungin is not licensed for first-line treatment of invasive aspergillosis as two studies were discouraging. In 61 patients, there was a progression of aspergillosis in 51%, a complete response in only 2%, and partial response in 31%, and stable disease in 15% of the patients [311]. Of 24 patients after hematopoietic stem cell transplantation suffering from proven or probable invasive aspergillosis, 42% had complete or partial response, and 50% had a progressive disease [307, 312].

### Dosage and plasma pharmacokinetics of caspofungin

The standard dose of caspofungin is 70 mg as a single loading dose, followed by a maintenance dose of 50 mg once daily, or 70 mg once daily, when the body weight exceeds 80 kg. In healthy volunteers, a poly-exponential elimination with a *t*_½_β of 8–10 h and a *t*_½_γ of 27 h has been described (see Table 5). Caspofungin displays linear pharmacokinetics [313, 314]. A mean *C*_max_ of 12 µg/mL and a mean AUC_0–∞_ of 118 µg h/mL were determined after a 70-mg single dose (*T*_inf_ = 1 h) [313]. Similar values were measured after a loading dose of 70 mg followed by a maintenance dose of 50 mg once daily for 14 days. No relevant accumulation took place, when this regimen was applied. AUC_0–24h_ was 97.6 and 100.5 µg h/mL on day 1 and on day 14, respectively. With daily doses of 50 and 70 mg, moderate accumulation of ~50% was observed within 2 weeks [314]. The caspofungin CL is about 10 mL/h/kg. Immediately after administration, caspofungin undergoes a rapid distribution into tissue, mainly into the liver. When ^3^H caspofungin had been infused 40% of the administered dose was found in the urine and 34% in the faeces [313, 314]. Caspofungin is bound to plasma proteins by 95% [313, 314]. A change in *V*_d_ with values of ~0.05 L/kg at the start of therapy and an increase to 0.3–2.0 L/kg within the first days of treatment has been reported [313]. In a phase II study of patients with proven or probable invasive aspergillosis, high-dose treatment with daily doses of 70, 100, 150, and 200 mg was assessed. Caspofungin displayed linear pharmacokinetics over the entire range. Body weight was found to be a significant covariate for CL. Patients with hepatic impairment, however, had been excluded from this study. After infusion of 200 mg of caspofungin, *C*_max_ and *C*_min_ amounted to 40.6 and 11.8 µg/mL, respectively, and the AUC was 500 µg h/mL [315, 316].

**Table 5:** Overview on pharmacokinetics of echinocandins

	Caspofungin	Anidulafungin	Micafungin
Dose, mg once daily	Loading dose 70, maintenance dose 50 (70 if body weight >80 kg)	Loading dose 200 (*T*_inf_, 180 min), maintenance dose 100 (*T*_inf_, 90 min)	50 for prophylaxis, 100 for candidaemia, 150 for oesophageal candidiasis
*C*_max_ (µg/mL)	10	7	18 (dose 150 mg)
Volume of distribution (L/kg)	0.3–2.0	0.6	0.3
Protein binding (%)	92.4–96.5	99.0	99.9
*t*_1/2_ (h)	8	40–50	13–20
CL (mL/h/kg)	~10	15	~12
Metabolism and elimination	Independent from cytochrome P-450 (CYP)	Spontaneous degradation in plasma	CYP involved
Renal impairment	No dose adjustment	No dose adjustment	No dose adjustment
Hepatic impairment	Enhanced exposure in moderate hepatic impairment, dose reduction	Slightly lowered concentrations, no dose adjustment recommended	Slightly lowered concentrations, contra-indicated in European SmPC
Remark	Dose reduction in critically ill patients with liver dysfunction may cause underexposure		Potential risk for liver tumours

Details and references are displayed in the text

*C*_max_ peak level; *AUC* area under the concentration–time curve; *t*_1/2_ half-life; *CL* clearance; *V*_d_ apparent volume of distribution; *T*_inf_ infusion time

Caspofungin is transformed in the liver. Isoenzymes of CYP obviously have no relevant role in caspofungin metabolism. Caspofungin is hydrolysed to M0, its main metabolite which emerges in the plasma 24–30 h after infusion. Metabolite M1 is also formed by hydrolysis, and is *N*-acetylated forming M2. The metabolites M0, M1 and M2 are eliminated via the urine [317].

### Drug–drug interactions involving caspofungin

Caspofungin has no relevant influence on activity of CYP enzymes. In vitro, however, it was found to interfere with ATP-binding cassette transporters. Obviously it is a weak P-gp inhibitor, but a strong inhibitor of BCRP [200]. In clinical practice, drug–drug interactions are not a major problem in caspofungin treatment. When cyclosporine A was co-administered, caspofungin exposure was elevated by 35%. In contrast, tacrolimus caused slightly lowered *C*_max_ values of caspofungin. Caspofungin concentrations were also lowered by simultaneous administration of efavirenz, nevirapine, rifampicin, dexamethasone, phenytoin or carbamazepine [318].

### Caspofungin in special patient groups

In allogenic haematopoietic stem cell transplant recipients, mean *C*_max_ (8.5 µg/mL) was similar to, and mean *C*_min_ (2.9 µg/mL) was slightly above the values reported from other study populations on standard dosage [319].

For patients with moderate hepatic impairment, reduction of the maintenance dose to 35 mg/d is advised. This recommendation is based on data obtained from patients with mild liver cirrhosis (Child–Pugh Score 5–6) or moderate liver cirrhosis (Child–Pugh Score 7–9) who were in an otherwise stable condition. The patients were matched to healthy subjects. Patients with moderate liver cirrhosis were treated with a reduced maintenance dose of 35 mg once daily, patients with mild cirrhosis with the standard dose. A slight elevation in caspofungin concentrations observed in mild hepatic insufficiency was judged as clinically irrelevant. In patients with moderately impaired liver function, the reduced dose led to caspofungin concentrations comparable with those in the control group [320]. In critical illness, patients with moderate liver dysfunction may achieve sub-therapeutic caspofungin exposure when the dose is adjusted (AUC_0–24 h_ was 65 instead of ~100 µg h/mL). This was recently found out by Martial and colleagues applying pharmacokinetic/pharmacodynamic modelling and Monte Carlo simulation. The authors ascribe the low concentrations to typical pathophysiological alterations occurring in critically ill patients, e.g. hypoalbuminaemia, and advise standard dose for this population [321]. In a critically ill male patient (body weight 85 kg with) suffering from liver cirrhosis (Child–Pugh Score 9), therapeutic caspofungin exposure has been achieved with administration of the standard dose [322].

In 38 critically ill patients treated with standard doses of caspofungin at a surgical ICU, *C*_min_ values were significantly higher when the body weight was <75 kg and the serum albumin level >23.6 g/L [323]. Recently, van der Elst et al. reported a median AUC_0–24 h_ of 78 µg h/mL from 20 patients treated at intensive care units (ICU) with standard doses. Thus, caspofungin exposure was somewhat lower than in healthy subjects and may result in sub-optimal efficacy. Based on their data and on population pharmacokinetic modelling the authors suggest a dose of 1 mg/kg bodyweight for critically ill patients [324]. Variable levels and an even lower exposure (mean AUC_0–24 h_, 52 µg h/mL, mean *C*_max_, 3.9 µg/mL) has been found by Sinnollareddy et al. in seven critically ill patients [157]. In contrast, pharmacokinetic parameters obtained from 21 ICU patients on caspofungin at standard doses were similar to those of non-critically ill patients. On day 7 of caspofungin treatment (*n* = 13), median AUC_0–24 h_ was 107.2 (90.4–125.3) µg h/mL, *C*_min_ 2.55 (1.82–3.08) µg/mL, *C*_max_ 8.65 (7.16–9.34) µg/mL, *V*_d_ 7.03 (5.51–7.73) L and CL 0.54 (0.44–0.60) L/h [median (interquartile range)] [325].

Since caspofungin elimination is largely independent from renal function, standard dosage is suggested in patients with renal impairment, even in those with terminal renal failure requiring haemodialysis (see Table 5) [318, 326]. Two studies have investigated the influence of continuous renal replacement therapy on caspofungin kinetics. In critically ill patients on continuous veno-venous haemofiltration or on continuous veno-venous haemodialysis, pharmacokinetic parameters were comparable with those in critically ill patients not on renal replacement therapy or in healthy volunteers. The extracorporeal caspofungin CL was negligible. Thus, standard dosage has therefore been advised for patients undergoing continuous renal replacement therapy [327]. More recently, Roger et al. performed a pharmacokinetic study in critically patients on vasopressor support undergoing veno-venous haemofiltration (*n* = 5) or veno-venous haemodiafiltration (*n* = 7). In addition, they performed population pharmacokinetic modelling and Monte Carlo simulations. Based on their results, they recommend an enhanced loading dose of 100 mg followed by standard maintenance dose [328].

Caspofungin plasma levels have also been reported from two critically ill patients on ECMO. In a young female on veno-arterial ECMO, most of the caspofungin levels were below the lower limit of detection [64]. In contrast, a male on veno-venous ECMO presented therapeutic *C*_min_ and *C*_max_ values [224].

### Target-site concentrations of caspofungin

In rats, caspofungin has reached relatively high tissue concentrations in the liver and the kidneys, intermediate concentrations in spleen, lung (mean 2.4 µg/g), red blood cells, and small intestine. In heart, lymph nodes, muscle, eyes and brain (mean 0.2 µg/g) caspofungin concentrations were low. The rats had been treated with a high a single dose of 2.0 mg/kg [H^3^] labelled caspofungin. In the liver, 22.2 µg/g (35% of the dose) on average was measured 24 h after injection. Lower concentrations were detected in lung and brain. [313]. In a patient with cholangitis, biliary caspofungin levels were measured after infusion of 70 mg using a bioassay. Levels amounted to 0.8 µg/mL at 1 h, to 1.0 µg/mL at 2 h, and to 0.6 µg/mL at 3 h after infusion. The serum concentration at 1 h was 3.1 µg/mL [329].

## Anidulafungin

Anidulafungin (Ecalta^®^, Pfizer Limited, Sandwich, Kent, UK) has a molecular weight of 1140 Da and contains three phenyl groups in its side chain. It is licensed for the treatment of invasive candidiasis in adult patients.

### Dosage and plasma pharmacokinetics of anidulafungin

The recommend standard dose is 200 mg once on day 1 (loading dose) and 100 mg once daily on subsequent days (maintenance dose). The infusion rate should not exceed 1.1 mg/min. Therefore, a *T*_inf_ of 3 h is required for the loading dose and a *T*_inf_ of 90 min for the maintenance dose. For antifungal prophylaxis in immunocompromised patients, administration of 200 mg every 48 h and 300 mg every 72 h was compared with the standard regimen and resulted in similar AUC_0–144 h_ values [330].

Plasma pharmacokinetics of anidulafungin has been studied in healthy volunteers and in various patient populations [331]. A population pharmacokinetic analysis was performed on 600 plasma levels obtained from 225 subjects during phase II and phase III trials. A mean *t*_1/2_ of 25.6 h, a *V*_ss_ of 32.5 L (0.54 L/kg) and a CL of 0.93 L/h (15 mL/h/kg) has been reported. A mean *C*_max_ of about 7 µg/mL has been measured after 100 mg per day; the AUC_0–∞_ was 106 µg h/mL [246]. In nine healthy volunteers, who had obtained 90 mg of ^14^C-labeled anidulafungin, the mean *C*_max_ was 4.11 µg/mL, the AUC was 102.2 µg h/mL and the *t*_½_ amounted to 28 h. Anidulafungin undergoes spontaneous ring opening. The respective product which is further degraded by hydrolysis and *N*-acetylation independently from phase I and II metabolism is eliminated via biliary excretion [303, 332]. The degradants of anidulafungin are eliminated mainly by the faeces. Only 10% of the administered radioactivity was recovered as intact drug, 90% as degradants (see Table 5).

### Drug–drug interactions involving anidulafungin

Obviously, anidulafungin does not inhibit CYP isoenzymes. However, a strong in vitro inhibition of the ATP-binding cassette transporter BCRP by anidulafungin has been demonstrated [200]. Several clinical studies addressed eventual drug–drug interactions with anidulafungin, e.g. co-medication with voriconazole did not cause an interaction [333]. Simultaneous treatment with cyclosporine A (1.25 mg/kg orally applied) and anidulafungin resulted in a 22% increase in the AUC_0–∞_ of anidulafungin. In vitro, anidulafungin exposure had no effect on the metabolism of cyclosporine A [334]. There was no relevant interaction between tacrolimus and anidulafungin in healthy volunteers [335].

### Anidulafungin in special patient groups

Renal impairment has no influence on anidulafungin elimination [303]. Surprisingly, hepatic impairment results in a decreased anidulafungin exposure. An increased degradation due to a reduced protein binding and an enlarged *V*_d_ have been suggested as possible explanations [336]. In morbidly obese subjects, anidulafungin exposure was lowered by one-third on average. The authors conclude that an enhanced dose should be considered for this population. [337]. Based on pharmacokinetic/pharmacodynamic analyses of data from phase II and phase III studies, standard dosage has been recommend for patients with a body weight of up to 150 kg [338, 339]. In critically ill patients, Brüggemann et al. reported a median AUC_0–24 h_ of 83 µg h/mL, a *C*_max_ of 5.9 µg/mL, a *C*_min_ of 2.8 µg/mL, a *t*_½_ of 27 h, and a median *V*_d_ of 40 L at day 7 of standard treatment. Thus, the exposure was slightly below that in healthy subjects or in patients in a more stable condition [340].

Renal replacement therapy by continuous veno-venous haemodiafiltration or continuous veno-venous haemofiltration did not affect anidulafungin pharmacokinetics, and there was no relevant extracorporeal CL [341–343]. In a patients with liver failure treated with albumin dialysis, anidulafungin exposure was in the normal range although *t*_½_ was only 18 h [344]. A patient treated with veno-venous ECMO for acute respiratory distress syndrome presented unchanged anidulafungin kinetics [345].

### Target-site penetration of anidulafungin

Anidulafungin tissue concentrations were assessed in rabbits after 7 days of treatment. After a bolus injection of 5 mg/kg, the highest concentrations were measured in lung (mean 17.9 µg/g) and liver (mean 16.8 µg/g) and the lowest concentrations were found in the brain (mean 1.6 µg/g), in the vitreous humour, the aqueous humour, and in the choroid. On day 7, the mean peak concentration in plasma reached 14.2 µg/mL [346]. Comparable concentrations were measured in rats with tissue half-lives of ~30 h. Tissue concentrations exceeded the simultaneous plasma levels ~tenfold [347]. High anidulafungin concentrations (mean 103.1 µg/mL) were measured in human pulmonary alveolar macrophages obtained from healthy volunteers after 3 days of standard treatment. Mean concentrations in pulmonary ELF and in plasma were lower (0.9 and 1.5 µg/mL, respectively) [229]. In blood, anidulafungin accumulates in peripheral blood mononuclear cells and in polymorphonuclear leukocytes [348].

## Micafungin

The molecular weight of micafungin (Mycamine^®^, Fungard^®^, Astellas, Tokyo, Japan) amounts to 1292 Da and its side chain contains three aromatic rings. Its indications in Europe comprise treatment of invasive candidiasis and prophylaxis of *Candida* infection in patients undergoing allogeneic haematopoietic stem cell transplantation or patients who are expected to have neutropenia as well as treatment of oesophageal candidiasis in adults. After administration of micafungin to healthy volunteers, abnormal liver function tests have been noted. In rats, foci of altered hepatocytes (FAH) and hepatocellular tumours emerged after 3 months micafungin exposure. Therefore, the European Medicines Agency (EMA) restricted the indication of micafungin as follows: “The decision to use Mycamine should take into account a potential risk for the development of liver tumours. Mycamine should therefore only be used if other antifungals are not appropriate.” [304]. The occurrence of FAH or liver tumours during echinocandin treatment in humans has not yet been systematically investigated.

### Dosage and plasma pharmacokinetics of micafungin

Adults and children with a body weight >40 kg should receive 100 mg once daily for the treatment of invasive candidiasis, 150 mg for the treatment of oesophageal candidiasis (3 mg/kg/day for body weight ≤40 kg), and 50 mg once daily for *Candida* prophylaxis. In adult patients undergoing bone morrow or peripheral stem cell transplantation, on additional fluconazole prophylaxis, pharmacokinetics of micafungin was studied. On day 7 of treatment with a daily dose of 100 mg (*T*_inf_ = 1 h), the mean *C*_max_ was 22.0 µg/mL, the AUC_0–24 h_ amounted to 101.6 µg h/mL, *t*_½_ was 12 h, CL was 1.1 L/h, and *V*_ss_ was 17.3 L. [349]. Micafungin has a very high protein binding of 99.85%. Micafungin is metabolised into largely inactive metabolites, i.e. a catechol form (M-1), a methoxy form of M-1 (M-2) and to a further metabolite M-5 formed by hydroxylation at the side chain. In vitro, micafungin is a substrate for CYP3A, but hydroxylation by CYP3A plays a minor role in vivo. The metabolites are excreted mainly via the faeces [304].

Micafungin appears to be effective against most *Candida* species when the AUC/MIC ratio exceeds 3000. Pharmacokinetic/pharmacodynamic targets for various body fluids remain to be established [350, 351]. In patients with *Candida* oesophagitis, administration of 300 mg every other day and 150 mg daily resulted in almost identical mean AUC_0–48 h_ values (311 versus 310 µg h/mL). There was a non-significant trend to better response in patients on intermittent high-dose treatment [295].

### Drug–drug interactions involving micafungin

Micafungin is a weak inhibitor of CYP3A [352]. In vitro, it is a strong inhibitor of multidrug resistance protein 4 (MRP4) and a mild inhibitor of the transporters P-gp, multidrug resistance protein 1 (MRP1), multidrug resistance protein 5 (MRP5), and BCRP [200]. Accordingly, a 15%-reduction in CL of cyclosporine A was observed, when micafungin was concomitantly administered [353]. In contrast, micafungin had no influence on tacrolimus exposure in healthy volunteers [354]. The combination of fluconazole and micafungin had no effect on the pharmacokinetics of fluconazole or micafungin [355]. Induction of CYP3A4 by rifampicin or ritonavir did not exert an influence on the AUC_0–∞_ of micafungin. Warfarin, diazepam, salicylic acid or methotrexate did not affect micafungin concentrations [356].

In patients with febrile neutropenia who had undergone recent allogenic haematopoietic stem cell transplantation, a micafungin dose escalation from 150 to 300 mg per day had no significant influence on cyclosporine A exposure as expressed by the ratios *C*_max_/dose and *C*_min_/dose [357]. Simultaneous administration of high-dose micafungin and low-dose amphotericin B deoxycholate in healthy males resulted in a 30% increase in amphotericin B concentrations but left micafungin levels unchanged [358].

### Micafungin in special patient groups

Micafungin pharmacokinetics was investigated in children aged 2–12 years and in adolescents aged 13–17 years suffering from febrile neutropenia. Children were treated with 0.5–4.0 mg/kg/day, adolescents with 0.5–1.5 mg/kg/day. Administration of 2 mg/kg resulted in a *C*_max_ of 21.4 ± 9.7 µg/mL, an AUC_0–∞_ of 132.3 ± 27.1 µg h/mL, on day 4 (mean ± standard error of the mean). CL was ~20 mL/h/kg, *t*_1/2_, 12–13 h, and *V*_ss_ was 0.3–0.4 L/kg. Mean CL was faster in 2- to 8-year-old than in 9- to 17-year-old children [359]. A mean *C*_max_ of 2.5 µg/mL and an AUC of 20.6 µg h/mL were measured in premature infants after a single micafungin dose of 0.75 mg/kg; *t*_1/2_ was 7.5 h [355]. In children with invasive candidiasis younger than 5 years old, the micafungin CL was faster and the exposure was lower than in those who were at the age of 5 years or over. A similar *t*_1/2_ was found in both groups [360]. Recently, Hope and colleagues performed a population pharmacokinetic analysis on pharmacokinetic data of micafungin and its metabolites M1 and M5 obtained from 229 children between the ages of 4 month and 17 years enrolled in phase I and phase III trials. An AUC_0–24 h_ of 75–139 µg h/mL was set as a target. The authors propose a dose of 1 mg/kg for antifungal prophylaxis, 2 mg/kg for the treatment of invasive candidiasis, and a micafungin dose of 3 mg/kg for the treatment of *Candida* oesophagitis [361].

A study on liver homogenates from neonates and adults suggests that the faster micafungin CL observed in neonates is a result of a higher unbound micafungin fraction in neonatal serum in comparison with adults (mean 0.033 versus 0.004). The expression levels of various transporter proteins, i.e. sodium/taurocholate co-transporting polypeptide (NTCP), organic anion-transporting polypeptides 1B1/3 (OATP1B1/3), BSEP, BCRP and multidrug resistance-associated protein 3 (MRP3 or ABCC3), were similar in neonates and in adults [362].

As mentioned above, the use of micafungin in patients with hepatic impairment is discouraged by the European product information, because of its hepatotoxicity. Like for anidulafungin, the micafungin exposure was significantly reduced in patients with moderately impaired liver function (Child–Pugh score 7–9) in comparison with healthy volunteers (mean AUC_0–∞_, 97.5 versus 125.9 µg h/mL, *P* = 0.03). However, this difference was ascribed to different body weights in both groups. Therefore, no dose adjustment is recommended in moderate hepatic impairment [355, 363]. A lower micafungin exposure and an increased CL were also found in a single-dose study of 8 patients with severe hepatic dysfunction and of 8 healthy subjects. Mean values were 7.3 versus 10.3 μg/mL for *C*_max_, 100 versus 142 μg h/mL for AUC_0–24 h_, and 0.7 versus 1.1 L/h for the micafungin CL in subjects with severely impaired and normal liver function, respectively [364]. No correlation between the degree of liver dysfunction and micafungin levels could be identified in a study of 8 patients with pre-existing hepatic impairment. Due to liver toxicity, however, micafungin treatment had to be stopped in one of the patients [365]. By contrast, *C*_min_ and *C*_max_ of micafungin were significantly enhanced in patients with liver failure caused by graft versus host disease after haematopoietic stem cell transplantation. When serum bilirubin concentration was >5 mg/dL and/or serum γ-glutamyltransferase level was >500 IU/L, median *C*_min_ was 10.5 and *C*_max_ was 27.6 µg/mL. In patients with lower bilirubin and γ-glutamyltransferase, the values amounted to 4.8 and 15.8 µg/mL, respectively [366]. Thus, the effect of impaired liver function on micafungin pharmacokinetics appears to be variably and hardly predictable.

Micafungin pharmacokinetics is unchanged in renal impairment [363, 366]. Three clinical studies addressed the effect of continuous renal replacement therapy on micafungin elimination. Neither continuous veno-venous haemodialysis nor continuous veno-venous haemodiafiltration nor continuous veno-venous haemofiltration had a clinically relevant influence on micafungin CL. Thus, no dose adjustment is required for patients undergoing continuous renal replacement therapy [367–369]. By contrast, an 8-h plasma exchange has shortened *t*_1/2_ of micafungin from 16.5 to 6.3 h and increased CL from 0.37 to 0.93 L/h. Administration after plasma exchange and an increment in dose have therefore been suggested [370].

In 20 critically ill patients treated with 100 mg of micafungin per day, micafungin exposure was somewhat lower than that in healthy volunteers and patients in a more stable condition. The median AUC_0–24 h_ was 78.6 µg h/mL, median *C*_max_ was 7.2 µg/L, *C*_min_ was 1.55 µg/L, *V*_d_ was 25.6 L, CL was 1.3 L/h, and *t*_1/2_ amounted to 13.7 h on average [371]. Thus, exposure was lower than that reported from stem cell transplant recipients [349]. Jullien et al. have performed a population pharmacokinetic analysis of 100 critically ill patients on mechanical ventilation suffering from severe sepsis. The mean micafungin CL amounted to 1.34 L/h. Monte Carlo simulation was applied for the assessment of the probability of target attainment. By the standard dose of 100 mg once daily, a sufficient exposure was achieved in ≥90% of the patients for infections with *C. albicans* or *C. glabrata* with an MIC <0.015. A higher dose is suggested, for infections with *C. parapsilosis* or with less susceptible *C. albicans* or *C. glabrata* strains [372].

In a study of 12 infants on ECMO, Autmizguine et al. observed a slightly increased *V*_d_ and a micafungin CL in the upper normal range. The authors propose a prophylactic dose of 2.5 mg/kg and a therapeutic dose of 5 mg/kg once daily for this population [373]. Micafungin exposure was high (C_max_, 17.4, C_min_, 5.5 µg/mL, AUC_0–24 h_, 207.3 µg h/mL, t_1/2_, 20 h, CL, 0.3 L/h, dose, 100 mg once daily) in a malnourished critically ill patient on extracorporeal carbon dioxide removal and continuous haemofiltration. An increased micafungin elimination by extracorporeal carbon dioxide removal is therefore unlikely [374].

In HIV-infected patients with confirmed oesophageal candidiasis, micafungin pharmacokinetics was similar to that in healthy volunteers [375].

### Target-site penetration and kinetics of micafungin

Micafungin tissue concentrations were measured in rats and rabbits after administration of 1 mg/kg. In rats, the highest micafungin concentrations were found in lung (mean 5.95 µg/mL) and kidney (mean 3.78 µg/mL) followed by liver (mean 2.65 µg/mL) [376]. In rabbits, tissue levels were comparable, and concentrations in brain (mean 0.10 µg/g), choroid (mean 0.061 µg/mL), and vitreous humour (mean 0.015 µg/mL) were very low [377].

Micafungin concentrations achieved in alveolar macrophages, ELF, and plasma of healthy volunteers amounted to a mean of 14.6, 0.52 and 14.8 µg/mL, respectively, after administration of 150 mg daily for 3 days [378]. A similar micafungin distribution has been found in adult lung transplant recipients after the same treatment. Mean *C*_max_ in plasma, ELF, and alveolar cells were 4.93, 1.38, and 17.41 µg/mL, respectively. For susceptible *A. fumigatus* with an MIC of 0.0156 µg/mL, AUC_0–24 h_/MIC ratios of 5077, 923, and 13340 were calculated for plasma, ELF, and alveolar cells, respectively [379].

In pancreatic pseudocyst fluid, a micafungin concentration of 0.38 µg/mL was measured 24 h after administration [380]. Yamada et al. assessed the distribution of micafungin in CSF, pleural effusion, ascites, and wound secretion of seven patients with IFIs. After daily doses of 100–300 mg of micafungin, highest concentrations were measured in wound secretion (4.4 µg/mL), and surprisingly, in CSF (1.9 µg/mL). Micafungin concentrations in pleural effusion (0.7 µg/mL) and in ascites (1.0 µg/mL) were lower [381]. In peritoneal fluid of critically ill post-surgical patients with peritonitis, a median *C*_max_ of 1.2 µg/mL was measured [351]. In bile of a patient suffering from *Candida* cholangitis, the micafungin concentration was 1.9 µg/mL 24 h after infusion of 150 mg [382].

In a burn patient on 200 mg of micafungin once daily, *C*_min_ levels in eschar were 4.0 and 14.8 µg/mL after a single dose and after repeated doses, respectively [383, 384]. Later on, micafungin concentrations in burn eschar and in plasma have been assessed in three other patients. By administration of 200 mg once daily, *C*_min_ levels in eschar of 1.4 and 6.7 µg/mL were achieved after the first dose and at steady state, respectively [383, 384]. Population pharmacokinetics of micafungin was analysed in burn eschar and plasma of 15 patients with severe burn injuries. Daily doses of 100–150 mg had been applied. The mean concentration in burn eschar amounted to 0.7 µg/mL and was below the detection limit in 1 patient. The probability of target attainment was estimated based on target AUC_0–24 h_/MIC ratios of 285 and 3000 for *C. parapsilosis* and *C. non*-*parapsilosis*, respectively. By a single dose of 100 mg, targets were achieved for strains with low MICs of ≤0.008 and ≤0.064, respectively [385].

Intraocular penetration of 150–300 mg of intravenous micafungin per day was studied after vitrectomy in 7 patients. The mean micafungin concentrations were 21.02 µg/mL in the plasma, 0.10 µg/mL in the vitreous humour, and 0.08 µg/mL in the aqueous humour. Lower levels of 0.043 and 0.026 µg/mL had previously been measured in a single patient. Thus, micafungin penetration into aqueous and vitreous humour appears to be poor. Micafungin levels of 1.60–5.99 µg/g in the cornea, 14.65 µg/g in the iris, 1.20 µg/g in the retina, and 5.81 µg/g in the choroid were measured in material from single cases [386, 387].

## Implications of antifungal pharmacokinetics for clinical practice

### Implications for treatment of systemic candidiasis

Candidaemia is the most common manifestation of systemic candidiasis. Recent epidemiological studies revealed a rate of ~1.5 per 1000 hospital admissions and an overall 30-day mortality of 35%. *C. albicans* has been isolated in ~50% of the cases [14, 388]. Echinocandins are first-line drugs for the treatment of candidaemia [12, 13, 308]. Their elimination is independent from renal function. Impaired liver function can lead to increased caspofungin levels, but decreased exposure to anidulafungin and variable alterations of micafungin concentrations. Fluconazole can be used in stable patients with candidaemia at low risk of a resistant pathogen and for step-down therapy. It displays favourable tissue distribution and safety. Renal impairment requires dose reduction. As a strong inhibitor of CYP3A4 and CYP2C9, it causes various drug–drug interactions. Liposomal amphotericin B is an alternative to echinocandins and is indicated for treatment of central nervous and cardiovascular candidiasis. Although safer than the conventional amphotericin B deoxycholate, a considerable nephrotoxicity of liposomal amphotericin B has to be anticipated. Whereas high-dose echinocandins might be an alternative for treatment of *Candida* endocarditis, their use in CNS infections is discouraged because of insufficient target-site penetration. Amphotericin B deoxycholate is recommended for disseminated candidiasis in neonates [12]. In adults, it should be avoided because of its nephrotoxicity and infusion-related adverse effects. Continuous infusion will reduce amphotericin B toxicity but eventually also its efficacy. Only fluconazole, conventional amphotericin B deoxycholate, and flucytosine reach therapeutic urinary concentrations. Echinocandins and amphotericin B lipid formulations are therefore not useful for treatment of urinary *Candida* infections. When 5-flucytosine is applied, its dose has to be adjusted to renal function and close monitoring of drug levels, as well as hepatotoxic and myelotoxic effects is strongly recommended.

### Implications for treatment of invasive aspergillosis

Voriconazole is the drug of choice for the treatment of invasive aspergillosis [10]. It penetrates well into relevant target compartments. However, its complex, non-linear pharmacokinetics requires therapeutic drug monitoring. Voriconazole is involved in numerous drug–drug interactions, e.g. with immunosuppressives, sedatives, anticoagulants, and lipid lowering drugs. CNS and liver are the major targets of voriconazole toxicity. Liposomal amphotericin B is a therapeutic alternative, particularly when azole resistance is a concern. Posaconazole and isavuconazole are options for second-line treatment, but their role is not yet established. Data on their tissue penetration are incomplete.

### Implications for treatment of cryptococcosis

Cryptococcosis is an indication for combined antifungal therapy with amphotericin B and 5-flucytosine [389]. As renal deterioration is common under amphotericin B treatment, even with the liposomal formulation, close monitoring of renal function and of flucytosine levels, if available, is mandatory. Flucytosine easily penetrates into relevant tissues including the CNS. Myelo- and hepatotoxicity limit its used.

### Implications for treatment of mucormycosis

The drug of choice for mucormycosis is liposomal amphotericin B at a dose of 5 mg/kg. For CNS manifestation, even 10 mg/kg are required. The current European guidelines recommend posaconazole as an option for the second-line or savage therapy, although this is off-label [11]. If posaconazole is selected, the recently introduced intravenous formulation should be used in order to warrant therapeutic exposure.

### Implications for antifungal treatment in critical illness

In critically ill patients, characteristic pathophysiological changes such as altered hydration, haemodynamics, tissue perfusion, and plasma protein levels can reduce exposure to antifungals. In general, this effect is more pronounced for hydrophilic drugs. Lower levels of liposomal amphotericin B and in some studies of echinocandins have been reported for critically ill patients. Sufficient dosage, in particular adequate loading doses, is therefore pivotal. The initial dosage must be guided by *V*_d_. Eventually impaired elimination has to be considered during further treatment. Therapeutic drug monitoring should be used in this population.

### Implications for antifungal treatment of patients with impaired renal or liver function

Flucytosine and fluconazole are mainly eliminated via the kidneys. Dose reduction guided by glomerular filtrations rate is therefore required in patients with renal impairment for these drugs (see Tables 2 and 3). Amphotericin B deoxycholate is contra-indicated in patients with potentially reversible renal impairment. No dose adjustment is recommended for liposomal amphotericin B in patients with impaired renal function. However, also this formulation displays a considerable nephrotoxicity. Echinocandins and the broad spectrum azoles voriconazole, posaconazole, and isavuconazole can be given at standard dose in renal failure. Accumulation of the solvent SBECD must be considered for the intravenous forms of voriconazole and posaconazole. Hepatic impairment affects pharmacokinetics of echinocandins. A dose reduction has been recommended for caspofungin when liver dysfunction is moderate. However, underdosage is a concern, particularly in critically ill patients. Anidulafungin can be applied at standard dose, although its levels may be slightly reduced in patients with impaired liver function. Micafungin should be avoided in liver disease. The maintenance dose of voriconazole should be reduced by 50% when it is applied in patients with liver cirrhosis Child–Pugh A and B. We recommend therapeutic drug monitoring in this case. For posaconazole and isavuconazole, no dose adjustment is recommended in patients with liver dysfunction. Drug monitoring and clinical monitoring for toxic effects, however, should be performed.

### Implications for antifungal treatment of patients on extracorporeal circuits

For intermittent haemodialysis, the dose of 5-flucytosine should be reduced to 37.5 mg/kg once after haemodialysis. Fluconazole should be applied at a maintenance dose of 100–200 mg after haemodialysis. For 5-flucytosine treatment during continuous renal replacement therapy, doses between 2.5 g every 72 h and 2.5 g twice daily have been proposed. As there are recent reports on overdosage and severe toxicity, we strongly advise therapeutic drug monitoring to avoid harmful adverse effects in this situation. Fluconazole has to be applied at enhanced maintenance doses of 800 and 1200 mg per day during continuous haemofiltration and haemodiafiltration, respectively. Enhanced doses during continuous renal replacement therapy are also required for itraconazole. Standard doses are appropriate during continuous renal replacement therapy for lipid-formulated amphotericin B, for echinocandins, for voriconazole, and for posaconazole. In patients on intermittent haemodialysis, the SBECD containing intravenous formulations should be avoided, if possible. Antifungal pharmacokinetics during ECMO has been assessed in a few cases rendering conflicting data for caspofungin and voriconazole.

## Combination antifungal therapy

In view of the aforementioned pharmacokinetic properties of antifungals, it is justified to apply combination antifungal therapy (CAF) to maximise the antifungal effect by attacking the same or different targets in fungal cells and making use of the synergistic effect [390].

Combination therapy has the following advantages: a broader spectrum of effect, synergistic effect, lesser risk of toxicity (it is possible to reduce doses), and decreased likelihood of resistance or tolerance. However, there is no evidence that it is possible to prevent resistance with CAF therapy. On the other hand, there are adverse antagonistic reactions, higher costs without any known benefit to the patient, and greater intensity of toxicity [391, 392], e.g. the risk of bone marrow suppression due to the accumulation of flucytosine in the event of renal failure caused by amphotericin B with a simultaneous supply of both antifungals [392, 393].

## Principles of combination therapy

Although the synergistic effect is the most desirable drug interaction, we can often observe occurrences of other interactions between antifungal drugs, i.e. addition, antagonism, or indifference [394]. However, in spite of the fact that multidrug therapy is common practice, especially in patients who do not respond to monotherapy, the assessment of in vitro and in vivo drug interactions may not always be adequate. Mathematical models have been developed to facilitate understanding the problem, to define, and to predict drug interactions. The most significant of them is empirical methods based on Bliss independence theory (effect-based strategy) and Loewe additivity theory (dose–effect-based strategy) [395, 396]. For both, if the effect of combination therapy is better than the expected (additive) effect, i.e. if the combination index (CI), which is defined as the ratio between the combined effect of drugs and the effect of individual components, is CI <1, we can speak of synergism. If the results are worse than expected (CI >1), there is an antagonistic effect of the drugs [395, 397].

It is possible to make an in vitro assessment of the efficacy of antifungal drugs administered in combination by applying methods determining drug sensitivity through the MIC. The following methods can be used:

1. The checkerboard method, including calculation of the fractional inhibitory concentration index (FICI). The FICI is determined for each drug by dividing the MIC of each drug used in combination by the MIC of each preparation used in monotherapy. It is suggested that an FICI of <0.5 should be considered as synergy, whereas an FICI of >4 should be regarded as antagonism, and an FICI of 0.5–4 should be regarded as no interaction [394, 398, 399].
  Results are shown as isobolograms. They present the nature of drug interaction and the range of concentrations where the maximum synergistic effect is achieved [400].
2. The time kill method: synergy—when a combination of drugs increases killing cells by ≥2 log10 (CFU/mL) at 24 h; in addition—when there is an increase of <2 but >1 log10; indifference—when there is a decrease from the least active antifungal <2 log10 CFU/mL; antagonism—a reduction in killing by >2 log10 [401, 402].
3. The Epsilometer test (Etest)—the diffusion-gradient method used to determine the minimal concentration of antibiotic inhibiting growth of the organism. With this technique, it is possible to accurately determine the degree of resistance to the drug and administering to the patient the optimal dose.
4. The response surface-modelling method: it is useful for drugs with different MIC values and for the assessment of drug interactions in infections with filamentous fungi [402, 403].

Animal models are used to verify the efficacy of combination therapy in vivo (histopathological assessment of target organs and survival rate), and subsequently, it is verified on humans (clinical state normalisation, laboratory investigations, and survival rate). It is necessary to note that it may be difficult to assess drug interdependence based on one ratio (interactions are often non-linear) [397] and the results of tests showing the in vitro efficacy of a particular interaction may not prove the same effect in the clinical situations [391].

CAF therapy is possible only thanks to the diverse mechanisms of action of individual drugs. At least three synergistic/additive models of action are possible:

1. The bioavailability model—one drug increases the availability and effective concentration of another drug in the target cell and/or place [404]. It may (a) facilitate another drug to enter fungal cells (destabilisation of the fungal cell membrane by azoles/polyenes and facilitation of the cell interior penetration by the flucytosine or (b) reduce degradation of another drug.
2. The same target model—when two drugs act on two different places of the same mechanism (both terbinafine and azoles inhibit ergosterol biosynthesis, and in consequence they damage the fungal cell membrane).
3. The parallel pathway inhibition model—drugs act on separate parts of the fungal cell which are responsible for crucial biological functions (echinocandins damage the fungal cell wall, whereas azoles and polyenes damage the cell membrane, both leading to cell lysis) [391, 397, 405].

The antagonistic mechanism is also possible—it is a competitive binding model [405], which assumes a mutually exclusive effect of both drugs at the same time (e.g. azoles inhibit the synthesis of ergosterol, which is necessary as the target of the polyene effect; thus polyenes become ineffective).

Antifungal drug combinations in different configurations are often concentration-dependent. Synergism can be observed at lower drug concentrations, whereas antagonism can be observed at higher concentrations [391]. CAF therapy with caspofungin (caspofungin plus voriconazole or caspofungin plus amphotericin B), which is applied in the case of *Aspergillus* infections, improves the effects of therapy if drugs are administered at adequate proportions. Synergism was observed when caspofungin was applied at smaller doses (1 mg/kg) than voriconazole and amphotericin B [406, 407], whereas the quantitative advantage of caspofungin in these combinations and larger doses (2.5–3 mg/kg) reduced positive effects [406–408]. A similar, concentration-dependent type of interaction was observed between amphotericin B and azoles [406, 409]. Although an antagonistic mechanism was observed in many studies [410, 411], the interaction between amphotericin B and fluconazole proves to be concentration-dependent, which is related to the pharmacodynamic effect. At larger doses, fluconazole reduces the content of ergosterol in the fungal cell membrane, and thus eliminates the target place of effect of amphotericin B. A reduced dose of fluconazole in CAF with amphotericin B recovers synergism and/or addition [412].

The aforementioned combination of amphotericin B with azoles arouses considerable controversy. Apart from the concentration-dependent mechanism, interactions between these drugs may depend on the administration time. The antagonism observed in the polyene-azole configuration is accounted for by the “depletion theory”, according to which an earlier supply of azoles reduces the amount of ergosterol, which is a prime target for amphotericin B [394]. On the other hand, the synergism in the polyenes-azoles combination is based on the “enhancement theory”, according to which the binding of amphotericin B with the fungal cell membrane sterols and the formation of pores facilitates the penetration of azoles into the cell interior, and thus inhibits the ergosterol synthesis by azoles [394]. A sequential supply of fluconazole and itraconazole reverses the antagonism in combination with amphotericin B. In many research models of invasive candidiasis and aspergillosis, the efficacy of CAF therapy based on amphotericin B and fluconazole was characterised by better survival rate than monotherapy [390, 413]. However, only when the sequence was changed and there was an earlier administration of amphotericin B before fluconazole, a synergistic effect was observed. As a result, pathogens were eliminated from the kidneys and heart at a faster rate in an animal model with pyelonephritis and endocarditis, as compared with traditional combination therapy, where amphotericin B and fluconazole were administered simultaneously [414]. An earlier administration of itraconazole reduces the efficacy of amphotericin B both in a conventional and lipid form in an animal model of invasive pulmonary aspergillosis. No antagonism was observed when the therapy was initiated with amphotericin B followed by subsequent itraconazole administration [415].

The considerations presented above contrast with the reports in which no such dependence was observed. Rex et al. researched non-neutropenic patients with candidaemia and noted that simultaneous administration of amphotericin B and fluconazole exhibited a trend to higher therapeutic efficacy than fluconazole monotherapy although the outcome was similar. It is noteworthy that no antagonism of the CAF therapy (fluconazole plus amphotericin B) was observed no matter if patients had received fluconazole before [416].

Thus, drug interactions depend on the type of preparations used in a combination therapy, the method used for the assessment of interaction, the genus and species of fungi, the time sequence of the administration of drugs and their doses [390].

## CAF therapy and clinical practice

### Candidiasis

So far there have not been too many indications to apply CAF therapy in *Candida* infections. A combination therapy based on lipid formulations of amphotericin B and flucytosine is recommended in the treatment of intracranial infections, including endophthalmitis [391]. A study on an animal model with *Candida* meningoencephalitis revealed that liposomal amphotericin B achieved higher concentration in the brain than amphotericin B lipid complex or amphotericin B deoxycholate [417].

A similar CAF formula is used for native valve endocarditis infected by a ventricular assist device (VAD), implantable cardioverter-defibrillator (ICD) or pacemaker [391, 418]. In spite of strong recommendation to use this combination of drugs, there is low quality of evidence concerning this issue. Since the introduction of echinocandins routine application of CAF therapy for candidaemia has not been advised [12]. There have been reports on fluconazole combined with flucytosine applied to a few patients in the treatment of *Candida* meningitis but it is generally thought that this CAF therapy is possible as a step-down therapy [419].

### Aspergillosis

CAF therapy applied in mould infections arouses big interest due to the high mortality it causes and due to its costs. There are contradictory conclusions of studies on the application of CAF in invasive aspergillosis. So far, research could not definitely confirm superiority of CAF. The results chiefly depend on the population of patients and their clinical state, the type of pathogen and its resistance, the location of infection (pulmonary versus extra-pulmonary), CAF heterogeneity, the presence of neutropenia, immunosuppressive regimens, the duration of therapy, various endpoints and follow-ups, etc. [420]. It is estimated that about 30–50% of ICU patients receive CAF therapy in invasive *Aspergillus* infections [421]. Although the caspofungin and liposomal amphotericin B combination was more successful as a primary rather than salvage therapy and it resulted in a favourable response in 60% patients [422] and despite the fact that the administration of voriconazole plus caspofungin as a primary therapy in organ transplant recipients improved the 90-day survival rate [423], there is no sufficient data to recommend routine application of CAF therapy as primary therapy in invasive *Aspergillus* infections [424].

A meta-analysis of 16 studies on 1833 patients shows that a double antifungal therapy increases the likelihood of therapeutic success and improves the 12-week survival rate, as compared with monotherapy in a salvage setting. Up to 30% of ICU patients receive this therapy for invasive aspergillosis (in breakthrough or refractory invasive aspergillosis) [397, 420, 421, 425].

The application of voriconazole and caspofungin combination as a salvage therapy (in the case of failure of amphotericin B therapy) caused synergistic interaction against *Aspergillus* (simultaneous inhibition of the cell membrane and fungal cell wall biosynthesis) and improved 3-month survival, as compared with voriconazole alone. The probability of death was the lowest in patients who received CAF therapy [426].

Although clinical trials did not reveal antagonism between azoles and amphotericin B, the application of this combination is not sufficiently justified and currently it is not recommended to administer polyenes and azoles to patients simultaneously [37].

CAF therapy based on voriconazole administered with anidulafungin [427] or micafungin [428, 429] had poor antifungal efficacy or missed statistical significance.

In a recently published study conducted on a homogenous group of haematological patients who received voriconazole and anidulafungin versus voriconazole alone, there was no significant difference in mortality between CAF and monotherapy. Nevertheless, the research proved that patients with a positive galactomannan test result and radiographic findings were characterised by reduced overall mortality in CAF therapy (15.7%) versus monotherapy (29.3%) (*P* = 0.037) [430].

Thus, in specific clinical situations (bilateral inflammatory infiltrations of the lungs, respiratory failure, sepsis/septic shock, unsuccessful monotherapy), the use of a triazole (voriconazole) or lipid amphotericin B formulation in combination with an echinocandin should be considered as salvage therapy [420, 421]. The type of antifungal applied in combination therapy depends on the *Aspergillus* species, organ function (chiefly kidney and liver function) and the need of other drugs which may cause drug–drug interactions, especially with voriconazole (voriconazole is metabolised by, and inhibits enzymes in the cytochrome system P450: CYP2C19, CYP2C9 and CYP3A4) [421, 431]. To sum up, CAF therapy for invasive aspergillosis is most recommended in patients with haematological malignancies and an elevated galactomannan levels. Apart from that, it seems that in CAF therapy for invasive *Aspergillus* infections voriconazole plus echinocandin or amphotericin B combined with an echinocandin are preferable combinations. CAF can be used as a salvage therapy in high risk patients [432]. However, in the recently published guidelines CAF therapy with voriconazole and an echinocandin is discouraged, but may be considered as a primary management in select patients with documented invasive aspergillosis (weak recommendation) [10].

### Cryptococcus

The combination of amphotericin B and flucytosine became a standard in treating cryptococcal meningitis [433] when Bennett et al. confirmed that the combination was more efficacious than amphotericin B administered as a monotherapy [434]. CAF therapy resulted in lesser toxicity and significantly faster sterilisation of CSF. There were similar results of other studies on cryptococcal meningitis in patients infected with human immunodeficiency virus (HIV) [435]. The combination of amphotericin B and flucytosine resulted in reduced risk of mortality on the 14th and 70th day in comparison with patients receiving amphotericin B as a monotherapy and it exhibited improved early fungicidal activity (EFA) [435]. Flucytosine quickly diffuses into the CSF; hence its efficacy in treating intracranial fungal infections [436]. O’Connor and colleagues found that the combination of liposomal amphotericin B and flucytosine applied to treat cryptococcal meningoencephalitis exhibited additive effect in the central nervous system and resulted in a dose-dependent reduction of the fungal burden. The administration of liposomal amphotericin B dosed at 3 mg/kg/day plus flucytosine 50 mg/kg/day, and liposomal amphotericin B 3 mg/kg/day plus flucytosine 100 mg/kg/day achieved near-maximum antifungal activity and it was significantly less toxic [437]. The role of azoles (fluconazole) in treatment of cryptococcal infections is usually limited to a maintenance therapy in the form of monotherapy after combined induction therapy [438]. However, due to the unavailability of flucytosine in some countries a combination of a short administration of amphotericin B and larger doses of fluconazole (1200 mg/day) is used. The combination resulted in improved EFA in the CSF and lower toxicity of the therapy [432, 439]. If it was impossible to apply a lipid formulation of amphotericin B, CAF therapy based on fluconazole and flucytosine was applied. It improved the survival rate and reduced the time of CSF sterilisation [440]. Thus, in cryptococcal diseases (meningitis, encephalitis) CAF therapy with amphotericin B plus flucytosine remains a treatment of choice, especially in HIV-infected patients. If flucytosine is unavailable, fluconazole is recommended as an alternative [389, 432].

### Zygomycosis

Zygomycosis is a rare infection due to mould fungi of the *Zygomycota*, preferentially called mucormycosis. *Zygomycota* cause a life-threatening infection involving most commonly lung and rhino-orbital-cerebral locations, mostly in immunocompromised patients (neutropenia, immunosuppressive drugs, penetrating trauma, diabetes) [441]. Despite therapy (elimination and/or reversal of underlying risk factors, surgical debridement, antifungals), the overall mortality is >50% and approaches 100% in disseminated forms [441]. The standard medical therapy includes liposomal amphotericin B, which is the most effective agent, whereas in the case of refractory disease or intolerance to prior antifungal therapy, CAF therapy is postulated, though with a moderate strength recommendation [11]. The use of polyene–caspofungin CAF therapy is interesting from the point of view of the mechanism of action of caspofungin, which in filamentous fungi is rather fungistatic. In vitro caspofungin is inactive against *Mucorales*, but in combination with liposomal amphotericin B enhances its action in some species, especially in relation to *Rhizopus* spp., the most common identified pathogens. Namely, 1,3-β-d-glucan synthase in yeast cells contains a regulatory subunit encoded by RHO1 and a catalytic subunit encoded by FKS, which is simultaneously the target for echinocandins. Thus, membrane-associated 1,3-β-d-glucan synthase activity is inhibited by caspofungin, which in combination with liposomal amphotericin B significantly improves the outcomes and long-term survival compared to monotherapy [441, 442]. Within the other antifungals, posaconazole seems to have the most effective activity against *Mucorales*. The analyses suggest that a CAF treatment with liposomal amphotericin B and posaconazole may be considered when dealing with patients presenting highly aggressive forms of an invasive mucormycosis [443]. In case of amphotericin B intolerance, CAF therapy with caspofungin plus posaconazole has been applied with positive effect. Sheybani et al. present two cases treated successfully with this combination and suggest that caspofungin might affect the efficacy of posaconazole in an additive or even synergistic manner, although *Mucorales* are resistant to caspofungin [444].

## CAF therapy: conclusions

In spite of contradictory reports numerous publications prove that CAF therapy may have positive effects in treating selected groups of patients. CAF therapy based on amphotericin B plus flucytosine proved to be efficacious in treating cryptococcal disease, particularly in HIV-infected patients. Voriconazole combined with anidulafungin was efficacious in treating haematological patients with invasive aspergillosis, mostly with a positive galactomannan test, and voriconazole plus echinocandin was efficacious in salvage settings in invasive aspergillosis. The application of CAF therapy in the treatment of *Candida* infections is significantly limited, but current guidelines advocate the application of this therapy to treat CNS candidiasis and *Candida* endocarditis (amphotericin B plus flucytosine). In the case of the refractory form of zygomycosis, salvage therapy with the combination of posaconazole and liposomal amphotericin B is recommended with moderate support by current guidelines. Polyene plus caspofungin is postulated, but is only marginally supported by the guidelines. In the case of amphotericin B intolerance, concurrent therapy with posaconazole and caspofungin can be considered. The advantage of CAF therapy over monotherapy was not proved for other fungal infections [432].

## References

[1] Meersseman W, Vandecasteele SJ, Wilmer A, Verbeken E, Peetermans WE, Van Wijngaerden E (2004). Invasive aspergillosis in critically ill patients without malignancy. Am J Respir Crit Care Med.

[2] Krieter P, Flannery B, Musick T, Gohdes M, Martinho M, Courtney R (2004). Disposition of posaconazole following single-dose oral administration in healthy subjects. Antimicrob Agents Chemother.

[3] Pea F (2012). Plasma pharmacokinetics of antimicrobial agents in critically ill patients. Curr Clin Pharmacol..

[4] Lepak AJ, Andes DR (2011). Antifungal PK/PD considerations in fungal pulmonary infections. Semin Respir Crit Care Med..

[5] Felton T, Troke PF, Hope WW (2014). Tissue penetration of antifungal agents. Clin Microbiol Rev.

[6] Utz JP, Treger A, McCullough NB, Emmons CW (1958). Amphotericin B: intravenous use in 21 patients with systemic fungal diseases. Antibiot Annu..

[7] Lemke A, Kiderlen AF, Kayser O (2005). Amphotericin B. Appl Microbiol Biotechnol.

[8] Brajtburg J, Bolard J (1996). Carrier effects on biological activity of amphotericin B. Clin Microbiol Rev.

[9] Ellis D (2002). Amphotericin B: spectrum and resistance. J Antimicrob Chemother.

[10] Patterson TF, Thompson GR, Denning DW, Fishman JA, Hadley S, Herbrecht R (2016). Practice guidelines for the diagnosis and management of aspergillosis: 2016 update by the Infectious Diseases Society of America. Clin Infect Dis.

[11] Cornely OA, Arikan-Akdagli S, Dannaoui E, Groll AH, Lagrou K, Chakrabarti A (2014). ESCMID and ECMM joint clinical guidelines for the diagnosis and management of mucormycosis 2013. Clin Microbiol Infect.

[12] Pappas PG, Kauffman CA, Andes DR, Clancy CJ, Marr KA, Ostrosky-Zeichner L (2016). Clinical practice guideline for the management of candidiasis: 2016 update by the Infectious Diseases Society of America. Clin Infect Dis.

[13] Cornely OA, Bassetti M, Calandra T, Garbino J, Kullberg BJ, Lortholary O (2012). ESCMID* guideline for the diagnosis and management of *Candida* diseases 2012: non-neutropenic adult patients. Clin Microbiol Infect.

[14] Prigitano A, Cavanna C, Passera M, Ossi C, Sala E, Lombardi G (2016). CAND-LO 2014-15 study: changing epidemiology of candidemia in Lombardy (Italy). Infection.

[15] Laniado-Laborín R, Cabrales-Vargas MN (2009). Amphotericin B: side effects and toxicity. Rev Iberoam Micol..

[16] Walsh TJ, Finberg RW, Arndt C, Hiemenz J, Schwartz C, Bodensteiner D, National Institute of Allergy and Infectious Diseases Mycoses Study Group (1999). Liposomal amphotericin B for empirical therapy in patients with persistent fever and neutropenia. N Engl J Med.

[17] Roden MM, Nelson LD, Knudsen TA, Jarosinski PF, Starling JM, Shiflett SE (2003). Triad of acute infusion-related reactions associated with liposomal amphotericin B: analysis of clinical and epidemiological characteristics. Clin Infect Dis.

[18] Harbarth S, Burke JP, Lloyd JF, Evans RS, Pestotnik SL, Samore MH (2002). Clinical and economic outcomes of conventional amphotericin B-associated nephrotoxicity. Clin Infect Dis.

[19] Bowden R, Chandrasekar P, White MH, Li X, Pietrelli L, Gurwith M (2002). A double-blind, randomized, controlled trial of amphotericin B colloidal dispersion versus amphotericin B for treatment of invasive aspergillosis in immunocompromised patients. Clin Infect Dis.

[20] Wingard JR, Kubilis P, Lee L, Yee G, White M, Walshe L (1999). Clinical significance of nephrotoxicity in patients treated with amphotericin B for suspected or proven aspergillosis. Clin Infect Dis.

[21] Fanos V, Cataldi L (2000). Amphotericin B-induced nephrotoxicity: a review. J Chemother.

[22] Eriksson U, Seifert B, Schaffner A (2001). Comparison of effects of amphotericin B deoxycholate infused over 4 or 24 hours: randomised controlled trial. BMJ.

[23] Girois SB, Chapuis F, Decullier E, Revol BG (2005). Adverse effects of antifungal therapies in invasive fungal infections: review and meta-analysis. Eur J Clin Microbiol Infect Dis.

[24] Bates DW, Su L, Yu DT, Chertow GM, Seger DL, Gomes DR (2001). Correlates of acute renal failure in patients receiving parenteral amphotericin B. Kidney Int.

[25] Bates DW, Su L, Yu DT, Chertow GM, Seger DL, Gomes DR (2001). Mortality and costs of acute renal failure associated with amphotericin B therapy. Clin Infect Dis.

[26] Deray G (2002). Amphotericin B nephrotoxicity. J Antimicrob Chemother..

[27] Barcia JP (1998). Hyperkalemia associated with rapid infusion of conventional and lipid complex formulations of amphotericin B. Pharmacotherapy..

[28] Bekersky I, Fielding RM, Dressler DE, Lee JW, Buell DN, Walsh TJ (2002). Plasma protein binding of amphotericin B and pharmacokinetics of bound versus unbound amphotericin B after administration of intravenous liposomal amphotericin B (AmBisome) and amphotericin B deoxycholate. Antimicrob Agents Chemother.

[29] Ridente Y, Aubard J, Bolard J (1999). Absence in amphotericin B-spiked human plasma of the free monomeric drug, as detected by SERS. FEBS Lett.

[30] Atkinson AJ, Bennett JE (1978). Amphotericin B pharmacokinetics in humans. Antimicrob Agents Chemother.

[31] Hoeprich PD (1990). Elimination half-life of amphotericin B. J Infect.

[32] Kan VL, Bennett JE, Amantea MA, Smolskis MC, McManus E, Grasela DM (1991). Comparative safety, tolerance, and pharmacokinetics of amphotericin B lipid complex and amphotericin B desoxycholate in healthy male volunteers. J Infect Dis.

[33] Ayestarán A, López RM, Montoro JB, Estíbalez A, Pou L, Julià A (1996). Pharmacokinetics of conventional formulation versus fat emulsion formulation of amphotericin B in a group of patients with neutropenia. Antimicrob Agents Chemother.

[34] Heinemann V, Bosse D, Jehn U, Kähny B, Wachholz K, Debus A (1997). Pharmacokinetics of liposomal amphotericin B (Ambisome) in critically ill patients. Antimicrob Agents Chemother.

[35] Bekersky I, Fielding RM, Dressler DE, Lee JW, Buell DN, Walsh TJ (2002). Pharmacokinetics, excretion, and mass balance of liposomal amphotericin B (AmBisome) and amphotericin B deoxycholate in humans. Antimicrob Agents Chemother.

[36] Bellmann R (2007). Clinical pharmacokinetics of systemically administered antimycotics. Curr Clin Pharmacol..

[37] Bellmann R (2013). Pharmacodynamics and pharmacokinetics of antifungals for treatment of invasive aspergillosis. Curr Pharm Des.

[38] Chabot GG, Pazdur R, Valeriote FA, Baker LH (1989). Pharmacokinetics and toxicity of continuous infusion amphotericin B in cancer patients. J Pharm Sci.

[39] Peleg AY, Woods ML (2004). Continuous and 4 h infusion of amphotericin B: a comparative study involving high-risk haematology patients. J Antimicrob Chemother.

[40] Speich R, Dutly A, Naef R, Russi EW, Weder W, Boehler A (2002). Tolerability, safety and efficacy of conventional amphotericin B administered by 24-hour infusion to lung transplant recipients. Swiss Med Wkly..

[41] Furrer K, Schaffner A, Vavricka SR, Halter J, Imhof A, Schanz U (2002). Nephrotoxicity of cyclosporine A and amphotericin B-deoxycholate as continuous infusion in allogenic stem cell transplantation. Swiss Med Wkly..

[42] Falci DR, Lunardi LW, Ramos CG, Bay MB, Aquino VR, Goldani LZ (2010). Continuous infusion of amphotericin B deoxycholate in the treatment of cryptococcal meningoencephalitis: analysis of safety and fungicidal activity. Clin Infect Dis.

[43] Falci DR, dos Santos RP, Wirth F, Goldani LZ (2011). Continuous infusion of amphotericin B deoxycholate: an innovative, low-cost strategy in antifungal treatment. Mycoses.

[44] Maharom P, Thamlikitkul V (2006). Implementation of clinical practice policy on the continuous intravenous administration of amphotericin B deoxycholate. J Med Assoc Thai.

[45] Wiederhold NP, Tam VH, Chi J, Prince RA, Kontoyiannis DP, Lewis RE (2006). Pharmacodynamic activity of amphotericin B deoxycholate is associated with peak plasma concentrations in a neutropenic murine model of invasive pulmonary aspergillosis. Antimicrob Agents Chemother.

[46] Bellmann R, Egger P, Gritsch W, Bellmann-Weiler R, Joannidis M, Kaneider N (2003). Amphotericin B lipid formulations in critically ill patients on continuous veno-venous haemofiltration. J Antimicrob Chemother.

[47] Al-Quadeib BT, Radwan MA, Siller L, Mutch E, Horrocks B, Wright M (2014). Therapeutic monitoring of amphotericin B in Saudi ICU patients using UPLC MS/MS assay. Biomed Chromatogr.

[48] Adler-Moore JP (1993). T PR. Development, characterization, efficacy and mode of action of Am Bisome, a unilamellar liposomal formulation of amphotericin B. J Liposome Res.

[49] Guo LS (2001). Amphotericin B colloidal dispersion: an improved antifungal therapy. Adv Drug Deliv Rev.

[50] Boswell GW, Buell D, Bekersky I (1998). Am Bisome (liposomal amphotericin B): a comparative review. J Clin Pharmacol.

[51] Heinemann V, Kähny B, Debus A, Wachholz K, Jehn U (1994). Pharmacokinetics of liposomal amphotericin B (AmBisome) versus other lipid-based formulations. Bone Marrow Transplant.

[52] Walsh TJ, Yeldandi V, McEvoy M, Gonzalez C, Chanock S, Freifeld A (1998). Safety, tolerance, and pharmacokinetics of a small unilamellar liposomal formulation of amphotericin B (AmBisome) in neutropenic patients. Antimicrob Agents Chemother.

[53] Gokhale PC, Barapatre RJ, Advani SH, Kshirsagar NA, Pandya SK (1993). Pharmacokinetics and tolerance of liposomal amphotericin B in patients. J Antimicrob Chemother.

[54] Adedoyin A, Bernardo JF, Swenson CE, Bolsack LE, Horwith G, DeWit S (1997). Pharmacokinetic profile of ABELCET (amphotericin B lipid complex injection): combined experience from phase I and phase II studies. Antimicrob Agents Chemother.

[55] Adedoyin A, Swenson CE, Bolcsak LE, Hellmann A, Radowska D, Horwith G (2000). A pharmacokinetic study of amphotericin B lipid complex injection (Abelcet) in patients with definite or probable systemic fungal infections. Antimicrob Agents Chemother.

[56] Walsh TJ, Goodman JL, Pappas P, Bekersky I, Buell DN, Roden M (2001). Safety, tolerance, and pharmacokinetics of high-dose liposomal amphotericin B (AmBisome) in patients infected with Aspergillus species and other filamentous fungi: maximum tolerated dose study. Antimicrob Agents Chemother.

[57] Bellmann R, Egger P, Wiedermann CJ (2003). Differences in pharmacokinetics of amphotericin B lipid formulations despite clinical equivalence. Clin Infect Dis.

[58] Humphreys H, Oliver DA, Winter R, Warnock DW (1994). Liposomal amphotericin B and continuous venous–venous haemofiltration. J Antimicrob Chemother.

[59] Tomlin M, Priestley GS (1995). Elimination of liposomal amphotericin by hemodiafiltration. Intensive Care Med.

[60] Bellmann R, Egger P, Djanani A, Wiedermann CJ (2004). Pharmacokinetics of amphotericin B lipid complex in critically ill patients on continuous veno-venous haemofiltration. Int J Antimicrob Agents.

[61] Weiler S, Uberlacher E, Schöfmann J, Stienecke E, Dunzendorfer S, Joannidis M (2012). Pharmacokinetics of amphotericin B colloidal dispersion in critically ill patients with cholestatic liver disease. Antimicrob Agents Chemother.

[62] Vogelsinger H, Joannidis M, Kountchev J, Bellmann-Weiler R, Wiedermann CJ, Bellmann R (2006). Pharmacokinetics of liposomal amphotericin B during extracorporeal albumin dialysis. Artif Organs.

[63] Weiler S, Vogelsinger H, Joannidis M, Dunzendorfer S, Bellmann R (2011). Influence of albumin dialysis on pharmacokinetics of amphotericin B colloidal dispersion and amphotericin B lipid complex. Artif Organs.

[64] Ruiz S, Papy E, Da Silva D, Nataf P, Massias L, Wolff M (2009). Potential voriconazole and caspofungin sequestration during extracorporeal membrane oxygenation. Intensive Care Med.

[65] Sanders SW, Buchi KN, Goddard MS, Lang JK, Tolman KG (1991). Single-dose pharmacokinetics and tolerance of a cholesteryl sulfate complex of amphotericin B administered to healthy volunteers. Antimicrob Agents Chemother.

[66] Hiemenz JW, Walsh TJ (1996). Lipid formulations of amphotericin B: recent progress and future directions. Clin Infect Dis.

[67] Janknegt R, de Marie S, Bakker-Woudenberg IA, Crommelin DJ (1992). Liposomal and lipid formulations of amphotericin B. Clin Pharmacokinet..

[68] Frothingham R (2002). Lipid formulations of amphotericin B for empirical treatment of fever and neutropenia. Clin Infect Dis.

[69] Wingard JR (2002). Lipid formulations of amphotericins: are you a lumper or a splitter?. Clin Infect Dis.

[70] Wingard JR, White MH, Anaissie E, Raffalli J, Goodman J, Arrieta A (2000). A randomized, double-blind comparative trial evaluating the safety of liposomal amphotericin B versus amphotericin B lipid complex in the empirical treatment of febrile neutropenia. L Amph/ABLC Collaborative Study Group. Clin Infect Dis.

[71] Fleming RV, Kantarjian HM, Husni R, Rolston K, Lim J, Raad I (2001). Comparison of amphotericin B lipid complex (ABLC) vs. ambisome in the treatment of suspected or documented fungal infections in patients with leukemia. Leuk Lymphoma.

[72] Pahls S, Schaffner A (1994). Comparison of the activity of free and liposomal amphotericin B in vitro and in a model of systemic and localized murine candidiasis. J Infect Dis.

[73] Leenders AC, Reiss P, Portegies P, Clezy K, Hop WC, Hoy J (1997). Liposomal amphotericin B (AmBisome) compared with amphotericin B both followed by oral fluconazole in the treatment of AIDS-associated cryptococcal meningitis. AIDS..

[74] Leenders AC, Daenen S, Jansen RL, Hop WC, Lowenberg B, Wijermans PW (1998). Liposomal amphotericin B compared with amphotericin B deoxycholate in the treatment of documented and suspected neutropenia-associated invasive fungal infections. Br J Haematol.

[75] Working PK (1999). Amphotericin B colloidal dispersion. Chemotherapy..

[76] White MH, Bowden RA, Sandler ES, Graham ML, Noskin GA, Wingard JR (1998). Randomized, double-blind clinical trial of amphotericin B colloidal dispersion vs. amphotericin B in the empirical treatment of fever and neutropenia. Clin Infect Dis.

[77] Wasan KM, Lopez-Berestein G (1996). Characteristics of lipid-based formulations that influence their biological behavior in the plasma of patients. Clin Infect Dis.

[78] Collette N, van der Auwera P, Lopez AP, Heymans C, Meunier F (1989). Tissue concentrations and bioactivity of amphotericin B in cancer patients treated with amphotericin B-deoxycholate. Antimicrob Agents Chemother.

[79] Wong-Beringer A, Jacobs RA, Guglielmo BJ (1998). Lipid formulations of amphotericin B: clinical efficacy and toxicities. Clin Infect Dis.

[80] Adler-Moore J, Proffitt RT (2002). Am Bisome: liposomal formulation, structure, mechanism of action and pre-clinical experience. J Antimicrob Chemother.

[81] Bekersky I, Fielding RM, Dressler DE, Kline S, Buell DN, Walsh TJ (2001). Pharmacokinetics, excretion, and mass balance of 14C after administration of 14C-cholesterol-labeled Am Bisome to healthy volunteers. J Clin Pharmacol.

[82] Swenson CE, Perkins WR, Roberts P, Ahmad I, Stevens R, Stevens DA (1998). In vitro and in vivo antifungal activity of amphotericin B lipid complex: are phospholipases important?. Antimicrob Agents Chemother.

[83] Gottfredsson M, Jessup CJ, Cox GM, Perfect JR, Ghannoum MA (2001). Fungal phospholipase activity and susceptibility to lipid preparations of amphotericin B. Antimicrob Agents Chemother.

[84] Kennedy AL, Wasan KM (1999). Preferential distribution of amphotericin B lipid complex into human HDL3 is a consequence of high density lipoprotein coat lipid content. J Pharm Sci.

[85] Christiansen KJ, Bernard EM, Gold JW, Armstrong D (1985). Distribution and activity of amphotericin B in humans. J Infect Dis.

[86] Vogelsinger H, Weiler S, Djanani A, Kountchev J, Bellmann-Weiler R, Wiedermann CJ (2006). Amphotericin B tissue distribution in autopsy material after treatment with liposomal amphotericin B and amphotericin B colloidal dispersion. J Antimicrob Chemother.

[87] Wu JQ, Shao K, Wang X, Wang RY, Cao YH, Yu YQ (2014). In vitro and in vivo evidence for amphotericin B as a P-glycoprotein substrate on the blood–brain barrier. Antimicrob Agents Chemother.

[88] Stevens DA, Clemons KV, Martinez M, Chen V (2015). The brain, amphotericin B, and P-glycoprotein. Antimicrob Agents Chemother.

[89] Weiler S, Falkensammer G, Hammerer-Lercher A, Anliker M, Vogelsinger H, Joannidis M (2009). Pulmonary epithelial lining fluid concentrations after use of systemic amphotericin B lipid formulations. Antimicrob Agents Chemother.

[90] Weiler S, Bellmann-Weiler R, Joannidis M, Bellmann R (2007). Penetration of amphotericin B lipid formulations into pleural effusion. Antimicrob Agents Chemother.

[91] van der Voort PH, Boerma EC, Yska JP (2007). Serum and intraperitoneal levels of amphotericin B and flucytosine during intravenous treatment of critically ill patients with *Candida* peritonitis. J Antimicrob Chemother.

[92] Weiler S, Bellmann-Weiler R, Dunzendorfer S, Joannidis M, Bellmann R (2008). Levels of amphotericin B lipid formulations in ascites. J Antimicrob Chemother.

[93] Adamson PC, Rinaldi MG, Pizzo PA, Walsh TJ (1989). Amphotericin B in the treatment of *Candida* cholecystitis. Pediatr Infect Dis J..

[94] Duflo F, Allaouchiche B, Chassard D (2000). Biliary excretion of amphotericin B deoxycholate and amphotericin B lipid complex. Scand J Infect Dis.

[95] Welte R, Eschertzhuber S, Weiler S, Leitner-Rupprich S, Aigner M, Lass-Florl C (2015). Biliary amphotericin B pharmacokinetics and pharmacodynamics in critically ill liver transplant recipients receiving treatment with amphotericin B lipid formulations. Int J Antimicrob Agents.

[96] Tassel D, Madoff MA (1968). Treatment of *Candida* sepsis and Cryptococcus meningitis with 5-fluorocytosine. A new antifungal agent. JAMA..

[97] Vermes A, Guchelaar HJ, Dankert J (2000). Flucytosine: a review of its pharmacology, clinical indications, pharmacokinetics, toxicity and drug interactions. J Antimicrob Chemother.

[98] Cutler RE, Blair AD, Kelly MR (1978). Flucytosine kinetics in subjects with normal and impaired renal function. Clin Pharmacol Ther.

[99] Block ER, Bennett JE, Livoti LG, Klein WJ, MacGregor RR, Henderson L (1974). Flucytosine and amphotericin B: hemodialysis effects on the plasma concentration and clearance. Ann Intern Med..

[100] Block ER, Bennett JE (1972). Pharmacological studies with 5-fluorocytosine. Antimicrob Agents Chemother.

[101] Daneshmend TK, Warnock DW (1983). Clinical pharmacokinetics of systemic antifungal drugs. Clin Pharmacokinet.

[102] Drouhet E, Babinet P, Chapusot JP, Kleinknecht D (1973). 5-Fluorocytosine in the treatment of candidiasis with acute renal insufficiency: its kinetics during haemodialysis and peritoneal dialysis. Biomedicine..

[103] Schönebeck J, Polak A, Fernex M, Scholer HJ (1973). Pharmacokinetic studies on the oral antimycotic agent 5-fluorocytosine in individuals with normal and impaired kidney function. Chemotherapy.

[104] Ittel TH, Legler UF, Polak A, Glöckner WM, Sieberth HG (1987). 5-Fluorocytosine kinetics in patients with acute renal failure undergoing continuous hemofiltration. Chemotherapy.

[105] Thomson AH, Shankland G, Clareburt C, Binning S (2002). Flucytosine dose requirements in a patient receiving continuous veno-venous haemofiltration. Intensive Care Med.

[106] Kunka ME, Cady EA, Woo HC, Thompson Bastin ML (2015). Flucytosine pharmacokinetics in a critically ill patient receiving continuous renal replacement therapy. Case Rep Crit Care..

[107] Roberts JA, Udy AA, O’Donoghue S, Briscoe S, Paterson DL, Lipman J (2009). Clearance of intravenous 5-fluorocytosine during continuous venovenous haemodiafiltration in a patient with hepatosplenic candidiasis. Int J Antimicrob Agents.

[108] Polak A (1979). Pharmacokinetics of amphotericin B and flucytosine. Postgrad Med J.

[109] Block ER (1973). Effect of hepatic insufficiency on 5-fluorocytosine concentrations in serum. Antimicrob Agents Chemother.

[110] Pennington JE, Block ER, Reynolds HY (1974). 5-fluorocytosine and amphotericin B in bronchial secretions. Antimicrob Agents Chemother.

[111] Meletiadis J, Al-Saigh R, Velegraki A, Walsh TJ, Roilides E, Zerva L (2012). Pharmacodynamic effects of simulated standard doses of antifungal drugs against Aspergillus species in a new in vitro pharmacokinetic/pharmacodynamic model. Antimicrob Agents Chemother.

[112] Mavridou E, Bruggemann RJ, Melchers WJ, Verweij PE, Mouton JW (2010). Impact of cyp51A mutations on the pharmacokinetic and pharmacodynamic properties of voriconazole in a murine model of disseminated aspergillosis. Antimicrob Agents Chemother.

[113] Lomaestro BM, Piatek MA (1998). Update on drug interactions with azole antifungal agents. Ann Pharmacother.

[114] Albengres E, Le Louët H, Tillement JP (1998). Systemic antifungal agents. Drug interactions of clinical significance. Drug Saf.

[115] Shi J, Chapel S, Montay G, Hardy P, Barrett JS, Sica D (2005). Effect of ketoconazole on the pharmacokinetics and safety of telithromycin and clarithromycin in older subjects with renal impairment. Int J Clin Pharmacol Ther.

[116] Kovarik JM, Beyer D, Bizot MN, Jiang Q, Shenouda M, Schmouder RL (2005). Blood concentrations of everolimus are markedly increased by ketoconazole. J Clin Pharmacol.

[117] Chaikin P, Gillen MS, Malik M, Pentikis H, Rhodes GR, Roberts DJ (2005). Co-administration of ketoconazole with H1-antagonists ebastine and loratadine in healthy subjects: pharmacokinetic and pharmacodynamic effects. Br J Clin Pharmacol.

[118] Park JY, Kim KA, Shin JG, Lee KY (2004). Effect of ketoconazole on the pharmacokinetics of rosiglitazone in healthy subjects. Br J Clin Pharmacol.

[119] Liu B, Crewe HK, Ozdemir M, Rowland Yeo R, Tucker G, Rostami-Hodjegan A (2017). The absorption kinetics of ketoconazole plays a major role in explaining the reported variability in the level of interaction with midazolam: interplay between formulation and inhibition of gut wall and liver metabolism. Biopharm Drug Dispos.

[120] Townsend R, Dietz A, Hale C, Akhtar S, Kowalski D, Lademacher C (2017). Pharmacokinetic evaluation of CYP3A4-mediated drug–drug interactions of isavuconazole with rifampin, ketoconazole, midazolam, and ethinyl estradiol/norethindrone in healthy adults. Clin Pharmacol Drug Dev..

[121] Becker C, Frey R, Unger S, Thomas D, Reber M, Weimann G (2016). Pharmacokinetic interaction of riociguat with ketoconazole, clarithromycin, and midazolam. Pulm Circ..

[122] Wiesinger H, Berse M, Klein S, Gschwend S, Höchel J, Zollmann FS (2015). Pharmacokinetic interaction between the CYP3A4 inhibitor ketoconazole and the hormone drospirenone in combination with ethinylestradiol or estradiol. Br J Clin Pharmacol.

[123] Stott C, White L, Wright S, Wilbraham D, Guy G (2013). A phase I, open-label, randomized, crossover study in three parallel groups to evaluate the effect of Rifampicin, Ketoconazole, and Omeprazole on the pharmacokinetics of THC/CBD oromucosal spray in healthy volunteers. Springerplus..

[124] Van Tyle JH (1984). Ketoconazole. Mechanism of action, spectrum of activity, pharmacokinetics, drug interactions, adverse reactions and therapeutic use. Pharmacotherapy..

[125] Figg WD, Liu Y, Arlen P, Gulley J, Steinberg SM, Liewehr DJ (2005). A randomized, phase II trial of ketoconazole plus alendronate versus ketoconazole alone in patients with androgen independent prostate cancer and bone metastases. J Urol.

[126] Gupta AK, Kohli Y, Batra R (2005). In vitro activities of posaconazole, ravuconazole, terbinafine, itraconazole and fluconazole against dermatophyte, yeast and non-dermatophyte species. Med Mycol.

[127] Cordonnier C (2004). Fungal infections: current diagnosis and treatment. Hematol J..

[128] Humphrey MJ, Jevons S, Tarbit MH (1985). Pharmacokinetic evaluation of UK-49,858, a metabolically stable triazole antifungal drug, in animals and humans. Antimicrob Agents Chemother.

[129] Debruyne D, Ryckelynck JP (1993). Clinical pharmacokinetics of fluconazole. Clin Pharmacokinet.

[130] Buijk SL, Gyssens IC, Mouton JW, Verbrugh HA, Touw DJ, Bruining HA (2001). Pharmacokinetics of sequential intravenous and enteral fluconazole in critically ill surgical patients with invasive mycoses and compromised gastro-intestinal function. Intensive Care Med.

[131] Debruyne D, Ryckelynck JP, Moulin M, Hurault de Ligny B, Levaltier B, Bigot MC (1990). Pharmacokinetics of fluconazole in patients undergoing continuous ambulatory peritoneal dialysis. Clin Pharmacokinet.

[132] Toon S, Ross CE, Gokal R, Rowland M (1990). An assessment of the effects of impaired renal function and haemodialysis on the pharmacokinetics of fluconazole. Br J Clin Pharmacol.

[133] Wysowski DK, Bacsanyi J (1996). Cisapride and fatal arrhythmia. N Engl J Med.

[134] Honig PK, Worham DC, Zamani K, Mullin JC, Conner DP, Cantilena LR (1993). The effect of fluconazole on the steady-state pharmacokinetics and electrocardiographic pharmacodynamics of terfenadine in humans. Clin Pharmacol Ther.

[135] Schöttker B, Dösch A, Kraemer DM (2003). Severe hepatotoxicity after application of desloratadine and fluconazole. Acta Haematol.

[136] Lazar JD, Wilner KD (1990). Drug interactions with fluconazole. Rev Infect Dis.

[137] Ehninger G, Jaschonek K, Schuler U, Krüger HU (1989). Interaction of fluconazole with cyclosporin. Lancet.

[138] Krüger HU, Schuler U, Zimmermann R, Ehninger G (1989). Absence of significant interaction of fluconazole with cyclosporin. J Antimicrob Chemother.

[139] López-Gil JA (1993). Fluconazole-cyclosporine interaction: a dose-dependent effect?. Ann Pharmacother.

[140] Canafax DM, Graves NM, Hilligoss DM, Carleton BC, Gardner MJ, Matas AJ (1991). Interaction between cyclosporine and fluconazole in renal allograft recipients. Transplantation.

[141] Torregrosa V, De la Torre M, Campistol JM, Oppenheimer F, Ricart MJ, Vilardell J (1992). Interaction of fluconazole with ciclosporin A. Nephron..

[142] Mañez R, Martin M, Raman D, Silverman D, Jain A, Warty V (1994). Fluconazole therapy in transplant recipients receiving FK506. Transplantation.

[143] Osowski CL, Dix SP, Lin LS, Mullins RE, Geller RB, Wingard JR (1996). Evaluation of the drug interaction between intravenous high-dose fluconazole and cyclosporine or tacrolimus in bone marrow transplant patients. Transplantation.

[144] Sádaba B, Campanero MA, Quetglas EG, Azanza JR (2004). Clinical relevance of sirolimus drug interactions in transplant patients. Transplant Proc..

[145] Kerr HD (1993). Case report: potentiation of warfarin by fluconazole. Am J Med Sci.

[146] Crussell-Porter LL, Rindone JP, Ford MA, Jaskar DW (1993). Low-dose fluconazole therapy potentiates the hypoprothrombinemic response of warfarin sodium. Arch Intern Med.

[147] Cadle RM, Zenon GJ, Rodriguez-Barradas MC, Hamill RJ (1994). Fluconazole-induced symptomatic phenytoin toxicity. Ann Pharmacother.

[148] Howitt KM, Oziemski MA (1989). Phenytoin toxicity induced by fluconazole. Med J Aust.

[149] Mitchell AS, Holland JT (1989). Fluconazole and phenytoin: a predictable interaction. BMJ.

[150] Kramer MR, Marshall SE, Denning DW, Keogh AM, Tucker RM, Galgiani JN (1990). Cyclosporine and itraconazole interaction in heart and lung transplant recipients. Ann Intern Med.

[151] Olkkola KT, Ahonen J, Neuvonen PJ (1996). The effects of the systemic antimycotics, itraconazole and fluconazole, on the pharmacokinetics and pharmacodynamics of intravenous and oral midazolam. Anesth Analg.

[152] Apseloff G, Hilligoss DM, Gardner MJ, Henry EB, Inskeep PB, Gerber N (1991). Induction of fluconazole metabolism by rifampin: in vivo study in humans. J Clin Pharmacol.

[153] Nicolau DP, Crowe HM, Nightingale CH, Quintiliani R (1995). Rifampin-fluconazole interaction in critically ill patients. Ann Pharmacother.

[154] Iatsimirskaia E, Tulebaev S, Storozhuk E, Utkin I, Smith D, Gerber N (1997). Metabolism of rifabutin in human enterocyte and liver microsomes: kinetic parameters, identification of enzyme systems, and drug interactions with macrolides and antifungal agents. Clin Pharmacol Ther.

[155] De Bellis P, Bonfiglio M, Gerbi G, Bacigalupo P, Buscaglia G, Guido P (2003). High-dose fluconazole therapy in intensive care unit. Minerva Anestesiol..

[156] Sinnollareddy MG, Roberts MS, Lipman J, Lassig-Smith M, Starr T, Robertson T (2016). In vivo microdialysis to determine subcutaneous interstitial fluid penetration and pharmacokinetics of fluconazole in intensive care unit patients with sepsis. Antimicrob Agents Chemother.

[157] Sinnollareddy MG, Roberts JA, Lipman J, Akova M, Bassetti M, De Waele JJ (2015). Pharmacokinetic variability and exposures of fluconazole, anidulafungin, and caspofungin in intensive care unit patients: data from multinational defining antibiotic levels in intensive care unit (DALI) patients Study. Crit Care.

[158] Alobaid AS, Wallis SC, Jarrett P, Starr T, Stuart J, Lassig-Smith M (2016). Effect of obesity on the population pharmacokinetics of fluconazole in critically ill patients. Antimicrob Agents Chemother.

[159] Momper JD, Capparelli EV, Wade KC, Kantak A, Dhanireddy R, Cummings JJ (2016). Population pharmacokinetics of fluconazole in premature infants with birth weights less than 750 grams. Antimicrob Agents Chemother.

[160] Oono S, Tabei K, Tetsuka T, Asano Y (1992). The pharmacokinetics of fluconazole during haemodialysis in uraemic patients. Eur J Clin Pharmacol.

[161] Debruyne D, Ryckelynck JP (1992). Fluconazole serum, urine, and dialysate levels in CAPD patients. Perit Dial Int.

[162] Yagasaki K, Gando S, Matsuda N, Kameue T, Ishitani T, Hirano T (2003). Pharmacokinetics and the most suitable dosing regimen of fluconazole in critically ill patients receiving continuous hemodiafiltration. Intensive Care Med.

[163] Kishino S, Koshinami Y, Hosoi T, Suda N, Takekuma Y, Gandoh S (2001). Effective fluconazole therapy for liver transplant recipients during continuous hemodiafiltration. Ther Drug Monit.

[164] Gharibian KN, Mueller BA (2016). Fluconazole dosing predictions in critically-ill patients receiving prolonged intermittent renal replacement therapy: a Monte Carlo simulation approach. Clin Nephrol.

[165] Sinnollareddy MG, Roberts MS, Lipman J, Peake SL, Roberts JA (2015). Influence of sustained low-efficiency diafiltration (SLED-f) on interstitial fluid concentrations of fluconazole in a critically ill patient: use of microdialysis. Int J Antimicrob Agents.

[166] Watt KM, Gonzalez D, Benjamin DK, Brouwer KL, Wade KC, Capparelli E (2015). Fluconazole population pharmacokinetics and dosing for prevention and treatment of invasive Candidiasis in children supported with extracorporeal membrane oxygenation. Antimicrob Agents Chemother.

[167] Walsh TJ, Foulds G, Pizzo PA (1989). Pharmacokinetics and tissue penetration of fluconazole in rabbits. Antimicrob Agents Chemother.

[168] Yang H, Wang Q, Elmquist WF (1996). Fluconazole distribution to the brain: a crossover study in freely-moving rats using in vivo microdialysis. Pharm Res.

[169] Brammer KW, Farrow PR, Faulkner JK (1990). Pharmacokinetics and tissue penetration of fluconazole in humans. Rev Infect Dis.

[170] Foulds G, Brennan DR, Wajszczuk C, Catanzaro A, Garg DC, Knopf W (1988). Fluconazole penetration into cerebrospinal fluid in humans. J Clin Pharmacol.

[171] Ebden P, Neill P, Farrow PR (1989). Sputum levels of fluconazole in humans. Antimicrob Agents Chemother.

[172] Thaler F, Bernard B, Tod M, Jedynak CP, Petitjean O, Derome P (1995). Fluconazole penetration in cerebral parenchyma in humans at steady state. Antimicrob Agents Chemother.

[173] Pea F, Righi E, Cojutti P, Carnelutti A, Baccarani U, Soardo G (2014). Intra-abdominal penetration and pharmacodynamic exposure to fluconazole in three liver transplant patients with deep-seated candidiasis. J Antimicrob Chemother.

[174] Bozzette SA, Gordon RL, Yen A, Rinaldi M, Ito MK, Fierer J (1992). Biliary concentrations of fluconazole in a patient with candidal cholecystitis: case report. Clin Infect Dis.

[175] Ahmad SR, Singer SJ, Leissa BG (2001). Congestive heart failure associated with itraconazole. Lancet.

[176] Caputo R (2003). Itraconazole (Sporanox) in superficial and systemic fungal infections. Expert Rev Anti Infect Ther..

[177] Al-Nakeeb Z, Sudan A, Jeans AR, Gregson L, Goodwin J, Warn PA (2012). Pharmacodynamics of Itraconazole against *Aspergillus fumigatus* in an in vitro model of the human alveolus: perspectives on the treatment of triazole-resistant infection and utility of airway administration. Antimicrob Agents Chemother.

[178] Caillot D, Bassaris H, McGeer A, Arthur C, Prentice HG, Seifert W (2001). Intravenous itraconazole followed by oral itraconazole in the treatment of invasive pulmonary aspergillosis in patients with hematologic malignancies, chronic granulomatous disease, or AIDS. Clin Infect Dis.

[179] Denning DW, Lee JY, Hostetler JS, Pappas P, Kauffman CA, Dewsnup DH, NIAID Mycoses Study Group (1994). Multicenter trial of oral itraconazole therapy for invasive aspergillosis. Am J Med.

[180] Denning DW, Hope WW (2010). Therapy for fungal diseases: opportunities and priorities. Trends Microbiol.

[181] Denning DW, Cadranel J, Beigelman-Aubry C, Ader F, Chakrabarti A, Blot S (2016). Chronic pulmonary aspergillosis: rationale and clinical guidelines for diagnosis and management. Eur Respir J.

[182] Chapman SW, Dismukes WE, Proia LA, Bradsher RW, Pappas PG, Threlkeld MG (2008). Clinical practice guidelines for the management of blastomycosis: 2008 update by the Infectious Diseases Society of America. Clin Infect Dis.

[183] Wheat LJ, Freifeld AG, Kleiman MB, Baddley JW, McKinsey DS, Loyd JE (2007). Clinical practice guidelines for the management of patients with histoplasmosis: 2007 update by the Infectious Diseases Society of America. Clin Infect Dis.

[184] Galgiani JN, Ampel NM, Blair JE, Catanzaro A, Geertsma F, Hoover SE (2016). 2016 Infectious Diseases Society of America (IDSA) clinical practice guideline for the treatment of coccidioidomycosis. Clin Infect Dis.

[185] Heykants J, Van Peer A, Van de Velde V, Van Rooy P, Meuldermans W, Lavrijsen K (1989). The clinical pharmacokinetics of itraconazole: an overview. Mycoses.

[186] De Beule K, Van Gestel J (2001). Pharmacology of itraconazole. Drugs..

[187] Grant SM, Clissold SP (1989). Itraconazole. A review of its pharmacodynamic and pharmacokinetic properties, and therapeutic use in superficial and systemic mycoses. Drugs..

[188] Lim SG, Sawyerr AM, Hudson M, Sercombe J, Pounder RE (1993). Short report: the absorption of fluconazole and itraconazole under conditions of low intragastric acidity. Aliment Pharmacol Ther.

[189] Graybill JR, Vazquez J, Darouiche RO, Morhart R, Greenspan D, Tuazon C (1998). Randomized trial of itraconazole oral solution for oropharyngeal candidiasis in HIV/AIDS patients. Am J Med.

[190] Prentice AG, Warnock DW, Johnson SA, Phillips MJ, Oliver DA (1994). Multiple dose pharmacokinetics of an oral solution of itraconazole in autologous bone marrow transplant recipients. J Antimicrob Chemother.

[191] Willems L, van der Geest R, de Beule K (2001). Itraconazole oral solution and intravenous formulations: a review of pharmacokinetics and pharmacodynamics. J Clin Pharm Ther.

[192] Barone JA, Moskovitz BL, Guarnieri J, Hassell AE, Colaizzi JL, Bierman RH (1998). Enhanced bioavailability of itraconazole in hydroxypropyl-beta-cyclodextrin solution versus capsules in healthy volunteers. Antimicrob Agents Chemother.

[193] Poirier JM, Cheymol G (1998). Optimisation of itraconazole therapy using target drug concentrations. Clin Pharmacokinet.

[194] Haria M, Bryson HM, Goa KL (1996). Itraconazole. A reappraisal of its pharmacological properties and therapeutic use in the management of superficial fungal infections. Drugs..

[195] Goodwin ML, Drew RH (2008). Antifungal serum concentration monitoring: an update. J Antimicrob Chemother.

[196] Kovarik JM, Hsu CH, McMahon L, Berthier S, Rordorf C (2001). Population pharmacokinetics of everolimus in de novo renal transplant patients: impact of ethnicity and comedications. Clin Pharmacol Ther.

[197] Jalava KM, Olkkola KT, Neuvonen PJ (1997). Itraconazole greatly increases plasma concentrations and effects of felodipine. Clin Pharmacol Ther.

[198] Neuvonen PJ, Suhonen R (1995). Itraconazole interacts with felodipine. J Am Acad Dermatol.

[199] Tailor SA, Gupta AK, Walker SE, Shear NH (1996). Peripheral edema due to nifedipine-itraconazole interaction: a case report. Arch Dermatol.

[200] Lempers VJ, van den Heuvel JJ, Russel FG, Aarnoutse RE, Burger DM, Brüggemann RJ (2016). Inhibitory potential of antifungal drugs on ATP-binding cassette transporters P-glycoprotein, MRP1 to MRP5, BCRP, and BSEP. Antimicrob Agents Chemother.

[201] Vandewoude K, Vogelaers D, Decruyenaere J, Jaqmin P, De Beule K, Van Peer A (1997). Concentrations in plasma and safety of 7 days of intravenous itraconazole followed by 2 weeks of oral itraconazole solution in patients in intensive care units. Antimicrob Agents Chemother.

[202] Coronel B, Persat F, Dorez D, Moskovtchenko JF, Peins MA, Mercatello A (1994). Itraconazole concentrations during continuous haemodiafiltration. J Antimicrob Chemother.

[203] Perfect JR, Savani DV, Durack DT (1993). Uptake of itraconazole by alveolar macrophages. Antimicrob Agents Chemother.

[204] Herbrecht R, Denning DW, Patterson TF, Bennett JE, Greene RE, Oestmann JW (2002). Voriconazole versus amphotericin B for primary therapy of invasive aspergillosis. N Engl J Med.

[205] Walsh TJ, Anaissie EJ, Denning DW, Herbrecht R, Kontoyiannis DP, Marr KA (2008). Treatment of aspergillosis: clinical practice guidelines of the Infectious Diseases Society of America. Clin Infect Dis.

[206] Chryssanthou E, Loebig A, Sjölin J (2008). Post-antifungal effect of amphotericin B and voriconazole against germinated Aspergillus fumigatus conidia. J Antimicrob Chemother.

[207] Jeans AR, Howard SJ, Al-Nakeeb Z, Goodwin J, Gregson L, Majithiya JB (2012). Pharmacodynamics of voriconazole in a dynamic in vitro model of invasive pulmonary aspergillosis: implications for in vitro susceptibility breakpoints. J Infect Dis.

[208] Jeu L, Piacenti FJ, Lyakhovetskiy AG, Fung HB (2003). Voriconazole. Clin Ther.

[209] Roffey SJ, Cole S, Comby P, Gibson D, Jezequel SG, Nedderman AN (2003). The disposition of voriconazole in mouse, rat, rabbit, guinea pig, dog, and human. Drug Metab Dispos.

[210] Purkins L, Wood N, Ghahramani P, Greenhalgh K, Allen MJ, Kleinermans D (2002). Pharmacokinetics and safety of voriconazole following intravenous- to oral-dose escalation regimens. Antimicrob Agents Chemother.

[211] Donnelly JP, De Pauw BE (2004). Voriconazole-a new therapeutic agent with an extended spectrum of antifungal activity. Clin Microbiol Infect.

[212] Wood N, Tan K, Purkins L, Layton G, Hamlin J, Kleinermans D (2003). Effect of omeprazole on the steady-state pharmacokinetics of voriconazole. Br J Clin Pharmacol.

[213] Ullmann AJ (2003). Review of the safety, tolerability, and drug interactions of the new antifungal agents caspofungin and voriconazole. Curr Med Res Opin.

[214] Rengelshausen J, Banfield M, Riedel KD, Burhenne J, Weiss J, Thomsen T (2005). Opposite effects of short-term and long-term St John’s wort intake on voriconazole pharmacokinetics. Clin Pharmacol Ther.

[215] Han K, Capitano B, Bies R, Potoski BA, Husain S, Gilbert S (2010). Bioavailability and population pharmacokinetics of voriconazole in lung transplant recipients. Antimicrob Agents Chemother.

[216] Weiler S, Zoller H, Graziadei I, Vogel W, Bellmann-Weiler R, Joannidis M (2007). Altered pharmacokinetics of voriconazole in a patient with liver cirrhosis. Antimicrob Agents Chemother.

[217] Fuhrmann V, Schenk P, Jaeger W, Miksits M, Kneidinger N, Warszawska J (2007). Pharmacokinetics of voriconazole during continuous venovenous haemodiafiltration. J Antimicrob Chemother.

[218] Robatel C, Rusca M, Padoin C, Marchetti O, Liaudet L, Buclin T (2004). Disposition of voriconazole during continuous veno-venous haemodiafiltration (CVVHDF) in a single patient. J Antimicrob Chemother.

[219] Radej J, Krouzecky A, Stehlik P, Sykora R, Chvojka J, Karvunidis T (2011). Pharmacokinetic evaluation of voriconazole treatment in critically ill patients undergoing continuous venovenous hemofiltration. Ther Drug Monit.

[220] Kiser TH, Fish DN, Aquilante CL, Rower JE, Wempe MF, MacLaren R (2015). Evaluation of sulfobutylether-β-cyclodextrin (SBECD) accumulation and voriconazole pharmacokinetics in critically ill patients undergoing continuous renal replacement therapy. Crit Care.

[221] Quintard H, Papy E, Massias L, Lasocki S, Arnaud P, Desmonts JM (2008). The pharmacokinetic profile of voriconazole during continuous high-volume venovenous hemofiltration in a critically ill patient. Ther Drug Monit.

[222] von Mach MA, Burhenne J, Weilemann LS (2006). Accumulation of the solvent vehicle sulphobutylether beta cyclodextrin sodium in critically ill patients treated with intravenous voriconazole under renal replacement therapy. BMC Clin Pharmacol.

[223] Hafner V, Czock D, Burhenne J, Riedel KD, Bommer J, Mikus G (2010). Pharmacokinetics of sulfobutylether-beta-cyclodextrin and voriconazole in patients with end-stage renal failure during treatment with two hemodialysis systems and hemodiafiltration. Antimicrob Agents Chemother.

[224] Spriet I, Annaert P, Meersseman P, Hermans G, Meersseman W, Verbesselt R (2009). Pharmacokinetics of caspofungin and voriconazole in critically ill patients during extracorporeal membrane oxygenation. J Antimicrob Chemother.

[225] Pascual A, Calandra T, Bolay S, Buclin T, Bille J, Marchetti O (2008). Voriconazole therapeutic drug monitoring in patients with invasive mycoses improves efficacy and safety outcomes. Clin Infect Dis.

[226] Lutsar I, Roffey S, Troke P (2003). Voriconazole concentrations in the cerebrospinal fluid and brain tissue of guinea pigs and immunocompromised patients. Clin Infect Dis.

[227] Weiler S, Fiegl D, MacFarland R, Stienecke E, Bellmann-Weiler R, Dunzendorfer S (2011). Human tissue distribution of voriconazole. Antimicrob Agents Chemother.

[228] Denes E, Pichon N, Debette-Gratien M, Bouteille B, Gaulier JM (2004). Pharmacokinetics of voriconazole in the cerebrospinal fluid of an immunocompromised patient with a brain abscess due to *Aspergillus fumigatus*. Clin Infect Dis.

[229] Crandon JL, Banevicius MA, Fang AF, Crownover PH, Knauft RF, Pope JS (2009). Bronchopulmonary disposition of intravenous voriconazole and anidulafungin given in combination to healthy adults. Antimicrob Agents Chemother.

[230] Capitano B, Potoski BA, Husain S, Zhang S, Paterson DL, Studer SM (2006). Intrapulmonary penetration of voriconazole in patients receiving an oral prophylactic regimen. Antimicrob Agents Chemother.

[231] Stern JB, Girard P, Caliandro R (2004). Pleural diffusion of voriconazole in a patient with Aspergillus fumigatus empyema thoracis. Antimicrob Agents Chemother.

[232] Howard SJ, Lestner JM, Sharp A, Gregson L, Goodwin J, Slater J (2011). Pharmacokinetics and pharmacodynamics of posaconazole for invasive pulmonary aspergillosis: clinical implications for antifungal therapy. J Infect Dis.

[233] Mavridou E, Brüggemann RJ, Melchers WJ, Mouton JW, Verweij PE (2010). Efficacy of posaconazole against three clinical *Aspergillus fumigatus* isolates with mutations in the cyp51A gene. Antimicrob Agents Chemother.

[234] Ullmann AJ, Lipton JH, Vesole DH, Chandrasekar P, Langston A, Tarantolo SR (2007). Posaconazole or fluconazole for prophylaxis in severe graft-versus-host disease. N Engl J Med.

[235] Cornely OA, Maertens J, Winston DJ, Perfect J, Ullmann AJ, Walsh TJ (2007). Posaconazole vs. fluconazole or itraconazole prophylaxis in patients with neutropenia. N Engl J Med..

[236] Walsh TJ, Raad I, Patterson TF, Chandrasekar P, Donowitz GR, Graybill R (2007). Treatment of invasive aspergillosis with posaconazole in patients who are refractory to or intolerant of conventional therapy: an externally controlled trial. Clin Infect Dis.

[237] Herbrecht R (2004). Posaconazole: a potent, extended-spectrum triazole anti-fungal for the treatment of serious fungal infections. Int J Clin Pract.

[238] Courtney R, Wexler D, Radwanski E, Lim J, Laughlin M (2004). Effect of food on the relative bioavailability of two oral formulations of posaconazole in healthy adults. Br J Clin Pharmacol.

[239] Courtney R, Pai S, Laughlin M, Lim J, Batra V (2003). Pharmacokinetics, safety, and tolerability of oral posaconazole administered in single and multiple doses in healthy adults. Antimicrob Agents Chemother.

[240] Nomeir AA, Kumari P, Hilbert MJ, Gupta S, Loebenberg D, Cacciapuoti A (2000). Pharmacokinetics of SCH 56592, a new azole broad-spectrum antifungal agent, in mice, rats, rabbits, dogs, and cynomolgus monkeys. Antimicrob Agents Chemother.

[241] Courtney R, Sansone A, Smith W, Marbury T, Statkevich P, Martinho M (2005). Posaconazole pharmacokinetics, safety, and tolerability in subjects with varying degrees of chronic renal disease. J Clin Pharmacol.

[242] Ezzet F, Wexler D, Courtney R, Krishna G, Lim J, Laughlin M (2005). Oral bioavailability of posaconazole in fasted healthy subjects: comparison between three regimens and basis for clinical dosage recommendations. Clin Pharmacokinet.

[243] Krishna G, Ma L, Martinho M, O’Mara E (2012). Single-dose phase I study to evaluate the pharmacokinetics of posaconazole in new tablet and capsule formulations relative to oral suspension. Antimicrob Agents Chemother.

[244] Kraft WK, Chang PS, van Iersel ML, Waskin H, Krishna G, Kersemaekers WM (2014). Posaconazole tablet pharmacokinetics: lack of effect of concomitant medications altering gastric pH and gastric motility in healthy subjects. Antimicrob Agents Chemother.

[245] Krishna G, Ma L, Martinho M, Preston RA, O’Mara E (2012). A new solid oral tablet formulation of posaconazole: a randomized clinical trial to investigate rising single- and multiple-dose pharmacokinetics and safety in healthy volunteers. J Antimicrob Chemother.

[246] Cornely OA, Duarte RF, Haider S, Chandrasekar P, Helfgott D, Jiménez JL (2016). Phase 3 pharmacokinetics and safety study of a posaconazole tablet formulation in patients at risk for invasive fungal disease. J Antimicrob Chemother.

[247] Kersemaekers WM, Dogterom P, Xu J, Marcantonio EE, de Greef R, Waskin H (2015). Effect of a high-fat meal on the pharmacokinetics of 300-milligram posaconazole in a solid oral tablet formulation. Antimicrob Agents Chemother.

[248] Maertens J, Cornely OA, Ullmann AJ, Heinz WJ, Krishna G, Patino H (2014). Phase 1B study of the pharmacokinetics and safety of posaconazole intravenous solution in patients at risk for invasive fungal disease. Antimicrob Agents Chemother.

[249] Wexler D, Courtney R, Richards W, Banfield C, Lim J, Laughlin M (2004). Effect of posaconazole on cytochrome P450 enzymes: a randomized, open-label, two-way crossover study. Eur J Pharm Sci.

[250] Petitcollin A, Crochette R, Tron C, Verdier MC, Boglione-Kerrien C, Vigneau C (2016). Increased inhibition of cytochrome P450 3A4 with the tablet formulation of posaconazole. Drug Metab Pharmacokinet.

[251] Groll AH, Walsh TJ (2005). Posaconazole: clinical pharmacology and potential for management of fungal infections. Expert Rev Anti Infect Ther..

[252] Sandherr M, Maschmeyer G (2011). Pharmacology and metabolism of voriconazole and posaconazole in the treatment of invasive aspergillosis: review of the literature. Eur J Med Res..

[253] Howard SJ, Felton TW, Gomez-Lopez A, Hope WW (2012). Posaconazole: the case for therapeutic drug monitoring. Ther Drug Monit.

[254] Weiler S, Lass-Flörl C, Auberger J, Bellmann-Weiler R, Stein M, Joannidis M (2009). Triazole-resistant candidaemia following posaconazole exposure. Int J Antimicrob Agents.

[255] Arendrup MC, Cuenca-Estrella M, Lass-Flörl C, Hope WW (2012). (EUCAST-AFST) ECoASTSoAST. EUCAST technical note on Aspergillus and amphotericin B, itraconazole, and posaconazole. Clin Microbiol Infect.

[256] Dekkers BG, Bakker M, van der Elst KC, Sturkenboom MG, Veringa A, Span LF (2016). Therapeutic drug monitoring of posaconazole: an update. Curr Fungal Infect Rep..

[257] Ray J, Campbell L, Rudham S, Nguyen Q, Marriott D (2011). Posaconazole plasma concentrations in critically ill patients. Ther Drug Monit.

[258] Morris AA, Mueller SW, Rower JE, Washburn T, Kiser TH (2015). Evaluation of sulfobutylether-β-cyclodextrin exposure in a critically ill patient receiving intravenous posaconazole while undergoing continuous venovenous hemofiltration. Antimicrob Agents Chemother.

[259] Conte JE, Golden JA, Krishna G, McIver M, Little E, Zurlinden E (2009). Intrapulmonary pharmacokinetics and pharmacodynamics of posaconazole at steady state in healthy subjects. Antimicrob Agents Chemother.

[260] Conte JE, DeVoe C, Little E, Golden JA (2010). Steady-state intrapulmonary pharmacokinetics and pharmacodynamics of posaconazole in lung transplant recipients. Antimicrob Agents Chemother.

[261] Thakuria L, Packwood K, Firouzi A, Rogers P, Soresi S, Habibi-Parker K (2016). A pharmacokinetic analysis of posaconazole oral suspension in the serum and alveolar compartment of lung transplant recipients. Int J Antimicrob Agents.

[262] Blennow O, Eliasson E, Pettersson T, Pohanka A, Szakos A, El-Serafi I (2014). Posaconazole concentrations in human tissues after allogeneic stem cell transplantation. Antimicrob Agents Chemother.

[263] Cendejas-Bueno E, Forastiero A, Ruiz I, Mellado E, Gavaldà J, Gomez-Lopez A (2017). Blood and tissue distribution of posaconazole in a rat model of invasive pulmonary aspergillosis. Diagn Microbiol Infect Dis.

[264] Reinwald M, Uharek L, Lampe D, Grobosch T, Thiel E, Schwartz S (2009). Limited penetration of posaconazole into cerebrospinal fluid in an allogeneic stem cell recipient with invasive pulmonary aspergillosis. Bone Marrow Transplant.

[265] Calcagno A, Baietto L, De Rosa FG, Tettoni MC, Libanore V, Bertucci R (2011). Posaconazole cerebrospinal concentrations in an HIV-infected patient with brain mucormycosis. J Antimicrob Chemother.

[266] Rüping MJ, Albermann N, Ebinger F, Burckhardt I, Beisel C, Müller C (2008). Posaconazole concentrations in the central nervous system. J Antimicrob Chemother.

[267] Ressaire Q, Padoin C, Chaouat M, Maurel V, Alanio A, Ferry A (2015). Muscle diffusion of liposomal amphotericin B and posaconazole in critically ill burn patients receiving continuous hemodialysis. Intensive Care Med.

[268] Campoli P, Al Abdallah Q, Robitaille R, Solis NV, Fielhaber JA, Kristof AS (2011). Concentration of antifungal agents within host cell membranes: a new paradigm governing the efficacy of prophylaxis. Antimicrob Agents Chemother.

[269] Campoli P, Perlin DS, Kristof AS, White TC, Filler SG, Sheppard DC (2013). Pharmacokinetics of posaconazole within epithelial cells and fungi: insights into potential mechanisms of action during treatment and prophylaxis. J Infect Dis.

[270] Pasqualotto AC, Denning DW (2008). New and emerging treatments for fungal infections. J Antimicrob Chemother.

[271] Murrell D, Bossaer JB, Carico R, Harirforoosh S, Cluck D (2016). Isavuconazonium sulfate: a triazole prodrug for invasive fungal infections. Int J Pharm Pract..

[272] Maertens JA, Raad II, Marr KA, Patterson TF, Kontoyiannis DP, Cornely OA (2016). Isavuconazole versus voriconazole for primary treatment of invasive mould disease caused by Aspergillus and other filamentous fungi (SECURE): a phase 3, randomised-controlled, non-inferiority trial. Lancet.

[273] Keirns J, Desai A, Kowalski D, Lademacher C, Mujais S, Parker B (2017). QT interval shortening with isavuconazole: in vitro and in vivo effects on cardiac repolarization. Clin Pharmacol Ther.

[274] Schmitt-Hoffmann A, Roos B, Maares J, Heep M, Spickerman J, Weidekamm E (2006). Multiple-dose pharmacokinetics and safety of the new antifungal triazole BAL4815 after intravenous infusion and oral administration of its prodrug, BAL8557, in healthy volunteers. Antimicrob Agents Chemother.

[275] Schmitt-Hoffmann A, Roos B, Heep M, Schleimer M, Weidekamm E, Brown T (2006). Single-ascending-dose pharmacokinetics and safety of the novel broad-spectrum antifungal triazole BAL4815 after intravenous infusions (50, 100, and 200 milligrams) and oral administrations (100, 200, and 400 milligrams) of its prodrug, BAL8557, in healthy volunteers. Antimicrob Agents Chemother.

[276] Desai A, Kovanda L, Kowalski D, Lu Q, Townsend R, Bonate PL (2016). Population pharmacokinetics of isavuconazole from phase 1 and phase 3 (SECURE) trials in adults and target attainment in patients with invasive infections due to aspergillus and other filamentous fungi. Antimicrob Agents Chemother.

[277] Kovanda LL, Desai AV, Lu Q, Townsend RW, Akhtar S, Bonate P (2016). Isavuconazole population pharmacokinetic analysis using nonparametric estimation in patients with invasive fungal disease (results from the VITAL study). Antimicrob Agents Chemother.

[278] Groll AH, Desai A, Han D, Howieson C, Kato K, Akhtar S (2016). Pharmacokinetic assessment of drug–drug interactions of isavuconazole with the immunosuppressants cyclosporine, mycophenolic acid, prednisolone, sirolimus, and tacrolimus in healthy adults. Clin Pharmacol Drug Dev.

[279] Miceli MH, Kauffman CA (2015). Isavuconazole: a new broad-spectrum triazole antifungal agent. Clin Infect Dis.

[280] Desai A, Yamazaki T, Dietz AJ, Kowalski D, Lademacher C, Pearlman H (2016). Pharmacokinetic and pharmacodynamic evaluation of the drug–drug interaction between isavuconazole and warfarin in healthy subjects. Clin Pharmacol Drug Dev..

[281] Townsend RW, Akhtar S, Alcorn H, Berg JK, Kowalski DL, Mujais S (2017). Phase I trial to investigate the effect of renal impairment on isavuconazole pharmacokinetics. Eur J Clin Pharmacol.

[282] Schmitt-Hoffmann A, Roos B, Spickermann J, Heep M, Peterfai E, Edwards DJ (2009). Effect of mild and moderate liver disease on the pharmacokinetics of isavuconazole after intravenous and oral administration of a single dose of the prodrug BAL8557. Antimicrob Agents Chemother.

[283] Desai A, Schmitt-Hoffmann AH, Mujais S, Townsend R (2016). Population pharmacokinetics of isavuconazole in subjects with mild or moderate hepatic impairment. Antimicrob Agents Chemother.

[284] Knoll BM (2014). Pharmacokinetics of oral isavuconazole in a patient after Roux-en-Y gastric bypass surgery. J Antimicrob Chemother.

[285] Ervens J, Ghannoum M, Graf B, Schwartz S (2014). Successful isavuconazole salvage therapy in a patient with invasive mucormycosis. Infection.

[286] Wiederhold NP, Kovanda L, Najvar LK, Bocanegra R, Olivo M, Kirkpatrick WR (2016). Isavuconazole is effective for the treatment of experimental cryptococcal meningitis. Antimicrob Agents Chemother.

[287] Chen SC, Slavin MA, Sorrell TC (2011). Echinocandin antifungal drugs in fungal infections: a comparison. Drugs..

[288] Cappelletty D, Eiselstein-McKitrick K (2007). The echinocandins. Pharmacotherapy..

[289] Denning DW (2003). Echinocandin antifungal drugs. Lancet.

[290] Bowman JC, Hicks PS, Kurtz MB, Rosen H, Schmatz DM, Liberator PA (2002). The antifungal echinocandin caspofungin acetate kills growing cells of *Aspergillus fumigatus* in vitro. Antimicrob Agents Chemother.

[291] Vanstraelen K, Lagrou K, Maertens J, Wauters J, Willems L, Spriet I (2013). The Eagle-like effect of echinocandins: what’s in a name?. Expert Rev Anti Infect Ther..

[292] Wiederhold NP (2007). Attenuation of echinocandin activity at elevated concentrations: a review of the paradoxical effect. Curr Opin Infect Dis..

[293] Lewis RE, Albert ND, Kontoyiannis DP (2008). Comparison of the dose-dependent activity and paradoxical effect of caspofungin and micafungin in a neutropenic murine model of invasive pulmonary aspergillosis. J Antimicrob Chemother.

[294] Tsai D, Lipman J, Roberts JA (2015). Pharmacokinetic/pharmacodynamic considerations for the optimization of antimicrobial delivery in the critically ill. Curr Opin Crit Care..

[295] Andes DR, Reynolds DK, Van Wart SA, Lepak AJ, Kovanda LL, Bhavnani SM (2013). Clinical pharmacodynamic index identification for micafungin in esophageal candidiasis: dosing strategy optimization. Antimicrob Agents Chemother.

[296] Arendrup MC, Perlin DS, Jensen RH, Howard SJ, Goodwin J, Hope W (2012). Differential in vivo activities of anidulafungin, caspofungin, and micafungin against *Candida glabrata* isolates with and without FKS resistance mutations. Antimicrob Agents Chemother.

[297] Pea F (2013). Current pharmacological concepts for wise use of echinocandins in the treatment of *Candida* infections in septic critically ill patients. Expert Rev Anti Infect Ther..

[298] Lepak A, Castanheira M, Diekema D, Pfaller M, Andes D (2012). Optimizing Echinocandin dosing and susceptibility breakpoint determination via in vivo pharmacodynamic evaluation against *Candida glabrata* with and without fks mutations. Antimicrob Agents Chemother.

[299] Andes DR, Diekema DJ, Pfaller MA, Marchillo K, Bohrmueller J (2008). In vivo pharmacodynamic target investigation for micafungin against *Candida albicans* and C. glabrata in a neutropenic murine candidiasis model. Antimicrob Agents Chemother.

[300] Pappas PG, Kauffman CA, Andes D, Benjamin DK, Calandra TF, Edwards JE (2009). Clinical practice guidelines for the management of candidiasis: 2009 update by the Infectious Diseases Society of America. Clin Infect Dis.

[301] Cornely OA, Bassetti M, Calandra T, Garbino J, Kullberg BJ, Lortholary O (2012). ESCMID* guideline for the diagnosis and management of *Candida* diseases 2012: non-neutropenic adult patients. Clin Microbiol Infect.

[302] Ullmann AJ, Akova M, Herbrecht R, Viscoli C, Arendrup MC, Arikan-Akdagli S (2012). ESCMID* guideline for the diagnosis and management of *Candida* diseases 2012: adults with haematological malignancies and after haematopoietic stem cell transplantation (HCT). Clin Microbiol Infect.

[303] European Medicines Agency, Ecalta. EPAR-Product Information. 2014. http://www.ema.europa.eu. Accessed 3 Mar 2017.

[304] European Medicines Agency, Mycamine. EPAR-Product Information. 2011. http://www.ema.europa.eu. Accessed 3 Mar 2017.

[305] European Medicines Agency, Cancidas. EPAR-Product Information. 2016. http://www.ema.europa.eu. Accessed 3 Mar 2017.

[306] Wiederhold NP, Lewis RE (2003). The echinocandin antifungals: an overview of the pharmacology, spectrum and clinical efficacy. Expert Opin Investig Drugs.

[307] Mora-Duarte J, Betts R, Rotstein C, Colombo AL, Thompson-Moya L, Smietana J (2002). Comparison of caspofungin and amphotericin B for invasive candidiasis. N Engl J Med.

[308] Ullmann AJ, Akova M, Herbrecht R, Viscoli C, Arendrup MC, Arikan-Akdagli S (2012). ESCMID* guideline for the diagnosis and management of *Candida* diseases 2012: adults with haematological malignancies and after haematopoietic stem cell transplantation (HCT). Clin Microbiol Infect.

[309] Freifeld AG, Bow EJ, Sepkowitz KA, Boeckh MJ, Ito JI, Mullen CA (2011). Clinical practice guideline for the use of antimicrobial agents in neutropenic patients with cancer: 2010 update by the infectious diseases society of america. Clin Infect Dis.

[310] Maertens J, Raad I, Petrikkos G, Boogaerts M, Selleslag D, Petersen FB (2004). Efficacy and safety of caspofungin for treatment of invasive aspergillosis in patients refractory to or intolerant of conventional antifungal therapy. Clin Infect Dis.

[311] Viscoli C, Herbrecht R, Akan H, Baila L, Sonet A, Gallamini A (2009). An EORTC phase II study of caspofungin as first-line therapy of invasive aspergillosis in haematological patients. J Antimicrob Chemother.

[312] Herbrecht R, Maertens J, Baila L, Aoun M, Heinz W, Martino R (2010). Caspofungin first-line therapy for invasive aspergillosis in allogeneic hematopoietic stem cell transplant patients: an European Organisation for Research and Treatment of Cancer study. Bone Marrow Transplant.

[313] Stone JA, Xu X, Winchell GA, Deutsch PJ, Pearson PG, Migoya EM (2004). Disposition of caspofungin: role of distribution in determining pharmacokinetics in plasma. Antimicrob Agents Chemother.

[314] Stone JA, Holland SD, Wickersham PJ, Sterrett A, Schwartz M, Bonfiglio C (2002). Single- and multiple-dose pharmacokinetics of caspofungin in healthy men. Antimicrob Agents Chemother.

[315] Cornely OA, Vehreschild JJ, Vehreschild MJ, Würthwein G, Arenz D, Schwartz S (2011). Phase II dose escalation study of caspofungin for invasive Aspergillosis. Antimicrob Agents Chemother.

[316] Würthwein G, Cornely OA, Trame MN, Vehreschild JJ, Vehreschild MJ, Farowski F (2013). Population pharmacokinetics of escalating doses of caspofungin in a phase II study of patients with invasive aspergillosis. Antimicrob Agents Chemother.

[317] Balani SK, Xu X, Arison BH, Silva MV, Gries A, DeLuna FA (2000). Metabolites of caspofungin acetate, a potent antifungal agent, in human plasma and urine. Drug Metab Dispos.

[318] Johnson MD, Perfect JR (2003). Caspofungin: first approved agent in a new class of antifungals. Expert Opin Pharmacother.

[319] Würthwein G, Young C, Lanvers-Kaminsky C, Hempel G, Trame MN, Schwerdtfeger R (2012). Population pharmacokinetics of liposomal amphotericin B and caspofungin in allogeneic hematopoietic stem cell recipients. Antimicrob Agents Chemother.

[320] Mistry GC, Migoya E, Deutsch PJ, Winchell G, Hesney M, Li S (2007). Single- and multiple-dose administration of caspofungin in patients with hepatic insufficiency: implications for safety and dosing recommendations. J Clin Pharmacol.

[321] Martial LC, Brüggemann RJ, Schouten JA, van Leeuwen HJ, van Zanten AR, de Lange DW (2016). dose reduction of caspofungin in intensive care unit patients with Child Pugh B will result in suboptimal exposure. Clin Pharmacokinet.

[322] Spriet I, Meersseman W, Annaert P, de Hoon J, Willems L (2011). Pharmacokinetics of caspofungin in a critically ill patient with liver cirrhosis. Eur J Clin Pharmacol.

[323] Nguyen TH, Hoppe-Tichy T, Geiss HK, Rastall AC, Swoboda S, Schmidt J (2007). Factors influencing caspofungin plasma concentrations in patients of a surgical intensive care unit. J Antimicrob Chemother.

[324] van der Elst KC, Veringa A, Zijlstra JG, Beishuizen A, Klont R, Brummelhuis-Visser P et al. Low caspofungin exposure in patients in intensive care units. Antimicrob Agents Chemother. 2017;61:e01582–e01616. 10.1128/AAC.01582-16 PMC5278683 27855112

[325] Muilwijk EW, Schouten JA, van Leeuwen HJ, van Zanten AR, de Lange DW, Colbers A (2014). Pharmacokinetics of caspofungin in ICU patients. J Antimicrob Chemother.

[326] Stone EA, Fung HB, Kirschenbaum HL (2002). Caspofungin: an echinocandin antifungal agent. Clin Ther.

[327] Weiler S, Seger C, Pfisterer H, Stienecke E, Stippler F, Welte R (2013). Pharmacokinetics of caspofungin in critically ill patients on continuous renal replacement therapy. Antimicrob Agents Chemother.

[328] Roger C, Wallis SC, Muller L, Saissi G, Lipman J, Brüggemann RJ (2016). Caspofungin Population Pharmacokinetics in Critically Ill Patients Undergoing Continuous Veno-Venous Haemofiltration or Haemodiafiltration. Clin Pharmacokinet..

[329] Goicoechea M, Fierer J, Johns S (2004). Treatment of candidal cholangitis with caspofungin therapy in a patient with a liver transplant: documentation of biliary excretion of caspofungin. Clin Infect Dis.

[330] Brüggemann RJ, Van Der Velden WJ, Knibbe CA, Colbers A, Hol S, Burger DM (2015). A rationale for reduced-frequency dosing of anidulafungin for antifungal prophylaxis in immunocompromised patients. J Antimicrob Chemother.

[331] Dowell JA, Knebel W, Ludden T, Stogniew M, Krause D, Henkel T (2004). Population pharmacokinetic analysis of anidulafungin, an echinocandin antifungal. J Clin Pharmacol.

[332] Damle BD, Dowell JA, Walsky RL, Weber GL, Stogniew M, Inskeep PB (2009). In vitro and in vivo studies to characterize the clearance mechanism and potential cytochrome P450 interactions of anidulafungin. Antimicrob Agents Chemother.

[333] Dowell JA, Schranz J, Baruch A, Foster G (2005). Safety and pharmacokinetics of coadministered voriconazole and anidulafungin. J Clin Pharmacol.

[334] Dowell JA, Stogniew M, Krause D, Henkel T, Weston IE (2005). Assessment of the safety and pharmacokinetics of anidulafungin when administered with cyclosporine. J Clin Pharmacol.

[335] Dowell JA, Stogniew M, Krause D, Henkel T, Damle B (2007). Lack of pharmacokinetic interaction between anidulafungin and tacrolimus. J Clin Pharmacol.

[336] Dowell JA, Stogniew M, Krause D, Damle B (2007). Anidulafungin does not require dosage adjustment in subjects with varying degrees of hepatic or renal impairment. J Clin Pharmacol.

[337] Lempers VJ, van Rongen A, van Dongen EP, van Ramshorst B, Burger DM, Aarnoutse RE (2016). Does weight impact anidulafungin pharmacokinetics?. Clin Pharmacokinet.

[338] Liu P, Ruhnke M, Meersseman W, Paiva JA, Kantecki M, Damle B (2013). Pharmacokinetics of anidulafungin in critically ill patients with candidemia/invasive candidiasis. Antimicrob Agents Chemother.

[339] Liu P, Mould DR (2014). Population pharmacokinetic–pharmacodynamic analysis of voriconazole and anidulafungin in adult patients with invasive aspergillosis. Antimicrob Agents Chemother.

[340] Brüggemann RJ, Middel-Baars V, de Lange DW, Colbers A, Girbes AR, Pickkers P (2017). Pharmacokinetics of anidulafungin in critically ill intensive care unit patients with suspected or proven invasive fungal infections. Antimicrob Agents Chemother.

[341] Aguilar G, Azanza JR, Carbonell JA, Ferrando C, Badenes R, Parra MA (2014). Anidulafungin dosing in critically ill patients with continuous venovenous haemodiafiltration. J Antimicrob Chemother.

[342] De Rosa FG, Corcione S, Baietto L, Pasero D, Di Perri G, Ranieri VM (2013). Pharmacokinetics of anidulafungin in two critically ill patients with septic shock undergoing CVVH. J Chemother.

[343] Leitner JM, Meyer B, Fuhrmann V, Saria K, Zuba C, Jäger W (2011). Multiple-dose pharmacokinetics of anidulafungin during continuous venovenous haemofiltration. J Antimicrob Chemother.

[344] Aguilar G, Azanza JR, Sádaba B, Badenes R, Ferrando C, Delgado C (2014). Pharmacokinetics of anidulafungin during albumin dialysis. Crit Care.

[345] Aguilar G, Ferriols R, Carbonell JA, Ezquer C, Alonso JM, Villena A (2016). Pharmacokinetics of anidulafungin during venovenous extracorporeal membrane oxygenation. Crit Care.

[346] Groll AH, Mickiene D, Petraitiene R, Petraitis V, Lyman CA, Bacher JS (2001). Pharmacokinetic and pharmacodynamic modeling of anidulafungin (LY303366): reappraisal of its efficacy in neutropenic animal models of opportunistic mycoses using optimal plasma sampling. Antimicrob Agents Chemother.

[347] Damle B, Stogniew M, Dowell J (2008). Pharmacokinetics and tissue distribution of anidulafungin in rats. Antimicrob Agents Chemother.

[348] Farowski F, Cornely OA, Vehreschild JJ, Wiesen M, Steinbach A, Vehreschild MJ (2013). Intracellular concentrations of anidulafungin in different compartments of the peripheral blood. Int J Antimicrob Agents.

[349] Hiemenz J, Cagnoni P, Simpson D, Devine S, Chao N, Keirns J (2005). Pharmacokinetic and maximum tolerated dose study of micafungin in combination with fluconazole versus fluconazole alone for prophylaxis of fungal infections in adult patients undergoing a bone marrow or peripheral stem cell transplant. Antimicrob Agents Chemother.

[350] Andes D, Ambrose PG, Hammel JP, Van Wart SA, Iyer V, Reynolds DK (2011). Use of pharmacokinetic-pharmacodynamic analyses to optimize therapy with the systemic antifungal micafungin for invasive candidiasis or candidemia. Antimicrob Agents Chemother.

[351] Grau S, Luque S, Campillo N, Samso E, Rodriguez U, Garcia-Bernedo CA (2015). Plasma and peritoneal fluid population pharmacokinetics of micafungin in post-surgical patients with severe peritonitis. J Antimicrob Chemother.

[352] Niwa T, Shiraga T, Takagi A (2005). Effect of antifungal drugs on cytochrome P450 (CYP) 2C9, CYP2C19, and CYP3A4 activities in human liver microsomes. Biol Pharm Bull.

[353] Hebert MF, Townsend RW, Austin S, Balan G, Blough DK, Buell D (2005). Concomitant cyclosporine and micafungin pharmacokinetics in healthy volunteers. J Clin Pharmacol.

[354] Hebert MF, Blough DK, Townsend RW, Allison M, Buell D, Keirns J (2005). Concomitant tacrolimus and micafungin pharmacokinetics in healthy volunteers. J Clin Pharmacol.

[355] Jarvis B, Figgitt DP, Scott LJ (2004). Micafungin. Drugs..

[356] Higashiyama Y, Kohno S (2004). Micafungin: a therapeutic review. Expert Rev Anti Infect Ther..

[357] Inoue Y, Saito T, Ogawa K, Nishio Y, Kosugi S, Suzuki Y (2012). Drug interactions between micafungin at high doses and cyclosporine A in febrile neutropenia patients after allogeneic hematopoietic stem cell transplantation. Int J Clin Pharmacol Ther.

[358] Undre NA, Stevenson P, Wilbraham D (2014). Pharmacokinetic profile of micafungin when co-administered with amphotericin B in healthy male subjects. Int J Clin Pharmacol Ther.

[359] Seibel NL, Schwartz C, Arrieta A, Flynn P, Shad A, Albano E (2005). Safety, tolerability, and pharmacokinetics of Micafungin (FK463) in febrile neutropenic pediatric patients. Antimicrob Agents Chemother.

[360] Undre NA, Stevenson P, Freire A, Arrieta A (2012). Pharmacokinetics of micafungin in pediatric patients with invasive candidiasis and candidemia. Pediatr Infect Dis J..

[361] Hope WW, Kaibara A, Roy M, Arrieta A, Azie N, Kovanda LL (2015). Population pharmacokinetics of micafungin and its metabolites M1 and M5 in children and adolescents. Antimicrob Agents Chemother.

[362] Yanni SB, Smith PB, Benjamin DK, Augustijns PF, Thakker, Annaert PP (2011). Higher clearance of micafungin in neonates compared with adults: role of age-dependent micafungin serum binding. Biopharm Drug Dispos.

[363] Hebert MF, Smith HE, Marbury TC, Swan SK, Smith WB, Townsend RW (2005). Pharmacokinetics of micafungin in healthy volunteers, volunteers with moderate liver disease, and volunteers with renal dysfunction. J Clin Pharmacol.

[364] Undre N, Pretorius B, Stevenson P (2015). Pharmacokinetics of micafungin in subjects with severe hepatic dysfunction. Eur J Drug Metab Pharmacokinet.

[365] Luque S, Campillo N, Álvarez-Lerma F, Ferrández O, Horcajada JP, Grau S (2016). Pharmacokinetics of micafungin in patients with pre-existing liver dysfunction: a safe option for treating invasive fungal infections. Enferm Infecc Microbiol Clin.

[366] Oshima K, Kanda Y, Kako S, Ohno K, Kishino S, Kurokawa M (2013). Pharmacokinetics of micafungin in patients undergoing allogeneic hematopoietic stem cell transplantation. Transpl Infect Dis..

[367] Kishino S, Ohno K, Shimamura T, Furukawatodo H (2004). Optimal prophylactic dosage and disposition of micafungin in living donor liver recipients. Clin Transplant.

[368] Hirata K, Aoyama T, Matsumoto Y, Ogawa F, Yamazaki H, Kikuti A (2007). Pharmacokinetics of antifungal agent micafungin in critically ill patients receiving continuous hemodialysis filtration. Yakugaku Zasshi.

[369] Maseda E, Grau S, Villagran MJ, Hernandez-Gancedo C, Lopez-Tofiño A, Roberts JA (2014). Micafungin pharmacokinetic/pharmacodynamic adequacy for the treatment of invasive candidiasis in critically ill patients on continuous venovenous haemofiltration. J Antimicrob Chemother.

[370] Konishi H, Fukushima K, Saotome T, Hamamoto T, Eguchi Y, Sudo M (2010). Impact of plasma exchange on pharmacokinetic disposition of micafungin. Ther Apher Dial..

[371] Lempers VJ, Schouten JA, Hunfeld NG, Colbers A, van Leeuwen HJ, Burger DM (2015). Altered micafungin pharmacokinetics in intensive care unit patients. Antimicrob Agents Chemother.

[372] Jullien V, Azoulay E, Schwebel C, Le Saux T, Charles PE, Cornet M (2017). Population pharmacokinetics of micafungin in ICU patients with sepsis and mechanical ventilation. J Antimicrob Chemother.

[373] Autmizguine J, Hornik CP, Benjamin DK, Brouwer KL, Hupp SR, Cohen-Wolkowiez M (2016). Pharmacokinetics and safety of micafungin in infants supported with extracorporeal membrane oxygenation. Pediatr Infect Dis J..

[374] Maseda E, Grau S, Hernandez-Gancedo C, Suarez-de-la-Rica A, Aguilar L, Gilsanz F (2015). Pharmacokinetics/pharmacodynamics of micafungin in a surgical critically ill patient during extracorporeal carbon dioxide removal and continuous renal replacement therapy. J Crit Care.

[375] Undre N, Stevenson P, Baraldi E (2012). Pharmacokinetics of micafungin in HIV positive patients with confirmed esophageal candidiasis. Eur J Drug Metab Pharmacokinet.

[376] Niwa T, Yokota Y, Tokunaga A, Yamato Y, Kagayama A, Fujiwara T (2004). Tissue distribution after intravenous dosing of micafungin, an antifungal drug, to rats. Biol Pharm Bull.

[377] Groll AH, Mickiene D, Petraitis V, Petraitiene R, Ibrahim KH, Piscitelli SC (2001). Compartmental pharmacokinetics and tissue distribution of the antifungal echinocandin lipopeptide micafungin (FK463) in rabbits. Antimicrob Agents Chemother.

[378] Nicasio AM, Tessier PR, Nicolau DP, Knauft RF, Russomanno J, Shore E (2009). Bronchopulmonary disposition of micafungin in healthy adult volunteers. Antimicrob Agents Chemother.

[379] Walsh TJ, Goutelle S, Jelliffe RW, Golden JA, Little EA, DeVoe C (2010). Intrapulmonary pharmacokinetics and pharmacodynamics of micafungin in adult lung transplant patients. Antimicrob Agents Chemother.

[380] Lat A, Thompson GR, Rinaldi MG, Dorsey SA, Pennick G, Lewis JS (2010). Micafungin concentrations from brain tissue and pancreatic pseudocyst fluid. Antimicrob Agents Chemother.

[381] Yamada N, Kumada K, Kishino S, Mochizuki N, Ohno K, Ogura S (2011). Distribution of micafungin in the tissue fluids of patients with invasive fungal infections. J Infect Chemother..

[382] Maruyama T, Takei Y, Gabazza EC, Morser J, Taguchi O (2009). Different bile concentration of micafungin and itraconazole in a patient with candidal cholecystitis. J Infect.

[383] Sasaki J, Yamanouchi S, Kudo D, Endo T, Nomura R, Takuma K (2012). Micafungin concentrations in the plasma and burn eschar of severely burned patients. Antimicrob Agents Chemother.

[384] Sasaki J, Yamanouchi S, Sato Y, Abe S, Shinozawa Y, Kishino S (2014). Penetration of micafungin into the burn eschar in patients with severe burns. Eur J Drug Metab Pharmacokinet.

[385] García-de-Lorenzo A, Luque S, Grau S, Agrifoglio A, Cachafeiro L, Herrero E (2016). Comparative population plasma and tissue pharmacokinetics of micafungin in critically ill patients with severe burn injuries and patients with complicated intra-abdominal infection. Antimicrob Agents Chemother.

[386] Mochizuki K, Suemori S, Udo K, Komori S, Ohkusu K, Yamada N (2011). Intraocular penetration of micafungin in patient with *Candida albicans* endophthalmitis. J Ocul Pharmacol Ther.

[387] Mochizuki K, Sawada A, Suemori S, Kawakami H, Niwa Y, Kondo Y (2013). Intraocular penetration of intravenous micafungin in inflamed human eyes. Antimicrob Agents Chemother.

[388] Barchiesi F, Orsetti E, Gesuita R, Skrami E, Manso E, Group CS (2016). Epidemiology, clinical characteristics, and outcome of candidemia in a tertiary referral center in Italy from 2010 to 2014. Infection.

[389] Perfect JR, Dismukes WE, Dromer F, Goldman DL, Graybill JR, Hamill RJ (2010). Clinical practice guidelines for the management of cryptococcal disease: 2010 update by the infectious diseases society of america. Clin Infect Dis.

[390] Carrillo-Muñoz AJ, Finquelievich J, Tur-Tur C, Eraso E, Jauregizar N, Quindós G (2014). Combination antifungal therapy: a strategy for the management of invasive fungal infections. Rev Esp Quimioter..

[391] Hatipoglu N, Hatipoglu H (2013). Combination antifungal therapy for invasive fungal infections in children and adults. Expert Rev Anti Infect Ther..

[392] Kontoyiannis DP, Lewis RE (2004). Toward more effective antifungal therapy: the prospects of combination therapy. Br J Haematol.

[393] Groll AH, Piscitelli SC, Walsh TJ (1998). Clinical pharmacology of systemic antifungal agents: a comprehensive review of agents in clinical use, current investigational compounds, and putative targets for antifungal drug development. Adv Pharmacol.

[394] Mukherjee PK, Sheehan DJ, Hitchcock CA, Ghannoum MA (2005). Combination treatment of invasive fungal infections. Clin Microbiol Rev.

[395] Foucquier J, Guedj M (2015). Analysis of drug combinations: current methodological landscape. Pharmacol Res Perspect..

[396] Koizumi Y, Iwami S (2014). Mathematical modeling of multi-drugs therapy: a challenge for determining the optimal combinations of antiviral drugs. Theor Biol Med Model..

[397] Spitzer M, Robbins N, Wright GD (2016). Combinatorial strategies for combating invasive fungal infections. Virulence..

[398] Odds FC (2003). Synergy, antagonism, and what the chequerboard puts between them. J Antimicrob Chemother.

[399] Meletiadis J, Pournaras S, Roilides E, Walsh TJ (2010). Defining fractional inhibitory concentration index cutoffs for additive interactions based on self-drug additive combinations, Monte Carlo simulation analysis, and in vitro-in vivo correlation data for antifungal drug combinations against Aspergillus fumigatus. Antimicrob Agents Chemother.

[400] Zhao L, Au JL, Wientjes MG (2010). Comparison of methods for evaluating drug–drug interaction. Front Biosci (Elite Ed)..

[401] Cantón E, Pemán J, Gobernado M, Viudes A, Espinel-Ingroff A (2005). Synergistic activities of fluconazole and voriconazole with terbinafine against four *Candida* species determined by checkerboard, time-kill, and Etest methods. Antimicrob Agents Chemother.

[402] Greco WR, Bravo G, Parsons JC (1995). The search for synergy: a critical review from a response surface perspective. Pharmacol Rev.

[403] Te Dorsthorst DT, Verweij PE, Meis JF, Punt NC, Mouton JW (2002). Comparison of fractional inhibitory concentration index with response surface modeling for characterization of in vitro interaction of antifungals against itraconazole-susceptible and -resistant *Aspergillus fumigatus* isolates. Antimicrob Agents Chemother.

[404] Zimmermann GR, Lehár J, Keith CT (2007). Multi-target therapeutics: when the whole is greater than the sum of the parts. Drug Discov Today..

[405] Cokol M, Chua HN, Tasan M, Mutlu B, Weinstein ZB, Suzuki Y (2011). Systematic exploration of synergistic drug pairs. Mol Syst Biol..

[406] Meletiadis J, Stergiopoulou T, O’Shaughnessy EM, Peter J, Walsh TJ (2007). Concentration-dependent synergy and antagonism within a triple antifungal drug combination against Aspergillus species: analysis by a new response surface model. Antimicrob Agents Chemother.

[407] Kirkpatrick WR, Perea S, Coco BJ, Patterson TF (2002). Efficacy of caspofungin alone and in combination with voriconazole in a Guinea pig model of invasive aspergillosis. Antimicrob Agents Chemother.

[408] Manavathu EK, Alangaden GJ, Chandrasekar PH (2003). Differential activity of triazoles in two-drug combinations with the echinocandin caspofungin against *Aspergillus fumigatus*. J Antimicrob Chemother.

[409] Meletiadis J, te Dorsthorst DT, Verweij PE (2006). The concentration-dependent nature of in vitro amphotericin B-itraconazole interaction against *Aspergillus fumigatus*: isobolographic and response surface analysis of complex pharmacodynamic interactions. Int J Antimicrob Agents.

[410] Kontoyiannis DP, Lewis RE, Sagar N, May G, Prince RA, Rolston KV (2000). Itraconazole-amphotericin B antagonism in *Aspergillus fumigatus*: an E-test-based strategy. Antimicrob Agents Chemother.

[411] O’Shaughnessy EM, Meletiadis J, Stergiopoulou T, Demchok JP, Walsh TJ (2006). Antifungal interactions within the triple combination of amphotericin B, caspofungin and voriconazole against *Aspergillus* species. J Antimicrob Chemother.

[412] Santos JR, Gouveia LF, Taylor EL, Resende-Stoianoff MA, Pianetti GA, César IC (2012). Dynamic interaction between fluconazole and amphotericin B against *Cryptococcus gattii*. Antimicrob Agents Chemother.

[413] Meletiadis J, Petraitis V, Petraitiene R, Lin P, Stergiopoulou T, Kelaher AM (2006). Triazole-polyene antagonism in experimental invasive pulmonary aspergillosis: in vitro and in vivo correlation. J Infect Dis.

[414] Louie A, Kaw P, Banerjee P, Liu W, Chen G, Miller MH (2001). Impact of the order of initiation of fluconazole and amphotericin B in sequential or combination therapy on killing of *Candida albicans* in vitro and in a rabbit model of endocarditis and pyelonephritis. Antimicrob Agents Chemother.

[415] Lewis RE, Prince RA, Chi J, Kontoyiannis DP (2002). Itraconazole preexposure attenuates the efficacy of subsequent amphotericin B therapy in a murine model of acute invasive pulmonary aspergillosis. Antimicrob Agents Chemother.

[416] Rex JH, Pappas PG, Karchmer AW, Sobel J, Edwards JE, Hadley S (2003). A randomized and blinded multicenter trial of high-dose fluconazole plus placebo versus fluconazole plus amphotericin B as therapy for candidemia and its consequences in nonneutropenic subjects. Clin Infect Dis.

[417] Groll AH, Giri N, Petraitis V, Petraitiene R, Candelario M, Bacher JS (2000). Comparative efficacy and distribution of lipid formulations of amphotericin B in experimental *Candida albicans* infection of the central nervous system. J Infect Dis.

[418] Steinbach WJ, Perfect JR, Cabell CH, Fowler VG, Corey GR, Li JS (2005). A meta-analysis of medical versus surgical therapy for *Candida* endocarditis. J Infect.

[419] Voice RA, Bradley SF, Sangeorzan JA, Kauffman CA (1994). Chronic candidal meningitis: an uncommon manifestation of candidiasis. Clin Infect Dis.

[420] Panackal AA, Parisini E, Proschan M (2014). Salvage combination antifungal therapy for acute invasive aspergillosis may improve outcomes: a systematic review and meta-analysis. Int J Infect Dis..

[421] Garnacho-Montero J, Olaechea P, Alvarez-Lerma F, Alvarez-Rocha L, Blanquer J, Galván B (2013). Epidemiology, diagnosis and treatment of fungal respiratory infections in the critically ill patient. Rev Esp Quimioter..

[422] Koulenti D, Garnacho-Montero J, Blot S (2014). Approach to invasive pulmonary aspergillosis in critically ill patients. Curr Opin Infect Dis..

[423] Singh N, Limaye AP, Forrest G, Safdar N, Muñoz P, Pursell K (2006). Combination of voriconazole and caspofungin as primary therapy for invasive aspergillosis in solid organ transplant recipients: a prospective, multicenter, observational study. Transplantation.

[424] Garbati MA, Alasmari FA, Al-Tannir MA, Tleyjeh IM (2012). The role of combination antifungal therapy in the treatment of invasive aspergillosis: a systematic review. Int J Infect Dis..

[425] Blot S, Koulenti D, Dimopoulos G (2013). Invasive pulmonary aspergillosis in critically ill patients. Annual update in intensive care and emergency medicine.

[426] Marr KA, Boeckh M, Carter RA, Kim HW, Corey L (2004). Combination antifungal therapy for invasive aspergillosis. Clin Infect Dis.

[427] Jeans AR, Howard SJ, Al-Nakeeb Z, Goodwin J, Gregson L, Warn PA (2012). Combination of voriconazole and anidulafungin for treatment of triazole-resistant aspergillus fumigatus in an in vitro model of invasive pulmonary aspergillosis. Antimicrob Agents Chemother.

[428] Chandrasekar PH, Cutright JL, Manavathu EK (2004). Efficacy of voriconazole plus amphotericin B or micafungin in a guinea-pig model of invasive pulmonary aspergillosis. Clin Microbiol Infect.

[429] Kontoyiannis DP, Ratanatharathorn V, Young JA, Raymond J, Laverdière M, Denning DW (2009). Micafungin alone or in combination with other systemic antifungal therapies in hematopoietic stem cell transplant recipients with invasive aspergillosis. Transpl Infect Dis..

[430] Marr KA, Schlamm HT, Herbrecht R, Rottinghaus ST, Bow EJ, Cornely OA (2015). Combination antifungal therapy for invasive aspergillosis: a randomized trial. Ann Intern Med.

[431] Johnson LB, Kauffman CA (2003). Voriconazole: a new triazole antifungal agent. Clin Infect Dis.

[432] Belanger ES, Yang E, Forrest GN (2015). Combination antifungal therapy: when, where, and why. Curr Clin Micro Rpt.

[433] Perfect JR, Bicanic T (2015). Cryptococcosis diagnosis and treatment: what do we know now. Fungal Genet Biol.

[434] Bennett JE, Dismukes WE, Duma RJ, Medoff G, Sande MA, Gallis H (1979). A comparison of amphotericin B alone and combined with flucytosine in the treatment of cryptococcal meningitis. N Engl J Med.

[435] Day JN, Chau TT, Lalloo DG (2013). Combination antifungal therapy for cryptococcal meningitis. N Engl J Med.

[436] Chayakulkeeree M, Perfect JR (2006). Cryptococcosis. Infect Dis Clin North Am..

[437] O’Connor L, Livermore J, Sharp AD, Goodwin J, Gregson L, Howard SJ (2013). Pharmacodynamics of liposomal amphotericin B and flucytosine for cryptococcal meningoencephalitis: safe and effective regimens for immunocompromised patients. J Infect Dis.

[438] Maziarz EK, Perfect JR (2016). Cryptococcosis. Infect Dis Clin North Am.

[439] Jackson AT, Nussbaum JC, Phulusa J, Namarika D, Chikasema M, Kanyemba C (2012). A phase II randomized controlled trial adding oral flucytosine to high-dose fluconazole, with short-course amphotericin B, for cryptococcal meningitis. AIDS..

[440] Milefchik E, Leal MA, Haubrich R, Bozzette SA, Tilles JG, Leedom JM (2008). Fluconazole alone or combined with flucytosine for the treatment of AIDS-associated cryptococcal meningitis. Med Mycol.

[441] Ibrahim AS, Bowman JC, Avanessian V, Brown K, Spellberg B, Edwards JE (2005). Caspofungin inhibits *Rhizopus oryzae* 1,3-beta-D-glucan synthase, lowers burden in brain measured by quantitative PCR, and improves survival at a low but not a high dose during murine disseminated zygomycosis. Antimicrob Agents Chemother.

[442] Reed C, Bryant R, Ibrahim AS, Edwards J, Filler SG, Goldberg R (2008). Combination polyene-caspofungin treatment of rhino-orbital-cerebral mucormycosis. Clin Infect Dis.

[443] Pagano L, Cornely OA, Busca A, Caira M, Cesaro S, Gasbarrino C (2013). Combined antifungal approach for the treatment of invasive mucormycosis in patients with hematologic diseases: a report from the SEIFEM and FUNGISCOPE registries. Haematologica.

[444] Sheybani F, Naderi HR, Sarvghad M, Ghabouli M, Arian M (2015). How should we manage a patient with invasive mucoromycosis who develops life-threatening reaction to amphotericin B? Report of two cases and literature review. Med Mycol Case Rep..

